# Dataset of propylene carbonate based liquid electrolyte mixtures for sodium-ion cells

**DOI:** 10.1016/j.dib.2021.107775

**Published:** 2021-12-30

**Authors:** Andreas Hofmann, Zhengqi Wang, Sebastian Pinto Bautista, Marcel Weil, Freya Müller, Robert Löwe, Luca Schneider, Ijaz Ul Mohsin, Thomas Hanemann

**Affiliations:** a Karlsruher Institut für Technologie, Institut für Angewandte Materialien (IAM), Herrmann-von-Helmholtz Platz 1, Eggenstein-Leopoldshafen 76344, Germany; b Department of Microsystems Engineering, University of Freiburg, Georges-Köhler-Allee 102, Freiburg D-79110, Germany; c Karlsruher Institut für Technologie, Institut für Technikfolgenabschätzung und Systemanalyse (ITAS), Postfach 3640, Karlsruhe 76021, Germany; d Helmholtz-Institut Ulm für Elektrochemische Energiespeicherung (HIU), Helmholtzstraße 11, Ulm 89081, Germany

**Keywords:** Density data, Viscosity data, Conductivity data, Gas chromatography dataset, Life cycle analysis (LCA) data

## Abstract

In this manuscript, we present rheology, ionic conductivity, density, chromatography, and life cycle analysis data on the PC+X electrolyte system with and without LiClO_4_. In particular, the data are presented in contact with Na surfaces. In this case, photographic images of electrolyte-sodium mixtures are also shown. The data was analyzed using OriginPro software to visualize it in an appropriate manner. In our view, the data serve as comparative values, form a basis of a chromatography analysis and are also valuable for modeling. The analysis of the data is presented in the manuscript “Comprehensive characterization of propylene carbonate based liquid electrolyte mixtures for sodium-ion cells” [1].

## Specifications Table

**Table:** 

Subject	Chemistry
Specific subject area	Analytical Chemistry and Electrochemistry
Type of data	Table Image Figure
How the data were acquired	The data were acquired via • density meter (Anton Paar DMA 4500M) • rheometer (Malvern Gemini HR Nano) • electrochemical workstation (Zahner Zennium IM6) • gas chromatography coupled with mass spectroscopy (Perkin Elmer Clarus 690 including a SQ8T mass spectrometer) • Advanced Electrolyte Model (AEM, Idaho National Laboratory) • Data are taken from www.echa.eu. An overview for each specific compound is provided in Table 12 (supporting information)
Data format	Raw Analyzed Filtered
Description of data collection	The data were collected by measurements as well as data analysis and software approach.
Data source location	All data are provided in the manuscript. The data was received from the following address: • Institution: Karlsruhe Institute of Technology • City/Town/Region: 76,344 Eggenstein-Leopoldshafen • Country: Germany
Data accessibility	With the article Primary data for the LCA overview (Table 9) were taken from following primary data sources: [2], [3], [4], [5], [6]
Related research article	A. Hofmann, Z. Wang, S. P. Bautista, M. Weil, F. Müller, R. Löwe, L. Schneider, I. U. Mohsin, T. Hanemann, Comprehensive characterization of propylene carbonate based liquid electrolyte mixtures for sodium-ion cells, Electrochimica Acta., https://doi.org/10.1016/j.electacta.2021.139670[1]

## Value of the Data

• The data support the images and figures shown in Ref. [1]. The GC data additionally helps the reader by substance identification. The data can be used for simulation and modeling of electrolyte properties
• Experimental as well as theoretic researches can profit from the data by using them for the described electrolytes
• The raw data can be used directly for electrolyte research. Additionally, the experimental data may be a set of data for modeling and/or simulation of electrolyte properties

## 1 Data Description

**Fig. 1:**
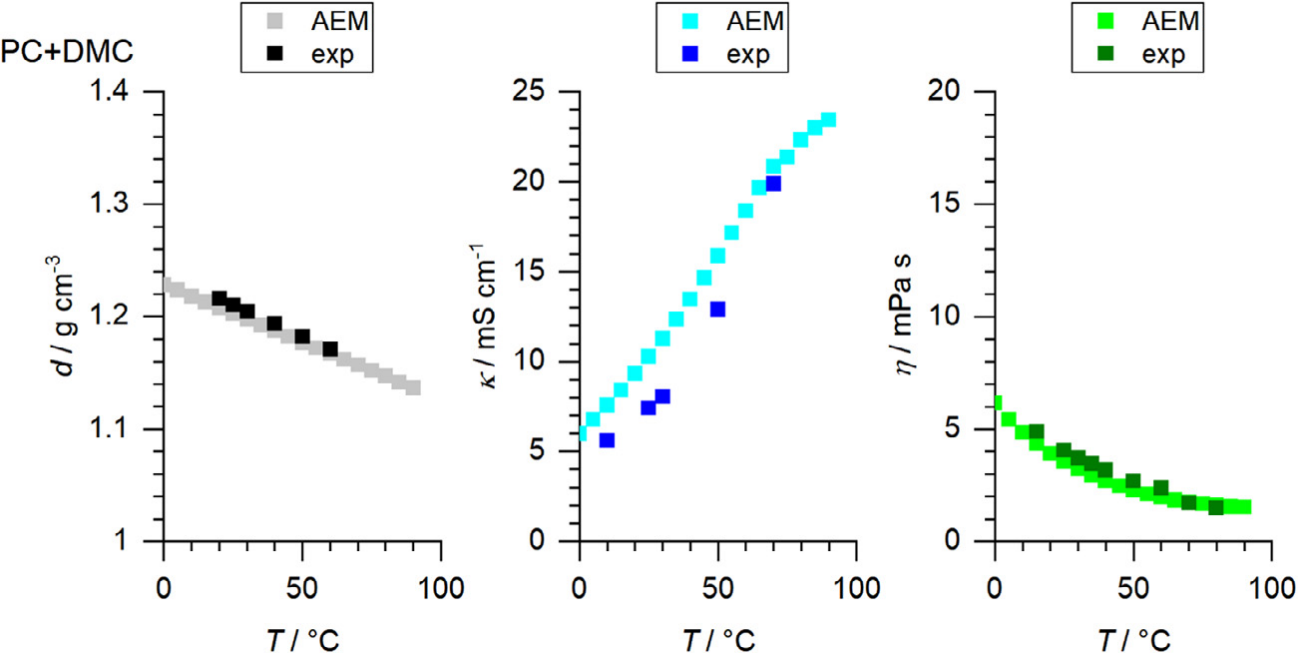
Temperature-dependent density (left-hand side), conductivity (middle) and viscosity (right-hand side) values of 1 M NaClO_4_ in PC+DMC.

**Fig. 2:**
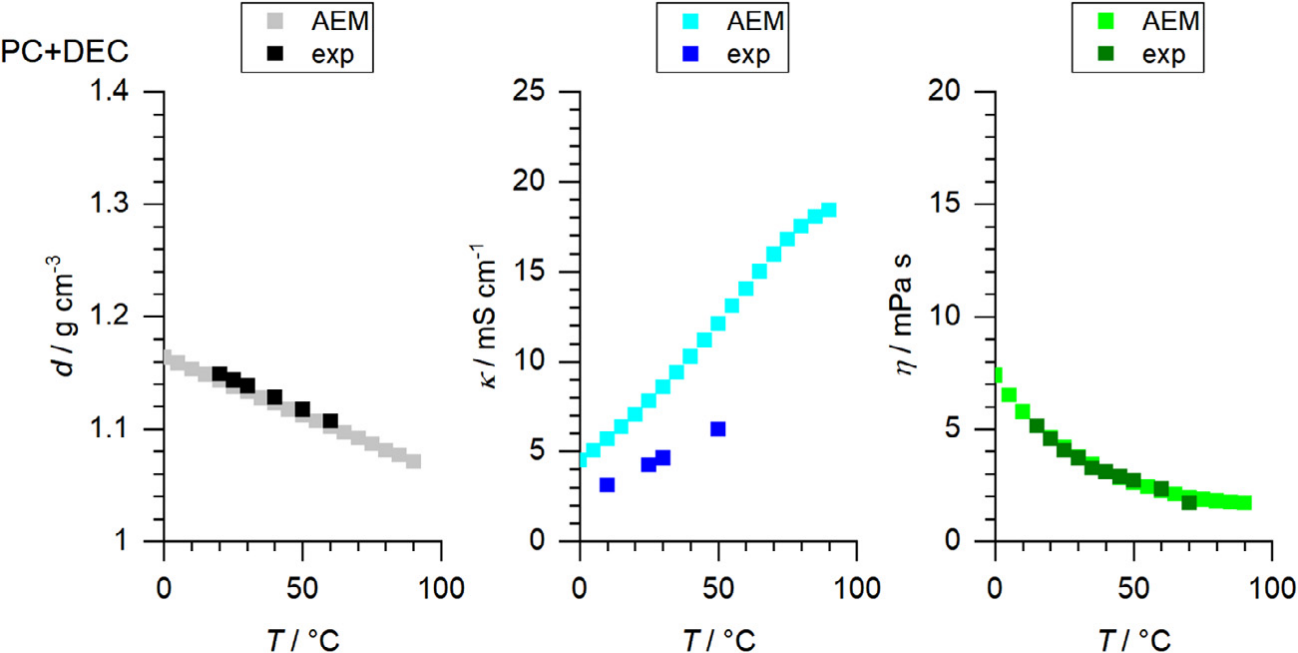
Temperature-dependent density (left-hand side), conductivity (middle) and viscosity (right-hand side) values of 1 M NaClO_4_ in PC+DEC.

**Fig. 3:**
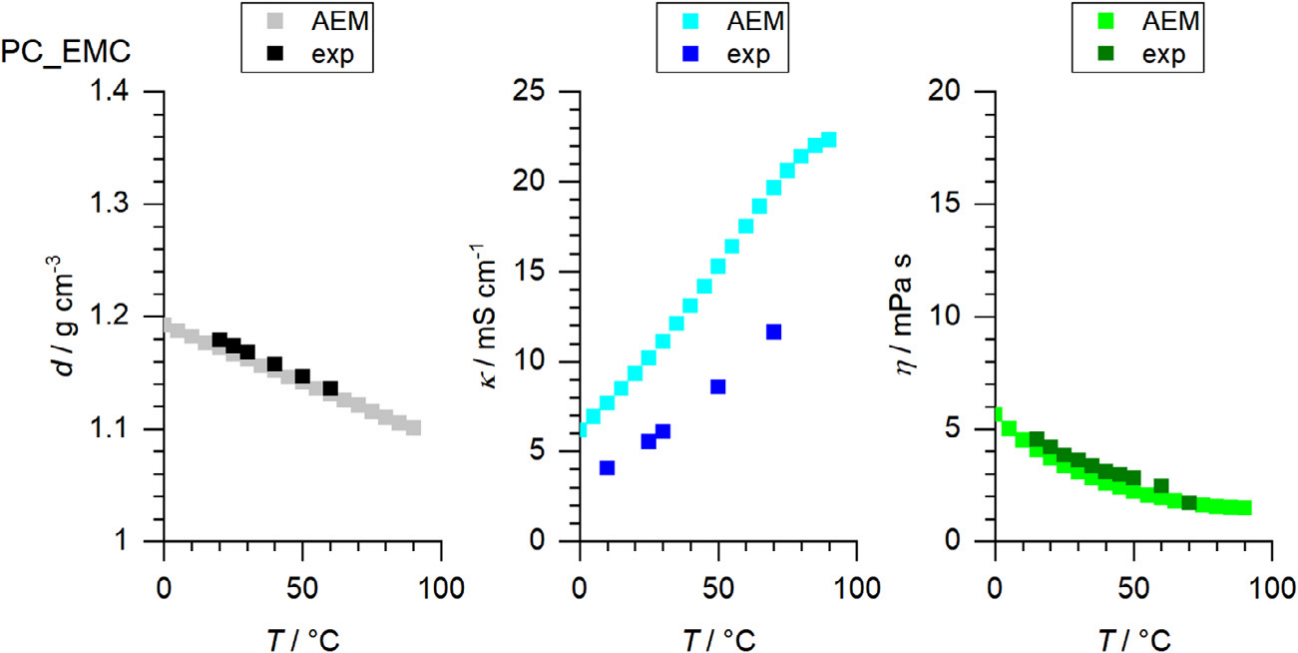
Temperature-dependent density (left-hand side), conductivity (middle) and viscosity (right-hand side) values of 1 M NaClO_4_ in PC+EMC.

**Fig. 4:**
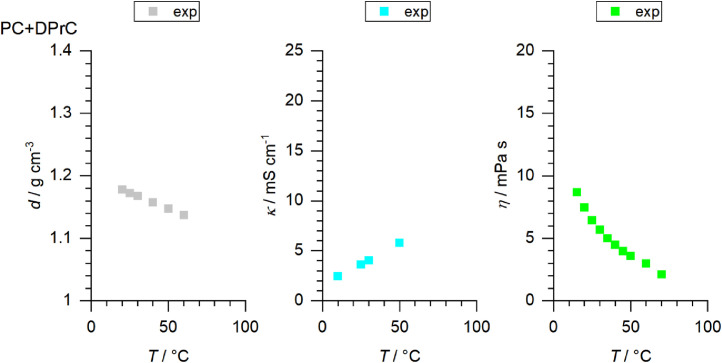
Temperature-dependent density (left-hand side), conductivity (middle) and viscosity (right-hand side) values of 1 M NaClO_4_ in PC+DPrC.

**Fig. 5:**
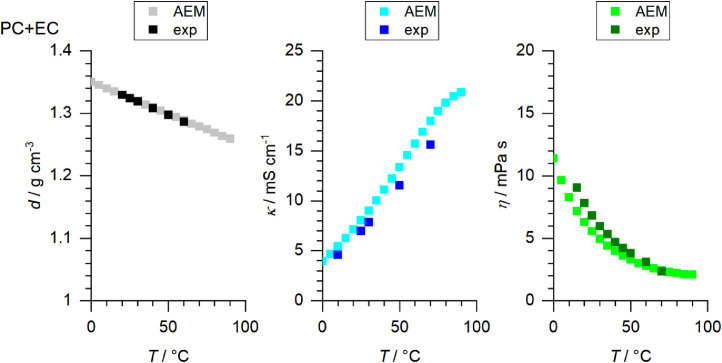
Temperature-dependent density (left-hand side), conductivity (middle) and viscosity (right-hand side) values of 1 M NaClO_4_ in PC+EC.

**Fig. 6:**
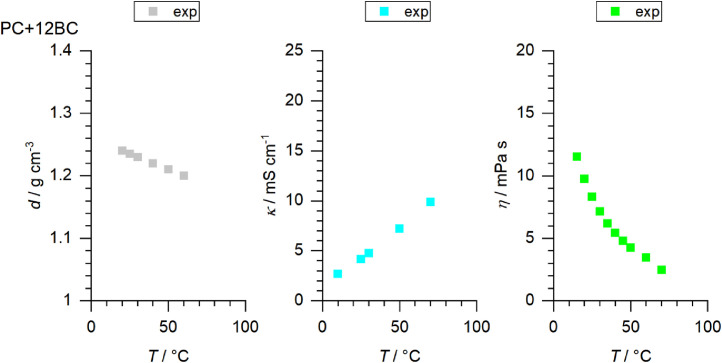
Temperature-dependent density (left-hand side), conductivity (middle) and viscosity (right-hand side) values of 1 M NaClO_4_ in PC+12BC.

**Fig. 7:**
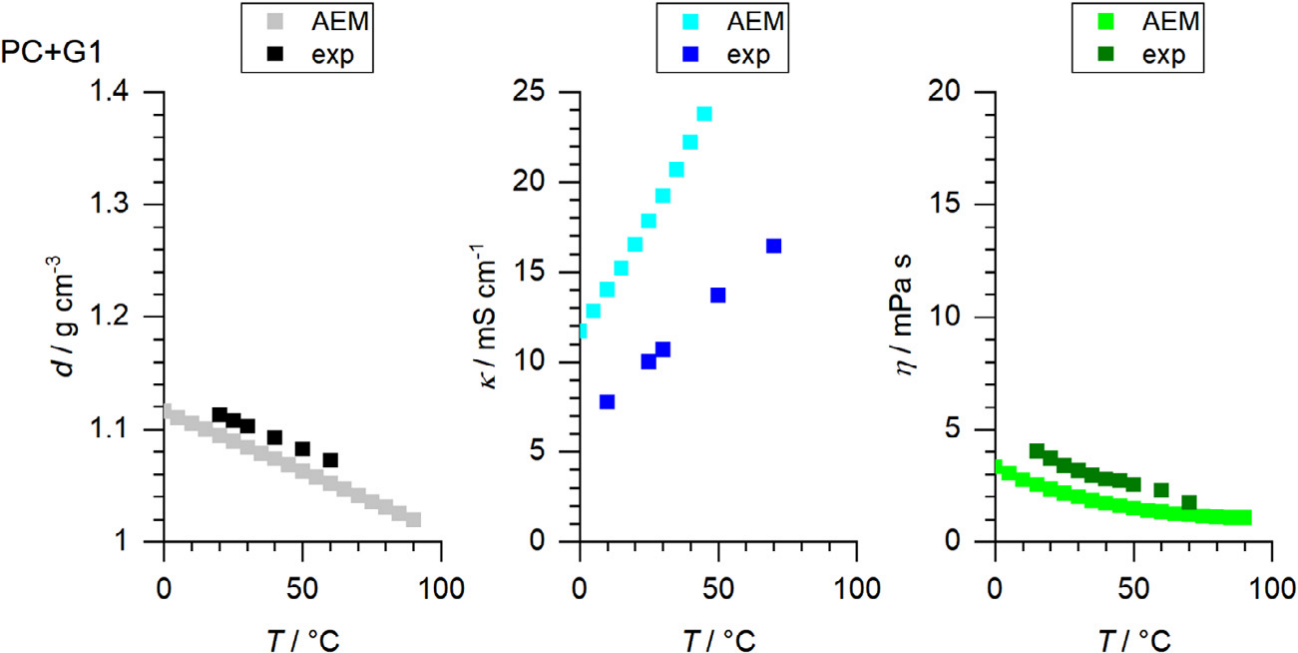
Temperature-dependent density (left-hand side), conductivity (middle) and viscosity (right-hand side) values of 1 M NaClO_4_ in PC+G1.

**Fig. 8:**
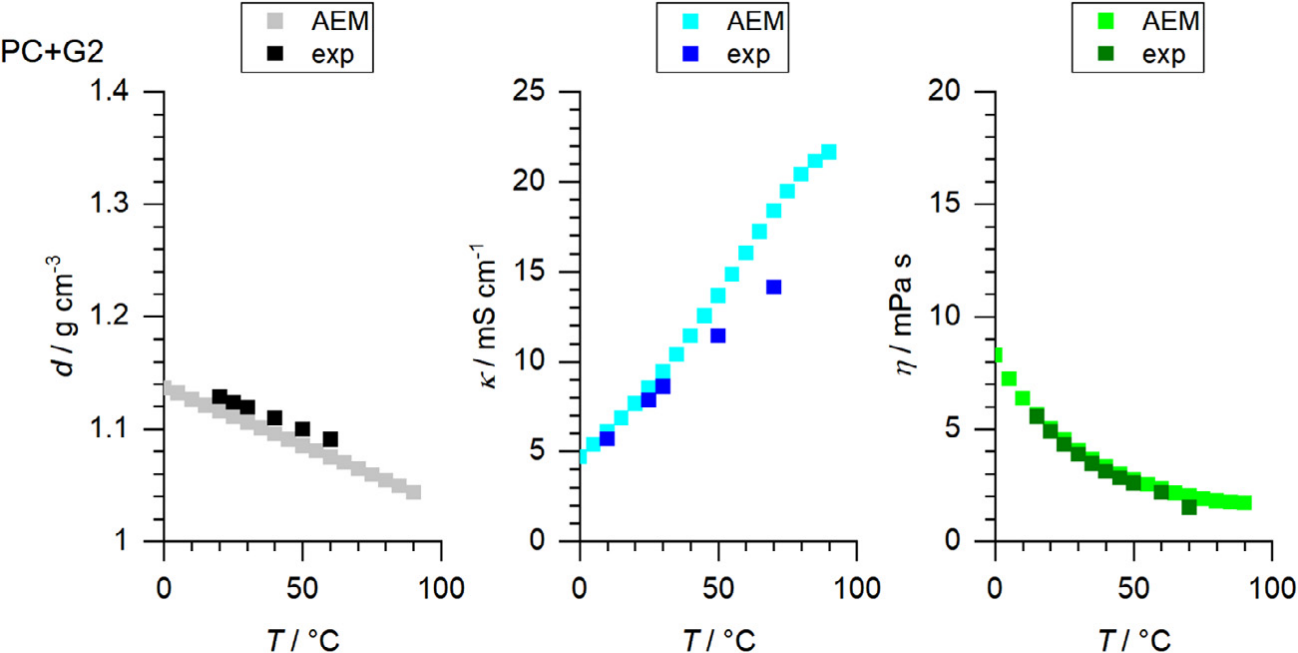
Temperature-dependent density (left-hand side), conductivity (middle) and viscosity (right-hand side) values of 1 M NaClO_4_ in PC+G2.

**Fig. 9:**
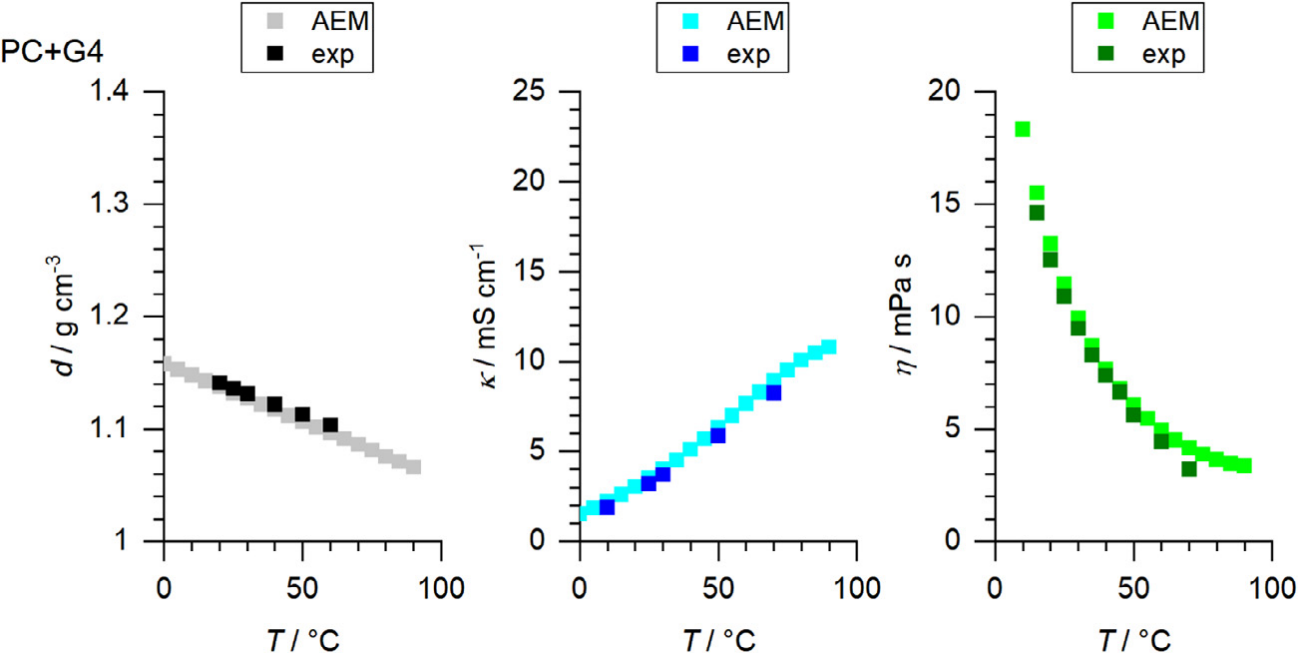
Temperature-dependent density (left-hand side), conductivity (middle) and viscosity (right-hand side) values of 1 M NaClO_4_ in PC+G4.

**Fig. 10:**
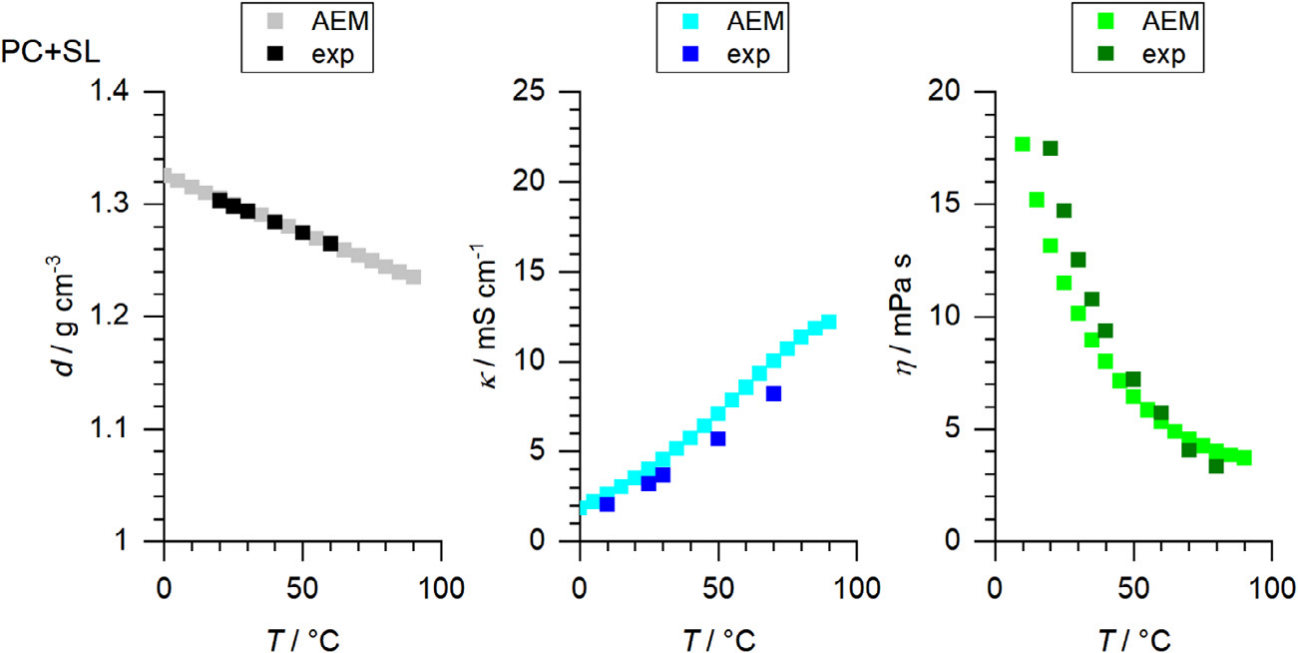
Temperature-dependent density (left-hand side), conductivity (middle) and viscosity (right-hand side) values of 1 M NaClO_4_ in PC+SL.

**Fig. 11:**
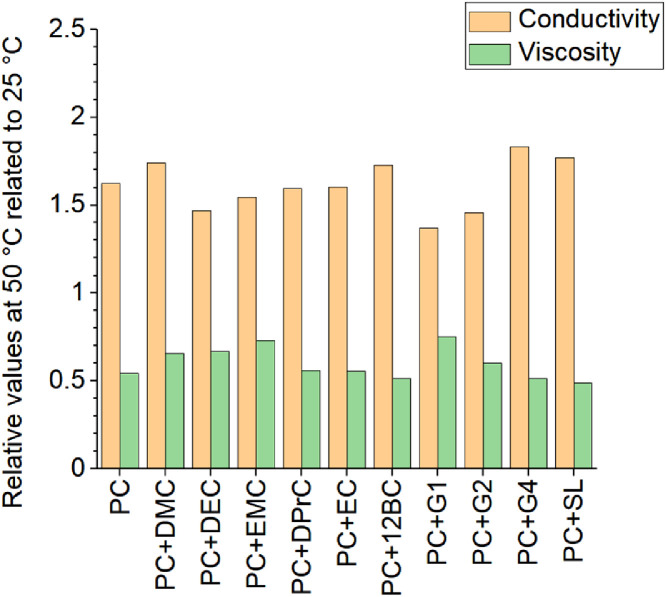
Relative modification of conductivity and viscosity with temperature increase from 25 °C to 50 °C.

Figs. 1 to 10 show the temperature dependent values of the density, viscosity and conductivity of the PC based electrolytes. Calculated values (mentioned as “AEM”) and experimental values (mentioned as “exp”) are plotted for a direct comparison. All specified values (calculated values, AEM) of the individual plots are listed in Tables 1a - 1i (see supporting information), namely the data of Fig. 1 are listed in Table 1b (in Table 1a, reference is made to Fig. 2 in the research article [1]), the data of Fig. 2 are listed in Table 1c, the data of Fig. 3 are listed in Table 1d, the data of Fig. 5 are listed in Table 1e, the data of Fig. 7 are listed in Table 1f, the data of Fig. 8 are listed in Table 1g, the data of Fig. 9 are listed in Table 1h and the data of Fig. 10 are listed in Table 1i (no calculations were made for Figs. 4 and 6). Experimental as well as calculated values of the density, conductivity and sodium diffusion coefficients at *T* = 25 °C are provided in Table 2a (supporting information), whereas viscosity values and Arrhenius flow characteristics are listed in Table 2b (supporting information) for all mixtures. Temperature dependent values (experimental) of the specific conductivity are provided in Table 3a, experimental values of the dynamic viscosity are listed in Table 3b and experimental density values are provided in Table 3c for all mixtures (all tables are provided in supporting information). In Fig. 11, a comparison is made of the conductivity and viscosity values between *T* = 25 °C and *T* = 50 °C. The raw data of Fig. 11 are listed in Table 4 (supporting information).

In Table 6, all mixtures are shown with and without NaClO_4_ addition before sodium addition and 20 days after sodium addition. In the research article [1], only exemplary individual substance classes are shown, while here all compounds are shown as an overview.

**Table 6:** Images of the samples after 20 d stored over sodium metal. This table is modified and completed from Table 3 in Ref. [1].

All gas chromatography (GC) chromatograms are shown in Figs. 12 to 21 (with exception of mixture PC+EC that is shown in Ref. [1]). All samples are directly compared with pure solvent as well as with the GC solvent methyl *tert*-butyl ether (MTBE). Since FID data are shown, a pronounced solvent signal is visible in all samples.

**Fig. 12:**
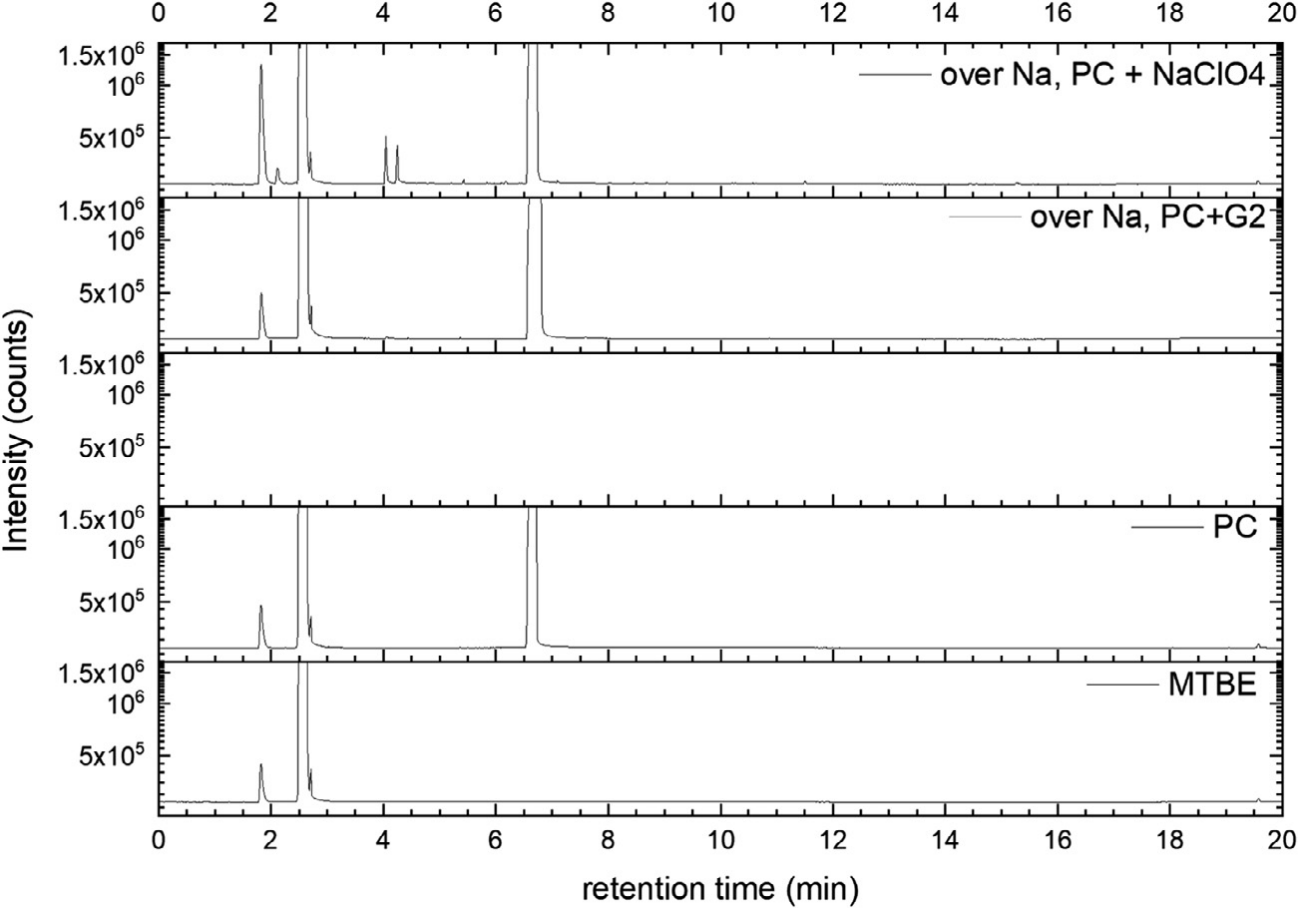
Chromatograms (raw data) (FID) of MTBE, PC, “PC + Na” and “PC + 1 M NaClO_4_ + Na”. Both sodium samples were measured after 150 days of storage.

**Fig. 13:**
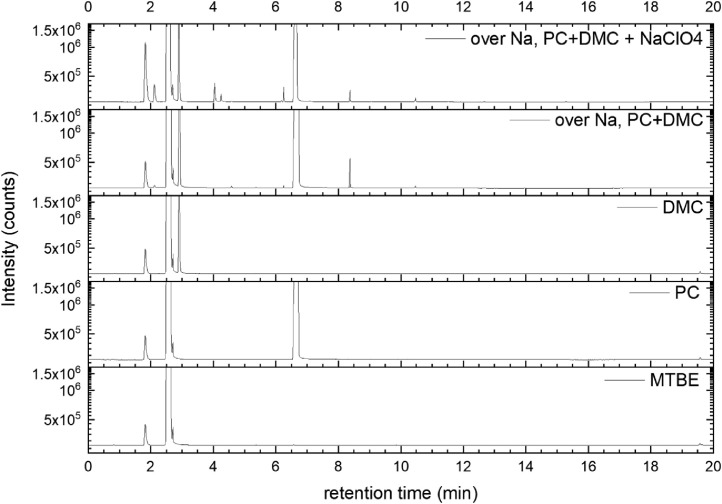
Chromatograms (raw data) (FID) of MTBE, PC, DMC, “PC + DMC + Na” and “PC + DMC + 1 M NaClO_4_ + Na”. Both sodium samples were measured after 150 days of storage.

**Fig. 14:**
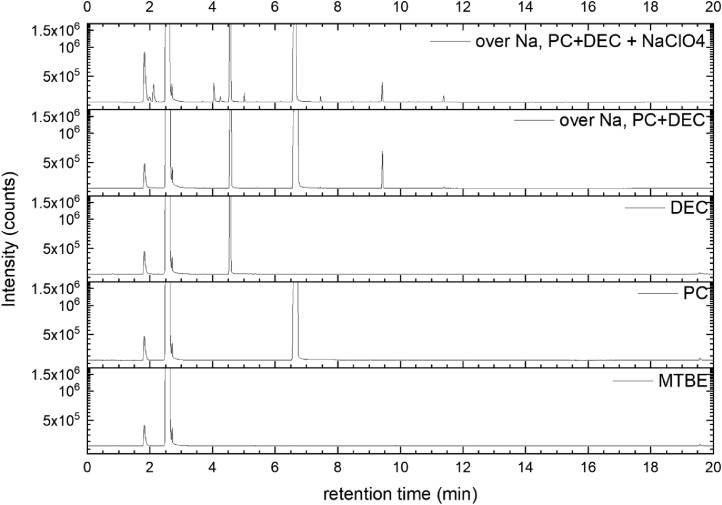
Chromatograms (raw data) (FID) of MTBE, PC, DEC, “PC + DEC + Na” and “PC + DEC + 1 M NaClO_4_ + Na”. Both sodium samples were measured after 150 days of storage.

**Fig. 15:**
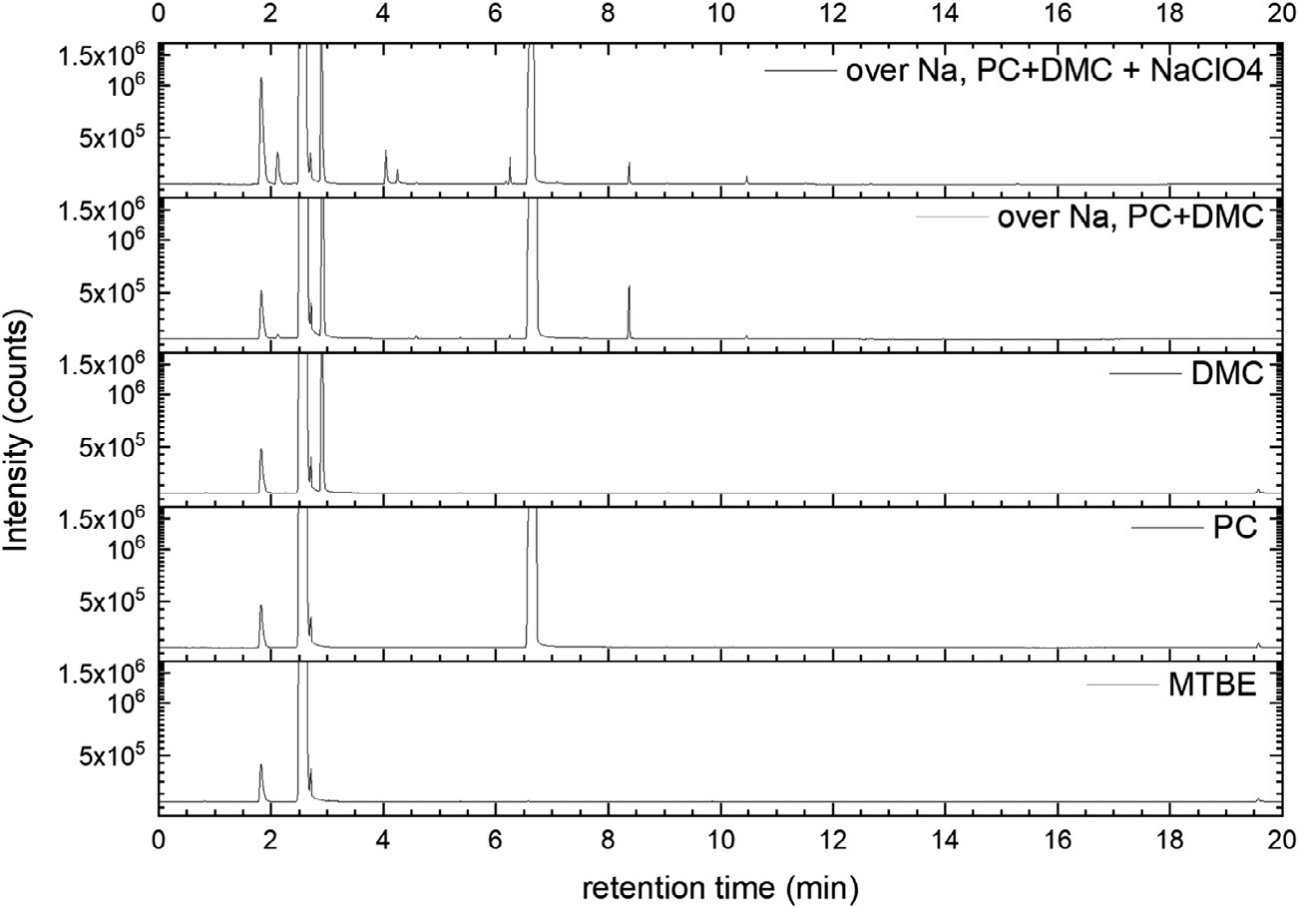
Chromatograms (raw data) (FID) of MTBE, PC, EMC, “PC + EMC + Na” and “PC + EMC + 1 M NaClO_4_ + Na”. Both sodium samples were measured after 150 days of storage.

**Fig. 16:**
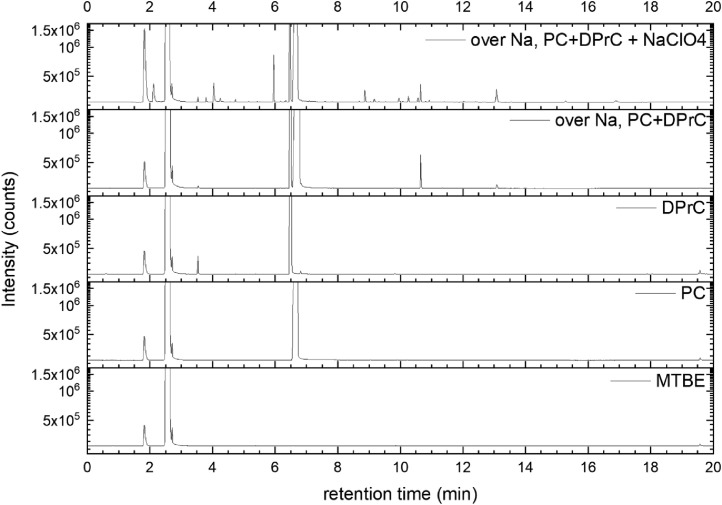
Chromatograms (raw data) (FID) of MTBE, PC, DPrC, “PC + DPrC + Na” and “PC + DPrC + 1 M NaClO_4_ + Na”. Both sodium samples were measured after 150 days of storage.

**Fig. 17:**
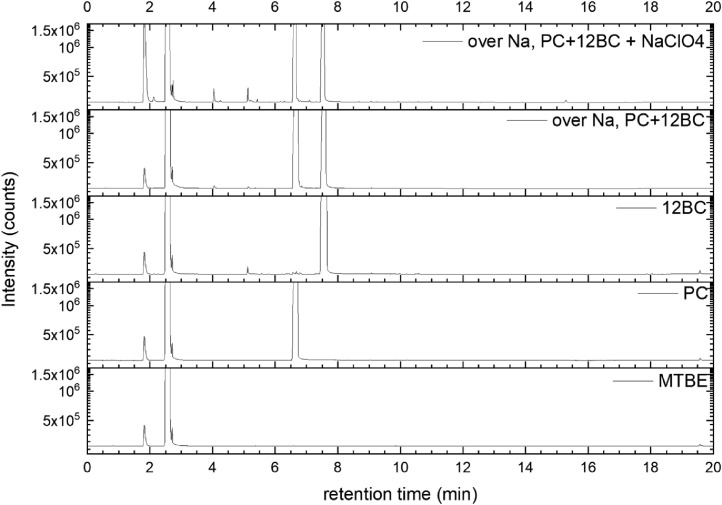
Chromatograms (raw data) (FID) of MTBE, PC, 12BC, “PC + 12BC + Na” and “PC + 12BC + 1 M NaClO_4_ + Na”. Both sodium samples were measured after 150 days of storage.

**Fig. 18:**
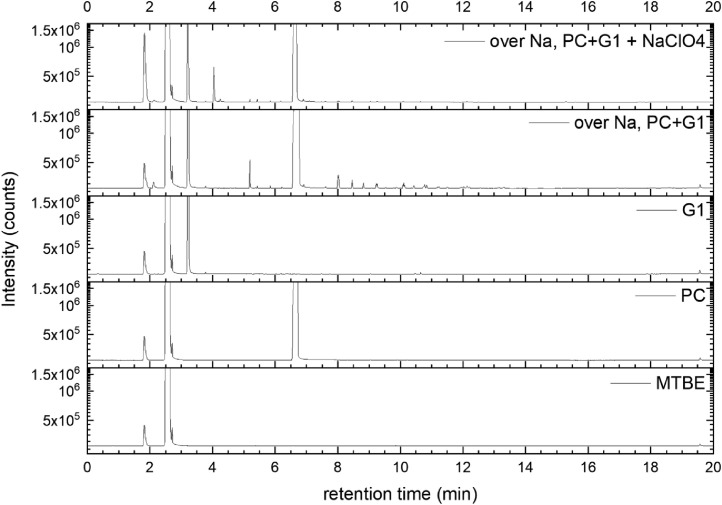
Chromatograms (raw data) (FID) of MTBE, PC, G1, “PC + G1 + Na” and “PC + G1 + 1 M NaClO_4_ + Na”. Both sodium samples were measured after 150 days of storage.

**Fig. 19:**
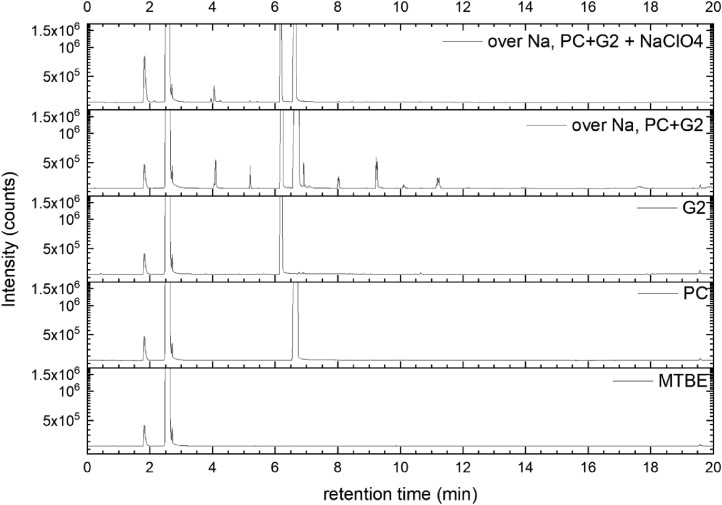
Chromatograms (raw data) (FID) of MTBE, PC, G2, “PC + G2 + Na” and “PC + G2 + 1 M NaClO_4_ + Na”. Both sodium samples were measured after 150 days of storage.

**Fig. 20:**
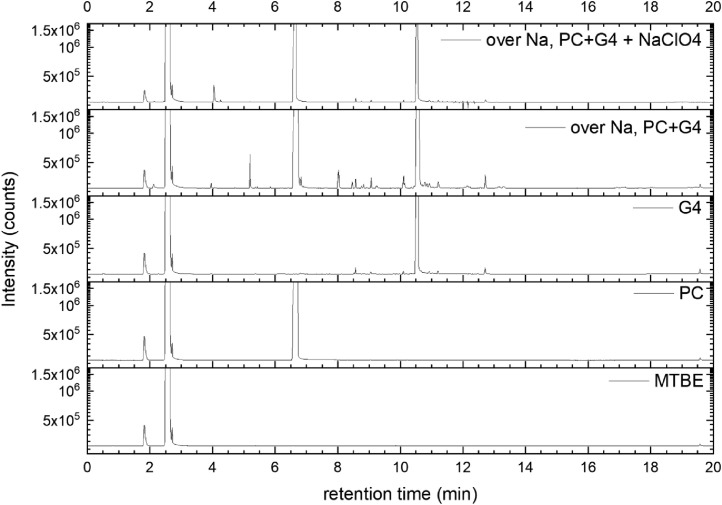
Chromatograms (raw data) (FID) of MTBE, PC, G4, “PC + G4 + Na” and “PC + G4 + 1 M NaClO_4_ + Na”. Both sodium samples were measured after 150 days of storage.

**Fig. 21:**
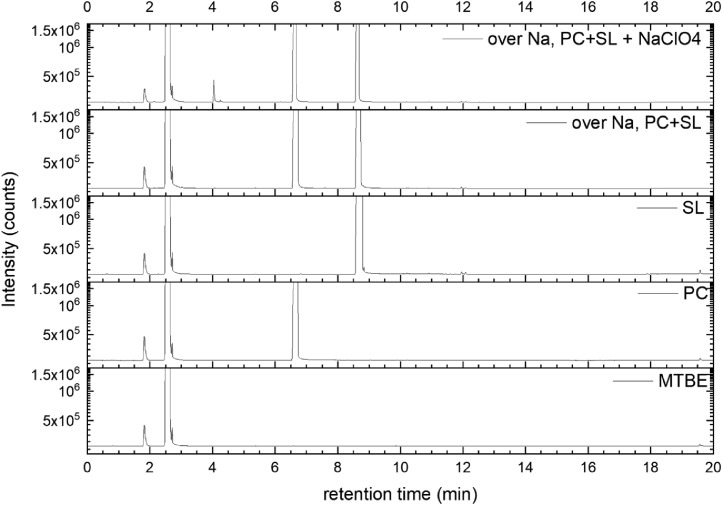
Chromatograms (raw data) (FID) of MTBE, PC, SL, “PC + SL + Na” and “PC + SL + 1 M NaClO_4_ + Na”. Both sodium samples were measured after 150 days of storage.

**Fig. 22:**
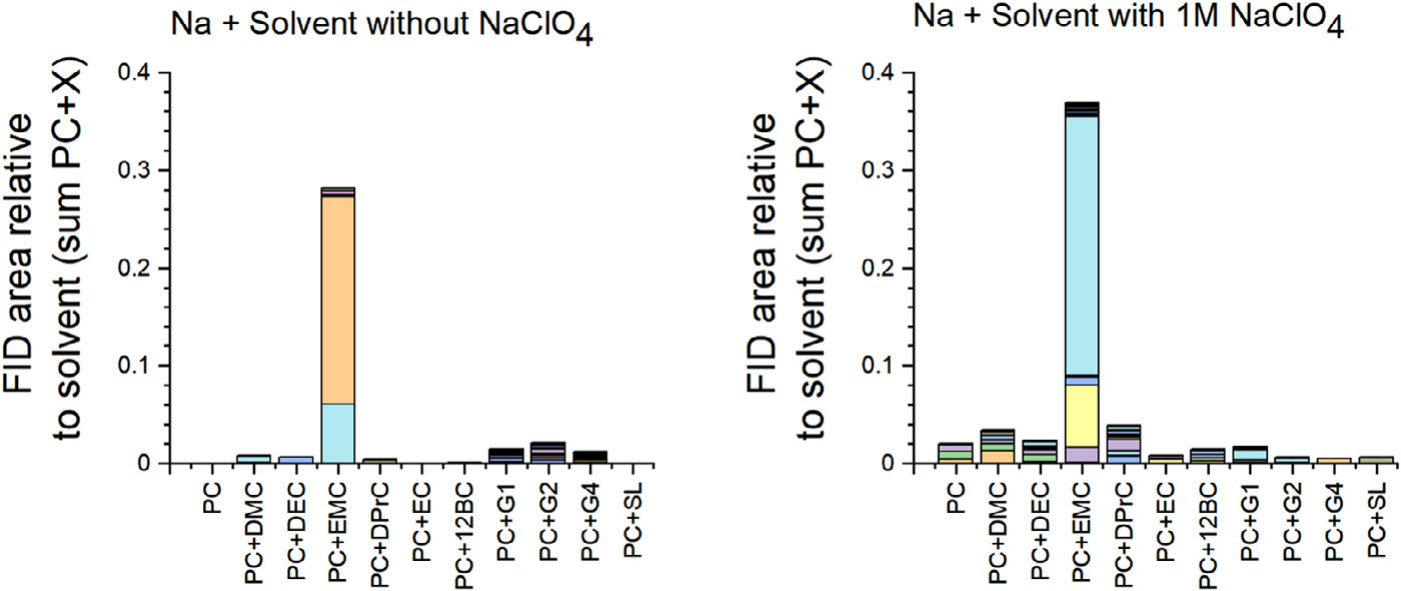
Gas chromatography results (FID) of electrolytes without (left-hand side) and with (right-hand side) NaClO_4_ salt over sodium metal after four months, modified from Fig. 5 in the research manuscript [1]. It should be noted that same colors of the bars do not represent the same substances, thus, each set of defragmentation products is individual for each mixture.

**Fig. 23:**
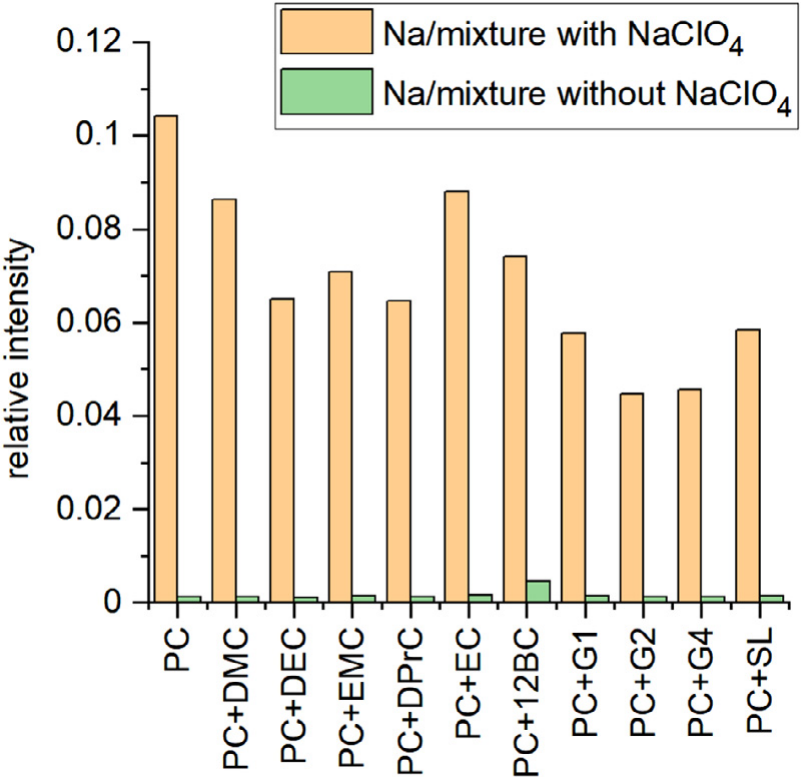
Gas chromatography results of electrolytes with and without NaClO_4_ salt including sodium metal after 4 months of storage. The relative intensity was calculated by referring the peak at 1.84 min (MS detector, mass extraction *m/z* = 44 from EI total fragmentation, CO_2_) relative to the peak at 6.80 min (PC, *m/z* = 87). Additionally, the PC intensity was corrected to the PC wt. content in the electrolyte.

A qualitative comparison of the individual peak areas is shown in Fig. 22 with and without sodium perchlorate including the formation of DMC and DEC from EMC. In Fig. 23, relative peak areas of CO_2_ against propylene carbonate (PC) are illustrated and compared between mixtures without and with sodium perchlorate. The data of Fig. 22 are listed in Table 5a (without NaClO_4_) and Table 5b (with NaClO_4_), and the data of Fig. 23 are shown in Table 5c (all in the supporting information).

**Fig. 24:**
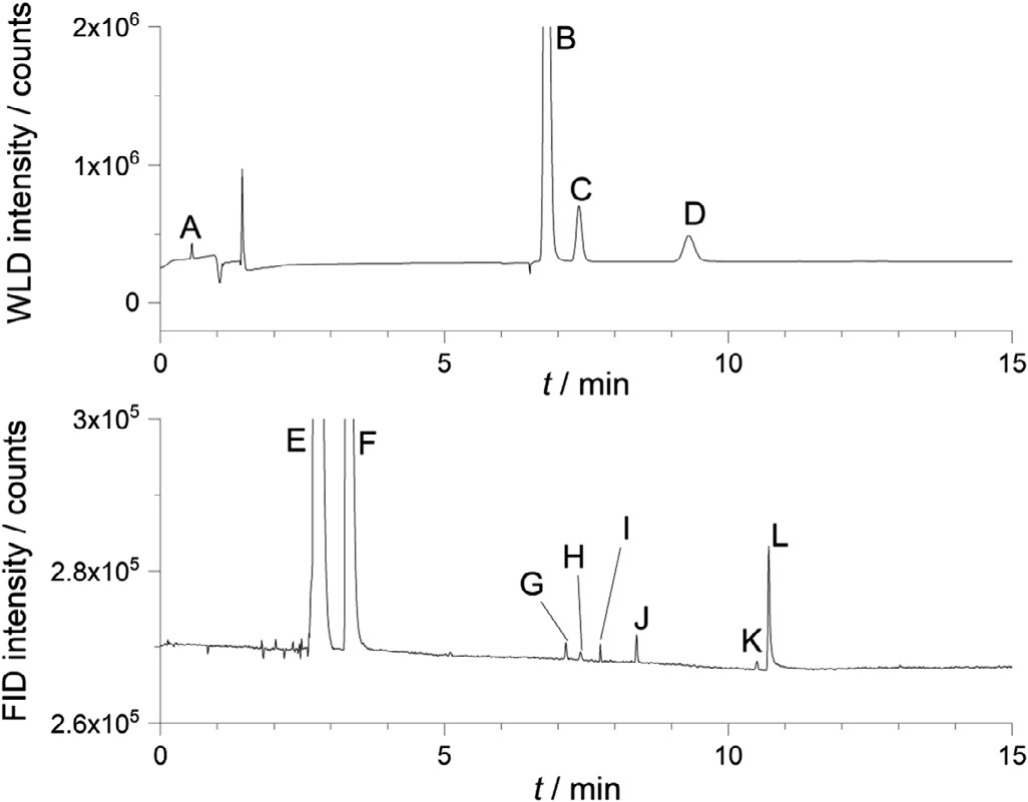
Gas analysis of the gas in the pat press cell with PC + 1 M NaClO_4_ + Na metal. Assignment to the peaks (For FID detector according to MS identification due to the splitted signal, see Fig. SI-24): *A* = H_2_, *B* = Ar/O_2_, C = N_2_, D = CO, *E* = O_2_/CO, *F* = propylene oxide, *G* = isopropyl isobutyrate (NIST), *H* = siloxane, *I* = no signal in MS, *J* = diisopropyl carbonate, *K* = siloxane, *L* = propylene carbonate.

A gas analysis from pure PC + NaClO_4_ electrolyte over sodium metal is shown in Fig. 24 and a short description of the individual peaks is provided in the caption text.

Impurities and reaction products that arise in the stored electrolyte (over Na), are mentioned in Tables 7 and 8 (supporting information). Both contain information about the detection of such substances with gas chromatography. Table 9 summarizes the results of the LCA for all impact categories (supporting information).

**Fig. 25:**
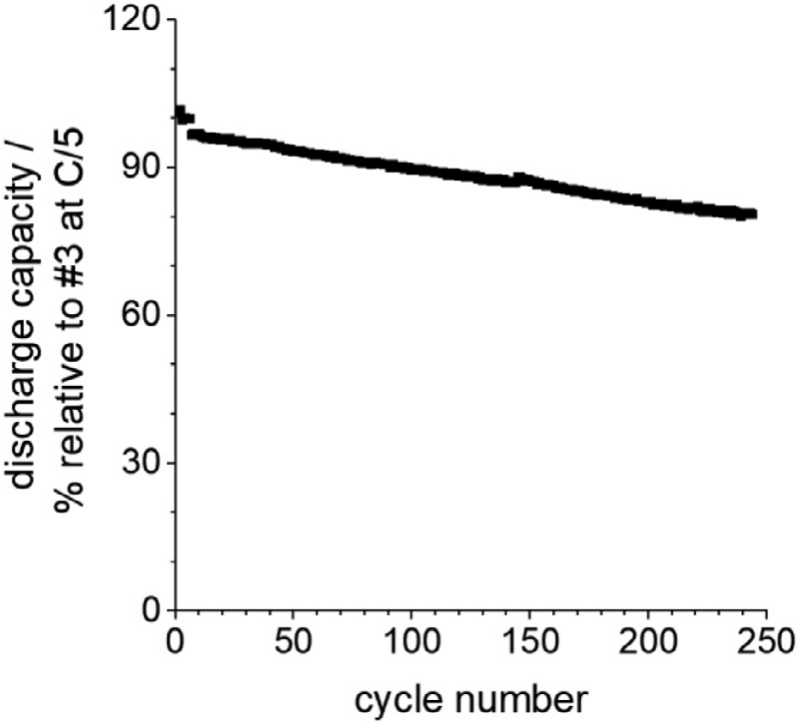
Cycling of NMO vs. HC cell with PC+1 M NaClO_4_ electrolyte without performance test for 250 cycles.

Fig. 25 shows the cycling behavior of sodium manganese oxide versus hard carbon over 250 cycles. The raw data are listed in Table 10 (supporting information).

**Fig. 26:**
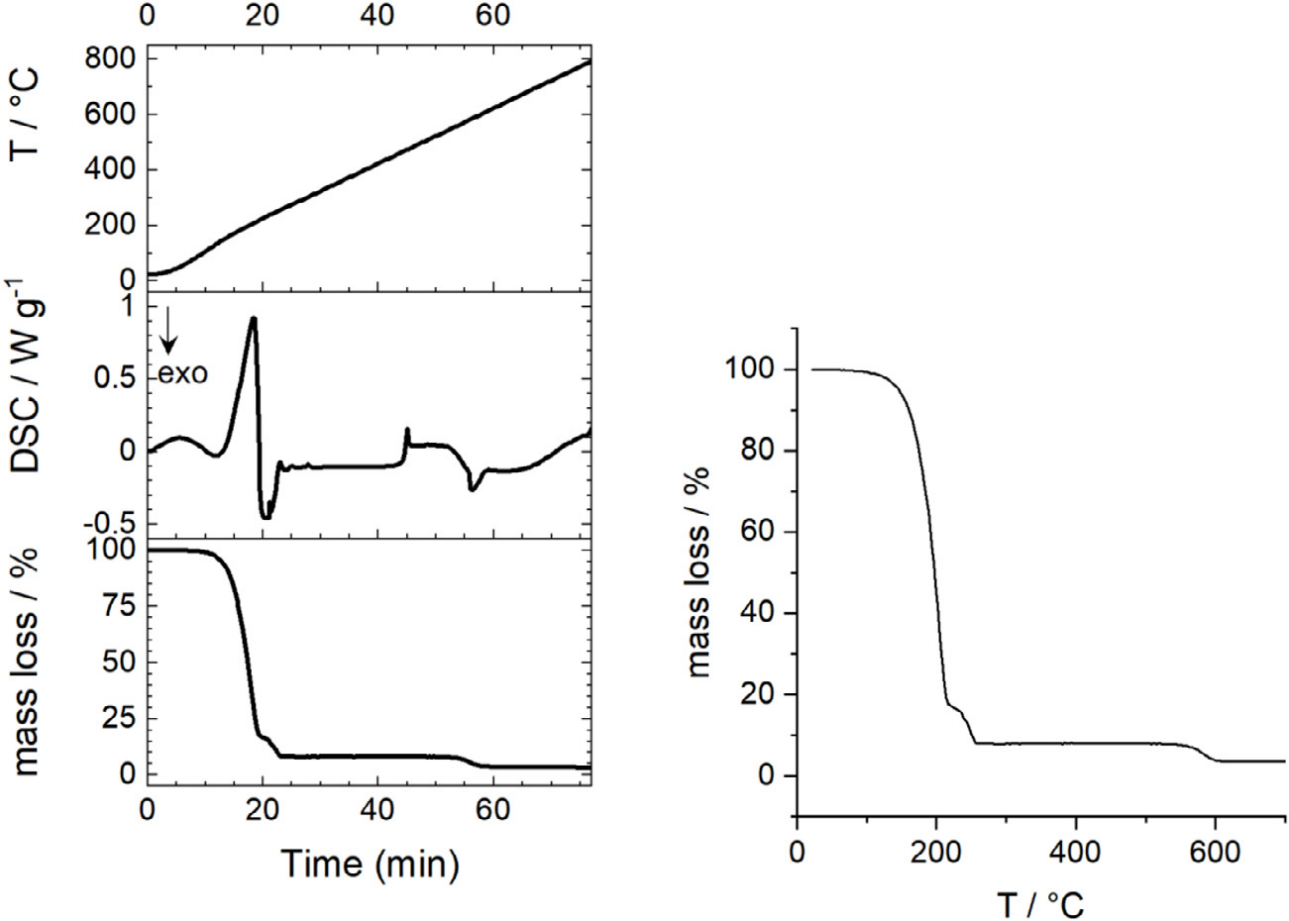
TGA analysis of the electrolyte mixture PC+EC+1 M NaClO_4_ with 10 K∙min^−1^ heating rate. Fig. SI-24a: Full range up to *T* = 750 °C. Fig. SI-24b: Mass loss vs. temperature plot for the electrolyte mixture.

In Fig. 26, a TGA-DSC analysis is depicted with the electrolyte mixture PC+EC+1M NaClO_4_. In addition to mass loss versus temperature, mass loss, DSC and temperature versus time are shown. Raw data are sown in Table 11 (supporting information).

## 2 Experimental Design, Materials and Methods

(a) Conductivity measurements

The electrochemical workstation "Zahner Zennium IM6″ was used to measure the EIS spectra of the electrolyte mixtures (frequency range: 50 kHz to 1 MHz). The measurements were performed in a RHD Instruments closed cell (TSC 1600 closed) filled with 850 µl of electrolyte in the glove box. The hermetically sealed cells were placed in a temperature chamber (Espec Oven SH 261) and waited for temperature constancy (waiting time about 20 min). The cell constant C of the RHD cell was determined by the measurement of KCl standard solution (1.413 mS cm^−1^ at 25 °C, Hanna instruments, HI 70,031). After measurement, conductivity values were determined from the internal resistance extracted from the phase minimum of the Bode plot (phase angle).

(b) Viscosity measurements

A Malvern Gemini HR Nano rotational rheometer was used for measuring the dynamic viscosity with a 40/1° cone geometry (sample filling gap: 30 μm). The mixtures were placed between cone and plate under normal atmosphere. A protective solvent evaporation hood was used during the measurement to avoid solvent evaporation. Measurements were carried out in a temperature range of 15–70 °C, and at each temperature a series of increased shear rates (5 to 200 s^−1^) was applied to ensure that viscosity was not dependent on shear rate.

(c) Density measurements

We measured the density values of the electrolyte mixtures using an Anton Paar DMA 4500 M instrument. Firstly, a check-up with air and water was performed to ensure a proper working of the device. Afterwards, the electrolytes were put into the device without any bubbles, and the measurement was performed (temperature range: 20 – 60 °C). Approximately 1.2 ml of solution was used for each measurement.

(d) Gas chromatography measurements

A Clarus 690 GC (PerkinElmer Inc., Waltham, USA) that was equipped with an autosampler, a FID (flame ionization detector), and an MS (mass spectrometry) detector (SQ 8T) was used for the measurement, while the software packages Turbomass 6.1.2 and TotalChrom 6.3.4 were used for both data acquisition and data analysis. The mixtures were diluted with MTBE and filled into GC vials. Measurements were then performed using an auto-sampler. Separation was performed with He gas 6.0, while the FID was operated with H2 gas (PG+160, Vici DBS) and dry air. Inside the GC oven, an Optima 5 MS column (30 m in length x 0.25 mm in inner diameter, 0.5 µm in film thickness) was used for separation. A split flow rate of 20 ml min^−1^ and an inlet temperature at 250 °C were used for injection. The injection volume equaled 0.5 µl. During the measurement, the pressure was continuously increased starting from 175 kPa (start pressure) with the following parameters:

- pressure-controlled mode
- oven temperature 40 °C
- oven and pressure parameters: 40 °C (1.5 min), heating at 20 °C min^−1^ (up to 320 °C)
- pressure from 175 kPa for 2 min, increasing at 7.8 kPa min^−1^ to 300 kPa.

The gas flow was divided after the separation column by a SilFlow™ GC Capillary Column 3-port splitter to capture signals in both the MS and FID. The MS setup consisted of a filament with a filament voltage that was 70 kV, an ion source temperature that was 200 °C, and an MS transfer line temperature that was 200 °C, respectively, while the FID setup featured 450 ml/min for synthetic air, 45 ml/min for hydrogen gas, and an FID temperature that was 280 °C. The FID was used for quantitative analysis, while the MS was used for identifying all compounds. Consequently, the MS was used in scan mode with a scan range of 33 u–350 u and an event time of 0.3 s. All signals from the FID were used to determine the peak area. First, the raw data were extracted using Turbomass 6.1.2 and then analyzed using OriginPro 2020b software. Impurities in the electrolyte solvents were analyzed based on NIST search (EI fragmentation match) and pure substance measurement wherever possible. Gas formation was tested in a PAT-Cell press (EL Cell), from which the spring in the upper housing was detached and a beaker containing electrolyte and sodium was placed in the lower housing. Following sealing, the pressure was monitored for 400 h at *T* = 25 °C. The gas was collected using a syringe fitted with a syringe stopper to prevent atmospheric gas from entering the syringe. The gas was then injected into a GC instrument (Arnel GC system from Perkin Elmer) and analyzed qualitatively using TotalChrom 6.3.4 software.

(e) *Cell testing*

The electrolytes were tested in coin cells to evaluate the performance and aging of the materials and electrolytes. Full cells of hard carbon versus Na_0.7_MnO_2_ (*Ø* = 16 mm) were assembled in type CR 2032 standard coin cells under protective atmosphere (Ar-filled glove box with humidity and oxygen content below 0.5 ppm). A glass fiber separator (*Ø* = 17 mm, QMA, Whatman®) that had been wetted with 110 µl of electrolyte mixture was placed between both electrodes. The electrodes as well as the separators were dried (vacuum, 110 °C, 24 h) before assembly. The theoretical surface area capacity of the electrode sheets equaled 2.2 mAh cm^−2^ (hard carbon) as well as 0.5 mAh cm^−2^ (sodium manganese oxide). This means that both electrodes were not balanced. Galvanostatic charge-discharge cycles were performed using the LICCY cell cycler (developed by KIT, Institute for Data Processing and Electronics). The cell tests were first performed with a series of continuously increased currents (C rate of charge/discharge as follows: 0.1C/0.1C, 0.2C/0.2C, 0.5C/0.5C, 0.5C/1C, 0.5C/1.5C, 0.5C/5C, 0.5C/7.5C, 0.5C/10C for 1 to 3 cycles), and then the cells were tested at a reduced rate (0.2C/0.2C for 2 cycles, 0.5C/1C for 100 cycles, and 0.2C/0.2C for 2 cycles). The applied C-rate was dependent on the capacity of the cathode materials used (lower capacity charge than HC).

(f) *Electrolyte simulation with AEM software*

The advanced electrolyte model (AEM) approach for calculating viscosity, density, conductivity, diffusion values, etc. had been published previously [7], [8], [9] and is available as a software tool. In principle, various physicochemical terms which were derived for multicomponent electrolytes were used to calculate these data. By using the INL's software package, all the values mentioned in the text were calculated, namely density, viscosity, conductivity and diffusion constants. The Advanced Electrolyte Model software can be licensed from the Idaho National Laboratory. Contact td@inl.gov for more information. In detail, appropriate solvents and salts were chosen in the software procedure and a range of concentration as well as temperature was applied. For Triple Ion stability, Option 1 was used that means [ABA^+^] = [BAB^−^]. Additionally, a contact angle of 0° and a total pore length of 0.1 µm are chosen. No surface-charge attenuated electrolyte permittivity calculation as well as double layer calculation was done. Finally, the desired values were extracted from the calculations.

(g) *Thermogravimetry analysis*

A STA 449 F3 from Netzsch was used for the thermogravimetric analysis of the "PC+EC+1 M NaClO_4_″ electrolyte mixture. 47.9 mg of the electrolyte was placed in an Al_2_O_3_ crucible (open) and measured between 25 °C and 700 °C under dry air atmosphere. Then, the temperature ramp was adjusted to 10 K min^−1^. To correct for the influences of the measurement system, blank runs or correction runs were performed under the same experimental conditions as for the samples. In addition, the DSC curve was recorded in parallel from *T* = 25 °C to *T* = 700 °C.

(h) *Hazard traffic light (HTL) and life cycle assessment (LCA)*

The hazard traffic light (HTL) qualitative method is a color-code of potential hazards for different substances first presented by Hofmann et al. [1]. It is based on the hazard statements described in the regulation of the European Parliament on classification, labeling and packaging [2] and as registered by the European Chemicals Agency – ECHA for each material. These statements can readily be extracted using the search engine for chemicals / regulated substances found in the ECHA homepage (https://echa.europa.eu/) and by typing in each one of the compounds herein presented. Specific details such as the European Community number and Infocard of each substance can be found in Table 12. A total number of 62 hazard statements grouped in 28 hazard classes are defined, each with a code, pictogram and signal words such as ‘danger´, ‘warning’ or no hazard word. An additional distinction between physical, health and environmental hazard is also taken into account. It is often the task of the producers and suppliers to classify their products following the previous guidelines, but for some specific substances a harmonization is done at the EU level when the perceived hazards are of major concern. Ultimately, a color is assigned to each hazard statement based on the respective signal word that it has received, which allows for a visual distinction of the potential hazardousness of a material. Red color will be assigned to hazards labelled as ‘danger´, whereas yellow will be used for those presented as ‘warning´. Statements without a hazard word are coloured in gray.

The life cycle assessment (LCA) method involves an assessment of the various environmental impacts of a product at different stages of its life cycle, i.e., raw material procurement, manufacturing, use, and final disposal. It closely follows the guidelines described in ISO standards 14,040/14,044. In this manuscript, a cradle-to-gate approach was used, which means that impacts are estimated only up to the final stage of electrolyte manufacture. A functional equivalent quantity of 1 L of electrolyte mixture was chosen and the analysis was carried out using the ReCiPe Midpoint 2016 impact assessment procedure, describing a set of 18 impact classes, of which each has a specific unit of reference. A calculation of the cumulative energy demand can be found in Table 9. The cumulative energy demand depicts the total energy consumed from non-renewable resources up to the final step of mixture production. Data for the preparation of the precursors were taken from both published patents and literature sources, as well as from the commercial life cycle inventory database Ecoinvent 3.7.1. OpenLCA v1.10 software [2], [3], [4], [5], [6] was used to perform the assessment.

## Ethics Statements

No ethics conflicts.

## CRediT authorship contribution statement

**Andreas Hofmann:** Conceptualization, Data curation, Investigation, Project administration, Supervision, Visualization, Validation, Writing – original draft, Writing – review & editing. **Zhengqi Wang:** Investigation, Visualization, Writing – review & editing. **Sebastian Pinto Bautista:** Investigation, Visualization, Writing – review & editing. **Marcel Weil:** Project administration, Supervision, Writing – review & editing. **Freya Müller:** Investigation, Writing – review & editing. **Robert Löwe:** Investigation, Writing – review & editing. **Luca Schneider:** Resources, Writing – review & editing. **Ijaz Ul Mohsin:** Resources, Writing – review & editing. **Thomas Hanemann:** Funding acquisition, Project administration, Resources, Supervision, Writing – review & editing.

## Declaration of Competing Interest

The authors declare that they have no known competing financial interests or personal relationships that could have appeared to influence the work reported in this paper.

## Appendix Supplementary materials

Image, application 1

Image, application 2
