# Supplementary material for: Dataset of propylene carbonate based liquid electrolyte mixtures for sodium-ion cells
Source: Data Brief. 2021 Dec 30;40:107775. doi: 10.1016/j.dib.2021.107775 (PMC8741479; doi:10.1016/j.dib.2021.107775)
Supplement: Supplementary file 1 [file mmc1.docx]

**Supporting Information, Tables**

**Title**

Dataset of propylene carbonate based liquid electrolyte mixtures for sodium-ion cells

**Authors**

Andreas Hofmann,^a,*^ Zhengqi Wang,^a,b^ Sebastian Pinto Bautista,^c,d^ Marcel Weil,^c^ Freya Müller,^a^ Robert Löwe,^a^ Luca Schneider,^a^ Ijaz Ul Mohsin^a^ and Thomas Hanemann^a,b^

**Affiliations**

a) Karlsruher Institut für Technologie, Institut für Angewandte Materialien (IAM), Herrmann-von-Helmholtz Platz 1, 76344 Eggenstein-Leopoldshafen, Germany

b) Department of Microsystems Engineering, University of Freiburg, Georges-Köhler-Allee 102, D-79110 Freiburg, Germany

c) Karlsruher Institut für Technologie, Institut für Technikfolgenabschätzung und Systemanalyse (ITAS), Postfach 3640, 76021 Karlsruhe, Germany

d) Helmholtz-Institut Ulm für Elektrochemische Energiespeicherung (HIU), Helmholtzstraße 11, 89081 Ulm, Germany

Table 1a. AEM calculation of the electrolyte mixtures PC+1 M NaClO_4_

|  | PC + 1 M NaClO_4_ | | | |
| --- | --- | --- | --- | --- |
| *T* | *d* | *D*_Na_ | *κ* | *η* |
| °C | g∙cm^-3^ | cm^2^∙s^-1^ | mS∙cm^-1^ | mPa∙s |
| 0 | 1.2933 | 2.08E-7 | 2.88 | 15.12 |
| 5 | 1.2885 | 2.88E-7 | 3.44 | 12.66 |
| 10 | 1.2829 | 3.91E-7 | 4.05 | 10.75 |
| 15 | 1.2782 | 5.18E-7 | 4.71 | 9.24 |
| 20 | 1.2731 | 6.77E-7 | 5.41 | 8.02 |
| 25 | 1.2679 | 8.73E-7 | 6.17 | 7.02 |
| 30 | 1.2631 | 1.11E-6 | 6.99 | 6.18 |
| 35 | 1.2574 | 1.41E-6 | 7.88 | 5.47 |
| 40 | 1.2528 | 1.76E-6 | 8.82 | 4.88 |
| 45 | 1.2481 | 2.18E-6 | 9.82 | 4.37 |
| 50 | 1.2426 | 2.67E-6 | 10.86 | 3.94 |
| 55 | 1.2377 | 3.23E-6 | 11.93 | 3.58 |
| 60 | 1.2331 | 3.85E-6 | 12.99 | 3.28 |
| 65 | 1.2274 | 4.49E-6 | 14.00 | 3.04 |
| 70 | 1.2227 | 5.12E-6 | 14.90 | 2.85 |
| 75 | 1.2180 | 5.69E-6 | 15.63 | 2.72 |
| 80 | 1.2130 | 6.10E-6 | 16.12 | 2.64 |
| 85 | 1.2083 | 6.29E-6 | 16.28 | 2.61 |
| 90 | 1.2030 | 6.18E-6 | 16.05 | 2.66 |

Table 1b. AEM calculation of the electrolyte mixtures PC+DMC+1 M NaClO_4_

|  | PC + DMC + 1 M NaClO_4_ | | | |
| --- | --- | --- | --- | --- |
| *T* | *d* | *D*_Na_ | *κ* | *η* |
| °C | g∙cm^-3^ | cm^2^∙s^-1^ | mS∙cm^-1^ | mPa∙s |
| 0 | 1.2284 | 5.75E-7 | 6.01 | 6.16 |
| 5 | 1.2235 | 7.25E-7 | 6.77 | 5.45 |
| 10 | 1.2180 | 9.03E-7 | 7.58 | 4.86 |
| 15 | 1.2130 | 1.11E-6 | 8.43 | 4.36 |
| 20 | 1.2077 | 1.36E-6 | 9.33 | 3.93 |
| 25 | 1.2026 | 1.64E-6 | 10.28 | 3.56 |
| 30 | 1.1974 | 1.98E-6 | 11.29 | 3.24 |
| 35 | 1.1927 | 2.37E-6 | 12.36 | 2.95 |
| 40 | 1.1874 | 2.82E-6 | 13.48 | 2.70 |
| 45 | 1.1825 | 3.34E-6 | 14.66 | 2.47 |
| 50 | 1.1769 | 3.93E-6 | 15.89 | 2.29 |
| 55 | 1.1722 | 4.59E-6 | 17.15 | 2.12 |
| 60 | 1.1674 | 5.30E-6 | 18.41 | 1.96 |
| 65 | 1.1620 | 6.06E-6 | 19.66 | 1.84 |
| 70 | 1.1570 | 6.84E-6 | 20.85 | 1.74 |
| 75 | 1.1517 | 7.42E-6 | 21.39 | 1.68 |
| 80 | 1.1471 | 8.10E-6 | 22.31 | 1.61 |
| 85 | 1.1418 | 8.67E-6 | 23.01 | 1.56 |
| 90 | 1.1369 | 9.05E-6 | 23.45 | 1.53 |

Table 1c. AEM calculation of the electrolyte mixtures PC+DEC+1 M NaClO_4_

|  | PC + DEC + 1 M NaClO_4_ | | | |
| --- | --- | --- | --- | --- |
| *T* | *d* | *D*_Na_ | *κ* | *η* |
| °C | g∙cm^-3^ | cm^2^∙s^-1^ | mS∙cm^-1^ | mPa∙s |
| 0 | 1.1638 | 4.66E-7 | 4.51 | 7.42 |
| 5 | 1.1589 | 5.90E-7 | 5.10 | 6.52 |
| 10 | 1.1536 | 7.39E-7 | 5.72 | 5.78 |
| 15 | 1.1485 | 9.15E-7 | 6.38 | 5.16 |
| 20 | 1.1434 | 1.12E-6 | 7.08 | 4.63 |
| 25 | 1.1381 | 1.37E-6 | 7.81 | 4.18 |
| 30 | 1.1332 | 1.65E-6 | 8.59 | 3.78 |
| 35 | 1.1277 | 1.99E-6 | 9.42 | 3.44 |
| 40 | 1.1230 | 2.37E-6 | 10.28 | 3.13 |
| 45 | 1.1174 | 2.82E-6 | 11.19 | 2.87 |
| 50 | 1.1126 | 3.33E-6 | 12.13 | 2.64 |
| 55 | 1.1072 | 3.89E-6 | 13.10 | 2.43 |
| 60 | 1.1022 | 4.52E-6 | 14.08 | 2.26 |
| 65 | 1.0971 | 5.18E-6 | 15.04 | 2.11 |
| 70 | 1.0919 | 5.86E-6 | 15.96 | 1.98 |
| 75 | 1.0870 | 6.54E-6 | 16.81 | 1.88 |
| 80 | 1.0815 | 7.16E-6 | 17.54 | 1.79 |
| 85 | 1.0768 | 7.68E-6 | 18.10 | 1.74 |
| 90 | 1.0713 | 8.04E-6 | 18.45 | 1.70 |

Table 1d. AEM calculation of the electrolyte mixtures PC+EMC+1 M NaClO_4_

|  | PC + EMC + 1 M NaClO_4_ | | | |
| --- | --- | --- | --- | --- |
| *T* | *d* | *D*_Na_ | *κ* | *η* |
| °C | g∙cm^-3^ | cm^2^∙s^-1^ | mS∙cm^-1^ | mPa∙s |
| 0 | 1.1927 | 6.41E-7 | 6.21 | 5.64 |
| 5 | 1.1874 | 7.98E-7 | 6.94 | 5.03 |
| 10 | 1.1825 | 9.83E-7 | 7.70 | 4.52 |
| 15 | 1.1769 | 1.20E-6 | 8.49 | 4.08 |
| 20 | 1.1722 | 1.45E-6 | 9.33 | 3.70 |
| 25 | 1.1666 | 1.74E-6 | 10.21 | 3.37 |
| 30 | 1.1620 | 2.08E-6 | 11.14 | 3.08 |
| 35 | 1.1564 | 2.47E-6 | 12.11 | 2.82 |
| 40 | 1.1517 | 2.92E-6 | 13.13 | 2.60 |
| 45 | 1.1461 | 3.44E-6 | 14.19 | 2.39 |
| 50 | 1.1415 | 4.01E-6 | 15.29 | 2.21 |
| 55 | 1.1362 | 4.64E-6 | 16.41 | 2.06 |
| 60 | 1.1313 | 5.33E-6 | 17.54 | 1.93 |
| 65 | 1.1258 | 6.05E-6 | 18.64 | 1.81 |
| 70 | 1.1211 | 6.81E-6 | 19.68 | 1.71 |
| 75 | 1.1155 | 7.52E-6 | 20.62 | 1.63 |
| 80 | 1.1107 | 8.17E-6 | 21.42 | 1.57 |
| 85 | 1.1054 | 8.69E-6 | 22.01 | 1.52 |
| 90 | 1.1008 | 9.03E-6 | 22.35 | 1.50 |

Table 1e. AEM calculation of the electrolyte mixtures PC+EC+1 M NaClO_4_

|  | PC + EC + 1 M NaClO_4_ | | | |
| --- | --- | --- | --- | --- |
| *T* | *d* | *D*_Na_ | *κ* | *η* |
| °C | g∙cm^-3^ | cm^2^∙s^-1^ | mS∙cm^-1^ | mPa∙s |
| 0 | 1.3502 | 2.87E-7 | 3.98 | 11.41 |
| 5 | 1.3454 | 3.92E-7 | 4.69 | 9.66 |
| 10 | 1.3397 | 5.24E-7 | 5.45 | 8.29 |
| 15 | 1.3350 | 6.87E-7 | 6.26 | 7.19 |
| 20 | 1.3297 | 8.85E-7 | 7.13 | 6.31 |
| 25 | 1.3245 | 1.13E-6 | 8.05 | 5.57 |
| 30 | 1.3198 | 1.41E-6 | 9.02 | 4.96 |
| 35 | 1.3143 | 1.76E-6 | 10.05 | 4.43 |
| 40 | 1.3094 | 2.17E-6 | 11.12 | 4.00 |
| 45 | 1.3047 | 2.63E-6 | 12.25 | 3.63 |
| 50 | 1.2990 | 3.16E-6 | 13.40 | 3.31 |
| 55 | 1.2943 | 3.77E-6 | 14.58 | 3.03 |
| 60 | 1.2894 | 4.43E-6 | 15.76 | 2.80 |
| 65 | 1.2839 | 5.14E-6 | 16.91 | 2.60 |
| 70 | 1.2792 | 5.87E-6 | 18.00 | 2.45 |
| 75 | 1.2745 | 6.60E-6 | 18.99 | 2.31 |
| 80 | 1.2693 | 7.25E-6 | 19.83 | 2.21 |
| 85 | 1.2641 | 7.81E-6 | 20.47 | 2.14 |
| 90 | 1.2594 | 8.18E-6 | 20.84 | 2.11 |

Table 1f. AEM calculation of the electrolyte mixtures PC+G1+1 M NaClO_4_

|  | PC + G1 + 1 M NaClO_4_ | | | |
| --- | --- | --- | --- | --- |
| *T* | *d* | *D*_Na_ | *κ* | *η* |
| °C | g∙cm^-3^ | cm^2^∙s^-1^ | mS∙cm^-1^ | mPa∙s |
| 0 | 1.1164 | 1.15E-6 | 11.70 | 3.34 |
| 5 | 1.1107 | 1.40E-6 | 12.84 | 3.04 |
| 10 | 1.1054 | 1.68E-6 | 14.01 | 2.78 |
| 15 | 1.1004 | 2.00E-6 | 15.23 | 2.55 |
| 20 | 1.0947 | 2.37E-6 | 16.51 | 2.34 |
| 25 | 1.0897 | 2.79E-6 | 17.85 | 2.16 |
| 30 | 1.0842 | 3.26E-6 | 19.25 | 2.00 |
| 35 | 1.0787 | 3.81E-6 | 20.71 | 1.85 |
| 40 | 1.0741 | 4.44E-6 | 22.23 | 1.72 |
| 45 | 1.0685 | 5.13E-6 | 23.81 | 1.61 |
| 50 | 1.0631 | 5.91E-6 | 25.43 | 1.50 |
| 55 | 1.0575 | 6.75E-6 | 27.07 | 1.41 |
| 60 | 1.0520 | 7.66E-6 | 28.69 | 1.33 |
| 65 | 1.0469 | 8.61E-6 | 30.27 | 1.26 |
| 70 | 1.0413 | 9.56E-6 | 31.75 | 1.19 |
| 75 | 1.0357 | 1.04E-5 | 33.05 | 1.15 |
| 80 | 1.0311 | 1.13E-5 | 34.13 | 1.11 |
| 85 | 1.0255 | 1.19E-5 | 34.88 | 1.08 |
| 90 | 1.0199 | 1.23E-5 | 35.22 | 1.08 |

Table 1g. AEM calculation of the electrolyte mixtures PC+G2+1 M NaClO_4_

|  | PC + G2 + 1 M NaClO_4_ | | | |
| --- | --- | --- | --- | --- |
| *T* | *d* | *D*_Na_ | *κ* | *η* |
| °C | g∙cm^-3^ | cm^2^∙s^-1^ | mS∙cm^-1^ | mPa∙s |
| 0 | 1.1369 | 4.15E-7 | 4.74 | 8.29 |
| 5 | 1.1323 | 5.33E-7 | 5.41 | 7.24 |
| 10 | 1.1267 | 6.74E-7 | 6.12 | 6.37 |
| 15 | 1.1211 | 8.44E-7 | 6.87 | 5.65 |
| 20 | 1.1164 | 1.05E-6 | 7.68 | 5.03 |
| 25 | 1.1109 | 1.30E-6 | 8.53 | 4.51 |
| 30 | 1.1060 | 1.58E-6 | 9.45 | 4.05 |
| 35 | 1.1008 | 1.92E-6 | 10.42 | 3.67 |
| 40 | 1.0957 | 2.32E-6 | 11.45 | 3.32 |
| 45 | 1.0906 | 2.79E-6 | 12.54 | 3.02 |
| 50 | 1.0853 | 3.33E-6 | 13.68 | 2.76 |
| 55 | 1.0806 | 3.94E-6 | 14.85 | 2.53 |
| 60 | 1.0750 | 4.62E-6 | 16.05 | 2.34 |
| 65 | 1.0703 | 5.35E-6 | 17.24 | 2.17 |
| 70 | 1.0649 | 6.13E-6 | 18.39 | 2.03 |
| 75 | 1.0593 | 6.91E-6 | 19.46 | 1.91 |
| 80 | 1.0544 | 7.64E-6 | 20.40 | 1.82 |
| 85 | 1.0493 | 8.29E-6 | 21.15 | 1.75 |
| 90 | 1.0441 | 8.76E-6 | 21.67 | 1.71 |

Table 1h. AEM calculation of the electrolyte mixtures PC+G4+1 M NaClO_4_

|  | PC + G4 + 1 M NaClO_4_ | | | |
| --- | --- | --- | --- | --- |
| *T* | *d* | *D*_Na_ | *κ* | *η* |
| °C | g∙cm^-3^ | cm^2^∙s^-1^ | mS∙cm^-1^ | mPa∙s |
| 0 | 1.1582 | 1.32E-7 | 1.55 | 26.74 |
| 5 | 1.1532 | 1.81E-7 | 1.88 | 21.98 |
| 10 | 1.1480 | 2.44E-7 | 2.24 | 18.35 |
| 15 | 1.1428 | 3.23E-7 | 2.63 | 15.51 |
| 20 | 1.1378 | 4.20E-7 | 3.05 | 13.25 |
| 25 | 1.1324 | 5.40E-7 | 3.51 | 11.44 |
| 30 | 1.1276 | 6.87E-7 | 4.01 | 9.95 |
| 35 | 1.1220 | 8.65E-7 | 4.54 | 8.71 |
| 40 | 1.1173 | 1.08E-6 | 5.11 | 7.68 |
| 45 | 1.1119 | 1.34E-6 | 5.71 | 6.81 |
| 50 | 1.1070 | 1.63E-6 | 6.34 | 6.08 |
| 55 | 1.1017 | 1.98E-6 | 6.99 | 5.47 |
| 60 | 1.0966 | 2.38E-6 | 7.65 | 4.95 |
| 65 | 1.0915 | 2.81E-6 | 8.31 | 4.51 |
| 70 | 1.0862 | 3.27E-6 | 8.95 | 4.16 |
| 75 | 1.0814 | 3.75E-6 | 9.55 | 3.87 |
| 80 | 1.0759 | 4.21E-6 | 10.08 | 3.65 |
| 85 | 1.0713 | 4.63E-6 | 10.50 | 3.47 |
| 90 | 1.0658 | 4.94E-6 | 10.79 | 3.36 |

Table 1i. AEM calculation of the electrolyte mixtures PC+SL+1 M NaClO_4_

|  | PC + SL + 1 M NaClO_4_ | | | |
| --- | --- | --- | --- | --- |
| *T* | *d* | *D*_Na_ | *κ* | *η* |
| °C | g∙cm^-3^ | cm^2^∙s^-1^ | mS∙cm^-1^ | mPa∙s |
| 0 | 1.3255 | 1.44E-7 | 1.88 | 24.82 |
| 5 | 1.3208 | 1.96E-7 | 2.24 | 20.80 |
| 10 | 1.3153 | 2.63E-7 | 2.63 | 17.68 |
| 15 | 1.3104 | 3.46E-7 | 3.06 | 15.19 |
| 20 | 1.3056 | 4.49E-7 | 3.53 | 13.17 |
| 25 | 1.2999 | 5.76E-7 | 4.03 | 11.51 |
| 30 | 1.2952 | 7.31E-7 | 4.57 | 10.13 |
| 35 | 1.2904 | 9.19E-7 | 5.15 | 8.97 |
| 40 | 1.2848 | 1.15E-6 | 5.77 | 8.00 |
| 45 | 1.2801 | 1.42E-6 | 6.43 | 7.16 |
| 50 | 1.2745 | 1.73E-6 | 7.12 | 6.45 |
| 55 | 1.2697 | 2.10E-6 | 7.84 | 5.85 |
| 60 | 1.2650 | 2.51E-6 | 8.58 | 5.33 |
| 65 | 1.2594 | 2.97E-6 | 9.33 | 4.90 |
| 70 | 1.2547 | 3.46E-6 | 10.05 | 4.54 |
| 75 | 1.2500 | 3.97E-6 | 10.74 | 4.24 |
| 80 | 1.2445 | 4.46E-6 | 11.35 | 4.01 |
| 85 | 1.2396 | 4.91E-6 | 11.86 | 3.84 |
| 90 | 1.2349 | 5.25E-6 | 12.22 | 3.72 |

Table 2a. Conductivity as well as density values at T = 25 °C for all mixtures with 1M sodium perchlorate. Additionally, the sodium diffusion coefficient from the AEM software calculation is provided.

| Mixture | *d* /  g∙cm^-3^ | *d* /  g∙cm^-3^ | *κ* /  mS∙cm^-1^ | *κ* /  mS∙cm^-1^ | *D*_Na_ /  10^-7^ cm^2^∙s^-1^ |
| --- | --- | --- | --- | --- | --- |
|  | experimental | AEM | experimental | AEM | AEM |
| PC | 1.2616 | 1.27 | 5.82 | 6.17 | 8.73 |
| PC+DMC | 1.2105 | 1.20 | 7.41 | 10.28 | 16.43 |
| PC+DEC | 1.1439 | 1.14 | 4.26 | 7.81 | 13.66 |
| PC+EMC | 1.1741 | 1.17 | 5.57 | 10.21 | 17.41 |
| PC+DPrC | 1.1726 | --- | 3.63 | --- | --- |
| PC+EC | 1.3243 | 1.32 | 7.08 | 8.05 | 11.28 |
| PC+12BC | 1.2353 | --- | 4.18 | --- | --- |
| PC+G1 | 1.1082 | 1.09 | 10.00 | 17.85 | 27.86 |
| PC+G2 | 1.1241 | 1.11 | 7.85 | 8.53 | 12.96 |
| PC+G4 | 1.1363 | 1.13 | 3.21 | 3.51 | 5.40 |
| PC+SL | 1.2984 | 1.30 | 3.22 | 4.03 | 5.76 |

Table 2b. Viscosity values at T = 25 °C for all mixtures with 1M sodium perchlorate including the flow activation energy according to Arrhenius fitting.

| Mixture | *η* /  mPa∙s | *η* /  mPa∙s | Arrhenius flow activation energy E_A_ / kJ∙mol^-1^ | R^2^ for the linear fit |
| --- | --- | --- | --- | --- |
|  | experimental | AEM | based on experimental rheology data^[a]^ |  |
| PC | 7.27 | 7.02 | 19.8 | 0.9993 |
| PC+DMC | 4.08 | 3.56 | 13.0 | 0.9987 |
| PC+DEC | 4.08 | 4.18 | 16.6 | 0.9990 |
| PC+EMC | 3.85 | 3.37 | 10.6 | 0.9980 |
| PC+DPrC | 6.43 | --- | 19.0 | 0.9979 |
| PC+EC | 6.84 | 5.57 | 19.0 | 0.9995 |
| PC+12BC | 8.32 | --- | 21.5 | 0.9984 |
| PC+G1 | 3.41 | 2.16 | 11.1 | 0.9976 |
| PC+G2 | 4.33 | 4.51 | 16.3 | 0.9977 |
| PC+G4 | 10.90 | 11.44 | 20.8 | 0.9988 |
| PC+SL | 14.72 | 11.51 | 23.1 | 0.9981 |

[a] temperature range for PC+DEC and PC+G1 was 15 °C - 45 °C, because at higher temperatures there was a significant deviation from the linearity, otherwise the full temperature range was used for the fitting (15 °C - 60 °C).

Table 3a. Experimental conductivity values for 1M NaClO_4_ containing electrolytes (*κ* in S∙cm^-1^).

|  |  | PC | PC+DMC | PC+DEC | PC+EMC | PC+DPrC | PC+EC | PC+12BC | PC+G1 | PC+G2 | PC+G4 | PC+SL |
| --- | --- | --- | --- | --- | --- | --- | --- | --- | --- | --- | --- | --- |
| 20 |  | 5.3 | 6.6 | 3.9 | 5.0 | 3.2 | 6.3 | 3.7 | 9.3 | 7.2 | 2.8 | 2.8 |
| 30 |  | 6.5 | 8.1 | 4.6 | 6.1 | 4.0 | 7.9 | 4.8 | 10.7 | 8.6 | 3.7 | 3.7 |
| 40 |  | 7.9 | 10.3 | 5.4 | 7.3 | 4.9 | 9.6 | 5.9 | 12.2 | 10.0 | 4.8 | 4.6 |
| 50 |  | 9.4 | 12.9 | 6.3 | 8.6 | 5.8 | 11.3 | 7.2 | 13.7 | 11.4 | 5.9 | 5.7 |

Table 3b. Experimental viscosity values for 1M NaClO_4_ containing electrolytes (*η* in mPa∙s).

|  | PC | PC+DMC | PC+DEC | PC+EMC | PC+DPrC | PC+EC | PC+12BC | PC+G1 | PC+G2 | PC+G4 | PC+SL |
| --- | --- | --- | --- | --- | --- | --- | --- | --- | --- | --- | --- |
| 20 | 8.4 | 4.5 | 4.6 | 4.2 | 7.5 | 7.8 | 9.8 | 3.7 | 4.9 | 12.5 | 17.5 |
| 30 | 6.3 | 3.7 | 3.7 | 3.6 | 5.7 | 6.0 | 7.1 | 3.2 | 3.9 | 9.5 | 12.5 |
| 40 | 4.9 | 3.2 | 3.1 | 3.1 | 4.5 | 4.7 | 5.4 | 2.8 | 3.1 | 7.4 | 9.4 |
| 50 | 3.9 | 2.7 | 2.7 | 2.8 | 3.6 | 3.8 | 4.3 | 2.6 | 2.6 | 5.6 | 7.2 |

Table 3c. Experimental density values for 1M NaClO_4_ containing electrolytes (*d* in g∙cm^-3^).

|  | PC | PC+DMC | PC+DEC | PC+EMC | PC+DPrC | PC+EC | PC+12BC | PC+G1 | PC+G2 | PC+G4 | PC+SL |
| --- | --- | --- | --- | --- | --- | --- | --- | --- | --- | --- | --- |
| 20 | 1.2626 | 1.2160 | 1.1492 | 1.1795 | 1.1776 | 1.3297 | 1.2403 | 1.1133 | 1.1289 | 1.1413 | 1.3033 |
| 30 | 1.2529 | 1.2045 | 1.1387 | 1.1687 | 1.1675 | 1.3189 | 1.2303 | 1.1032 | 1.1194 | 1.1315 | 1.2935 |
| 40 | 1.2422 | 1.1936 | 1.1282 | 1.1579 | 1.1574 | 1.3082 | 1.2202 | 1.0930 | 1.1098 | 1.1221 | 1.2839 |
| 50 | 1.2315 | 1.1824 | 1.1176 | 1.1470 | 1.1473 | 1.2974 | 1.2103 | 1.0828 | 1.1002 | 1.1129 | 1.2744 |

Table 4. Comparison of the experimental values at T = 25 °C and T = 50 °C.

| Mixture | *κ (T = 50 °C) / κ (T = 25 °C)* | *η (T = 50 °C) / η (T = 25 °C)* |
| --- | --- | --- |
|  | experimental | experimental |
| PC | 1.62 | 0.54 |
| PC+DMC | 1.74 | 0.66 |
| PC+DEC | 1.47 | 0.67 |
| PC+EMC | 1.54 | 0.73 |
| PC+DPrC | 1.60 | 0.56 |
| PC+EC | 1.60 | 0.56 |
| PC+12BC | 1.73 | 0.51 |
| PC+G1 | 1.37 | 0.75 |
| PC+G2 | 1.46 | 0.60 |
| PC+G4 | 1.83 | 0.52 |
| PC+SL | 1.77 | 0.49 |

Table 5a. Relative FID area to solvent (PC) for individual components, electrolyte without NaClO_4_

| PC | PC+DMC | PC+DEC | PC+EMC | PC+DPrC | PC+EC | PC+12BC | PC+G1 | PC+G2 | PC+G4 | PC+SL |
| --- | --- | --- | --- | --- | --- | --- | --- | --- | --- | --- |
| 5.838E-5 | 6.269E-4 | 1.559E-4 | 3.102E-4 | 1.472E-4 |  | 4.172E-4 | 5.405E-5 | 0.004 | 4.65E-4 | 5.379E-5 |
| 2.193E-4 | 2.819E-4 | 9.172E-5 | 0.06 | 2.999E-5 |  | 2.859E-4 | 0.001 | 0.002 | 2.859E-4 | 5.64E-5 |
| 1.505E-4 | 3.046E-4 | 5.851E-5 | 0.213 | 4.076E-5 |  | 9.665E-5 | 4.49E-5 | 0.002 | 5.549E-5 |  |
|  | 8.757E-5 | 0.006 | 9.985E-5 | 2.51E-5 |  | 3.292E-5 | 6.165E-5 | 1.901E-4 | 3.457E-5 |  |
|  | 0.006 | 2.555E-4 | 6.61E-5 | 0.003 |  | 4.2E-5 | 3.068E-5 | 3.173E-5 | 0.002 |  |
|  | 4.393E-4 |  | 8.709E-5 | 5.335E-4 |  |  | 3.874E-5 | 0.002 | 7.315E-5 |  |
|  |  |  | 0.001 |  |  |  | 0.003 | 0.005 | 7.977E-5 |  |
|  |  |  | 0.004 |  |  |  | 1.384E-4 | 4.091E-4 | 5.651E-5 |  |
|  |  |  | 0.002 |  |  |  | 1.948E-4 | 0.003 | 4.366E-4 |  |
|  |  |  | 9.071E-5 |  |  |  | 1.068E-4 | 1.225E-4 | 3.675E-5 |  |
|  |  |  | 2.185E-4 |  |  |  | 1.814E-4 | 2.032E-4 | 5.668E-5 |  |
|  |  |  | 1.236E-4 |  |  |  | 1.735E-4 | 5.013E-5 | 0.001 |  |
|  |  |  |  |  |  |  | 0.002 | 9.668E-4 | 0.001 |  |
|  |  |  |  |  |  |  | 6E-4 | 7.303E-4 | 3.364E-4 |  |
|  |  |  |  |  |  |  | 4.066E-4 | 6.167E-4 | 8.603E-5 |  |
|  |  |  |  |  |  |  | 6.78E-5 | 2.639E-4 | 1.745E-4 |  |
|  |  |  |  |  |  |  | 7.485E-5 |  | 5.861E-5 |  |
|  |  |  |  |  |  |  | 3.67E-5 |  | 5.04E-4 |  |
|  |  |  |  |  |  |  | 2.894E-4 |  | 2.407E-4 |  |
|  |  |  |  |  |  |  | 4.764E-4 |  | 0.001 |  |
|  |  |  |  |  |  |  | 2.576E-4 |  | 6.783E-5 |  |
|  |  |  |  |  |  |  | 2.969E-5 |  | 4.54E-5 |  |
|  |  |  |  |  |  |  | 1.844E-4 |  | 2.642E-4 |  |
|  |  |  |  |  |  |  | 4.605E-4 |  | 1.161E-4 |  |
|  |  |  |  |  |  |  | 3.541E-4 |  | 3.898E-4 |  |
|  |  |  |  |  |  |  | 2.267E-4 |  | 3.762E-4 |  |
|  |  |  |  |  |  |  | 5.103E-4 |  | 1.457E-4 |  |
|  |  |  |  |  |  |  | 2.138E-4 |  | 5.109E-5 |  |
|  |  |  |  |  |  |  | 3.159E-5 |  | 0.001 |  |
|  |  |  |  |  |  |  | 1.393E-4 |  | 1.679E-4 |  |
|  |  |  |  |  |  |  | 1.799E-4 |  | 1.99E-4 |  |
|  |  |  |  |  |  |  | 1.966E-4 |  | 8.806E-5 |  |
|  |  |  |  |  |  |  | 1.39E-4 |  | 2.074E-4 |  |
|  |  |  |  |  |  |  | 1.672E-4 |  | 9.09E-5 |  |
|  |  |  |  |  |  |  | 5.989E-5 |  | 5.183E-5 |  |
|  |  |  |  |  |  |  | 4.342E-5 |  |  |  |
|  |  |  |  |  |  |  | 2.92E-5 |  |  |  |
|  |  |  |  |  |  |  | 5.393E-5 |  |  |  |
|  |  |  |  |  |  |  | 6.693E-5 |  |  |  |
|  |  |  |  |  |  |  | 2.309E-4 |  |  |  |
|  |  |  |  |  |  |  | 1.797E-4 |  |  |  |
|  |  |  |  |  |  |  | 3.246E-4 |  |  |  |
|  |  |  |  |  |  |  | 1.027E-4 |  |  |  |
|  |  |  |  |  |  |  | 2.399E-4 |  |  |  |
|  |  |  |  |  |  |  | 2.481E-4 |  |  |  |
|  |  |  |  |  |  |  | 3.852E-4 |  |  |  |

Table 5b. Relative FID area to solvent (PC) for individual components, electrolyte with NaClO_4_

| PC | PC+DMC | PC+DEC | PC+EMC | PC+DPrC | PC+EC | PC+12BC | PC+G1 | PC+G2 | PC+G4 | PC+SL |
| --- | --- | --- | --- | --- | --- | --- | --- | --- | --- | --- |
| 0.004 | 0.013 | 0.002 | 0.001 | 0.007 | 2.6E-4 | 0.002 | 8.644E-4 | 5.833E-4 | 2.541E-4 | 3.018E-4 |
| 0.008 | 0.008 | 0.008 | 0.015 | 5.293E-5 | 0.004 | 0.003 | 0.002 | 7.617E-4 | 0.004 | 0.006 |
| 0.007 | 0.003 | 0.005 | 0.064 | 7.026E-4 | 9.686E-4 | 0.003 | 0.01 | 0.004 | 3.651E-4 | 4.44E-4 |
| 4.571E-4 | 3.088E-4 | 0.001 | 0.008 | 0.004 | 0.002 | 2.545E-4 | 8.798E-5 | 3.777E-4 | 1.142E-4 |  |
| 1.214E-4 | 0.004 | 0.001 | 3.359E-4 | 7.343E-4 | 4.609E-4 | 0.004 | 4.828E-4 | 3.289E-4 | 3.927E-5 |  |
| 3.03E-4 | 2.303E-4 | 1.411E-4 | 0.001 | 4.554E-4 | 2.526E-4 | 2.622E-4 | 5.056E-4 | 1.418E-4 | 1.079E-4 |  |
| 4.075E-4 | 0.004 | 9.695E-4 | 0.265 | 0.011 |  | 4.993E-4 | 5.35E-4 | 7.952E-5 |  |  |
| 2.989E-4 | 0.002 | 0.004 | 8.199E-4 | 1.178E-4 |  | 1.99E-4 | 2.134E-4 | 1.285E-4 |  |  |
|  |  | 0.001 | 3.325E-4 | 1.88E-4 |  | 2.152E-4 | 6.132E-4 | 1.675E-4 |  |  |
|  |  |  | 0.002 | 9.993E-5 |  | 1.19E-4 | 1.537E-4 | 6.438E-5 |  |  |
|  |  |  | 4.207E-4 | 0.002 |  | 4.672E-4 | 3.501E-4 |  |  |  |
|  |  |  | 0.002 | 4.966E-4 |  | 1.362E-4 | 2.427E-4 |  |  |  |
|  |  |  | 6.764E-4 | 1.852E-4 |  | 1.096E-4 | 1.614E-4 |  |  |  |
|  |  |  | 8.161E-4 | 7.74E-4 |  |  | 1.665E-4 |  |  |  |
|  |  |  | 0.003 | 8.388E-4 |  |  | 1.407E-4 |  |  |  |
|  |  |  | 0.002 | 7.044E-4 |  |  | 1.729E-4 |  |  |  |
|  |  |  | 5.105E-4 | 0.004 |  |  |  |  |  |  |
|  |  |  | 0.001 | 2.906E-4 |  |  |  |  |  |  |
|  |  |  | 8.904E-4 | 1.318E-4 |  |  |  |  |  |  |
|  |  |  |  | 0.004 |  |  |  |  |  |  |
|  |  |  |  | 3.377E-4 |  |  |  |  |  |  |
|  |  |  |  | 3.832E-4 |  |  |  |  |  |  |

Table 5c. Relative intensity of the CO_2_ peak after 4 month of storage related to fresh samples.

| Mixture | Electrolyte over Na with NaClO_4_ | Electrolyte over Na without NaClO_4_ |
| --- | --- | --- |
| PC | 0.104 | 0.0013 |
| PC+DMC | 0.086 | 0.0013 |
| PC+DEC | 0.065 | 0.0011 |
| PC+EMC | 0.071 | 0.0014 |
| PC+DPrC | 0.064 | 0.0013 |
| PC+EC | 0.088 | 0.0016 |
| PC+12BC | 0.074 | 0.0045 |
| PC+G1 | 0.058 | 0.0014 |
| PC+G2 | 0.045 | 0.0012 |
| PC+G4 | 0.046 | 0.0012 |
| PC+SL | 0.058 | 0.0014 |

Table 7. Results of selected impurities found in pure solvents with gas chromatography and retention times of pure n-alkanes.

| solvent^[a]^ | impurity (identified) | retention time (FID) | retention time (MS) | RI^[b]^ | RI from NIST^[c]^ database | FID area relative to solvent area^[d]^ | verification | | | | | | | |
| --- | --- | --- | --- | --- | --- | --- | --- | --- | --- | --- | --- | --- | --- | --- |
|  |  | [min, Peak maximum] | [min, onset] |  |  | [%] | NIST / (Match/ 1000) | Retention, pure compound ^[e]^ | EI fragmentation, pure compound^[f]^ | mass fragmentation *m*/*z* in descending intensity order | | | | |
| EMC | DMC | 2.89 | 2.99 | 615 | 620 | 5.7 | 907 | x | x | 45 | 59 | 90 | 62 | 60 |
| EMC | DEC | 4.54 | 4.64 | 781 | 767 | 19.0 | 924 | x | x | 45 | 91 | 63 | 43 | 59 |
| DPrC | di-*n*-propyl ether | 3.53 | 3.63 | 681 | 680 | 0.2 | 885 | --- | --- | 43 | 41 | 73 | 102 | 58 |
| DPrC | sec-butyl propyl carbonate | 6.82 | 6.93 | 1013 | (996) | 0.02 | 790 | --- | --- | 57 | 41 | 45 | 44 | 56 |
| 12-BC | 1,2-butanediol | 5.13 | 5.23 | 839 | (824) | 0.1 | 830 | x | x | 59 | 41 | 43 | 58 | 57 |
| 12-BC | propylene carbonate | 6.58 | 6.67 | 985 | 931 | 0.03 | 752 | x | x | 57 | 44 | 43 | 87 | 58 |
| 12-BC | ? | 6.68 | 6.78 | 997 | --- | 0.04 | --- | --- | --- | 101 | 44 | 43 | 57 | 41 |
| 12-BC | ? | 6.80 | 6.90 | 1010 | --- | 0.02 | --- | --- | --- | 43 | 44 | 45 | 41 | 57 |
| G1 | 1,4-dioxane | 3.78 | 3.88 | 707 | 695 | 0.03 | 857 | x | x | 88 | 58 | 43 | 57 | 87 |
| G1 | butylated hydroxytoluene | 10.66 | 10.77 | 1519 | 1513 | 0.02 | 724 | x | x | 205 | 57 | 41 | 145 | 220 |
| G2 | dimethoxyethane | 3.22 | 3.32 | 649 | 643 | 0.01 | 879 | x | x | 45 | 60 | 90 | 58 | 43 |
| G2 | 1,4-dioxane | 3.77 | 3.88 | 707 | 695 | 0.01 | 878 | x | x | 88 | 58 | 43 | 57 | 87 |
| G2 | ? | 6.77 | 6.86 | 1006 | --- | 0.03 | --- | --- | --- | 73 | 59 | 45 | 117 | 41 |
| G2 | ? | 6.89 | 7.00 | 1022 | --- | 0.02 | --- | --- | --- | 59 | 73 | 72 | 43 | 41 |
| G2 | butylated hydroxytoluene | 10.65 | 10.77 | 1519 | 1513 | 0.02 | 731 | x | x | 205 | 41 | 57 | 220 | 145 |
| G4 | ? | 6.80 | 6.92 | 1013 | --- | 0.01 | --- | --- | --- | 45 | 59 | 58 | 43 | 44 |
| G4 | Triethylene glycol monomethyl ether | 8.52 | 8.63 | 1217 | 1223 | 0.01 | 824 | x | x | 45 | 59 | 58 | 89 | 43 |
| G4 | Triglyme | 8.56 | 8.67 | 1222 | 1232 | 0.07 | 848 | --- | --- | 59 | 58 | 45 | 43 | 103 |
| G4 | ? | 9.06 | 9.17 | 1287 | --- | 0.03 | --- | --- | --- | 59 | 45 | 43 | 58 | 44 |
| G4 | Diethylene glycol di-n-butyl ether | 10.10 | 10.22 | 1437 | (1442) | 0.04 | 839 | x | x | 57 | 41 | 56 | 45 | 75 |
| G4 | ? | 10.92 | 11.03 | 1556 | --- | 0.05 | --- | --- | --- | 59 | 58 | 45 | 73 | 103 |
| G4 | ? | 11.20 | 11.30 | 1594 | --- | 0.04 | --- | --- | --- | 59 | 45 | 147 | 103 | 58 |
| G4 | ? | 12.71 | 12.82 | 1765 | --- | 0.1 | --- |  |  | 59 | 58 | 103 | 45 | 87 |
| SL | ? | 8.84 | 8.95 | 1258 | --- | 0.05 | --- | --- | --- | 89 | 39 | 53 | 55 | 120 |
| SL | ? | 10.91 | 11.03 | 1556 | --- | 0.01 | --- | --- | --- | 69 | 55 | 64 | 41 | 56 |
| SL | ? | 11.96 | 12.07 | 1689 | --- | 0.02 | --- | --- | --- | 91 | 79 | 64 | 77 | 106 |
| SL | ? | 12.09 | 12.21 | 1705 | --- | 0.01 | --- | --- | --- | 67 | 79 | 157 | 41 | 109 |
|  |  |  |  |  |  |  |  |  |  |  |  |  |  |  |
| MTBE | C4-alkene | 1.82 | 1.95 | --- | --- | --- | --- | --- | --- | 56 | 41 | 39 | 55 | 50 |
| MTBE | 2-Methoxy butane | 2.71 | 2.81 | 593 | (530) | --- | 931 | --- | --- | 59 | 73 | 41 | 45 | 43 |
| MTBE | *tert*-butyl formate | 5.37 | 5.47 | 862 |  | --- | 837 | --- | --- | 57 | 41 | 59 | 56 | 87 |
|  |  |  |  |  |  |  |  |  |  |  |  |  |  |  |
| n-C4 | --- | 1.89 | 1.97 | --- |  | --- | --- | --- | --- | --- | --- | --- | --- | --- |
| n-C5 | --- | 2.17 | 2.24 | --- |  | --- | --- | --- | --- | --- | --- | --- | --- | --- |
| n-C6 | --- | 2.75 | 2.85 | --- |  | --- | --- | --- | --- | --- | --- | --- | --- | --- |
| n-C7 | --- | 3.71 | 3.81 | --- |  | --- | --- | --- | --- | --- | --- | --- | --- | --- |
| n-C8 | --- | 4.74 | 4.83 | --- |  | --- | --- | --- | --- | --- | --- | --- | --- | --- |
| n-C9 | --- | 5.76 | 5.86 | --- |  | --- | --- | --- | --- | --- | --- | --- | --- | --- |
| n-C10 | --- | 6.70 | 6.81 | --- |  | --- | --- | --- | --- | --- | --- | --- | --- | --- |
| n-C11 | --- | 7.57 | 7.69 | --- |  | --- | --- | --- | --- | --- | --- | --- | --- | --- |
| n-C12 | --- | 8.40 | 8.50 | --- |  | --- | --- | --- | --- | --- | --- | --- | --- | --- |
| n-C13 | --- | 9.15 | 9.27 | --- |  | --- | --- | --- | --- | --- | --- | --- | --- | --- |
| n-C14 | --- | 9.86 | 9.97 | --- |  | --- | --- | --- | --- | --- | --- | --- | --- | --- |
| n-C15 | --- | 10.54 | 10.64 | --- |  | --- | --- | --- | --- | --- | --- | --- | --- | --- |
| n-C16 | --- | 11.23 | 11.34 | --- |  | --- | --- | --- | --- | --- | --- | --- | --- | --- |
| n-C17 | --- | 12.05 | 12.16 | --- |  | --- | --- | --- | --- | --- | --- | --- | --- | --- |
| n-C18 | --- | 13.07 | 13.17 | --- |  | --- | --- | --- | --- | --- | --- | --- | --- | --- |
| n-C19 | --- | 14.54 | 14.46 | --- |  | --- | --- | --- | --- | --- | --- | --- | --- | --- |
| n-C20 | --- | 16.04 | 16.13 | --- |  | --- | --- | --- | --- | --- | --- | --- | --- | --- |

[a] All alkanes mentioned are saturated n-alkanes with chain length of *n* carbon atoms measured from standards (Restek or Sigma Aldrich) [b] RI values are provided based on n-alkanes according to the method described in the manuscript, namely equation (1). [c] Retention index are taken from NIST database as experimental standard non-polar or semi-standard non-polar data. Data in brackets are estimated values for non-polar retention indices. [d] Only given for a very rough estimation. It must be mentioned that relative FID area comparison is not suitable for quantifications. In a comparison with known concentrations of similar substance types (organic carbonates, ethers), we obtain response factors that differ by a factor of up to 2. [e] Measured with same retention time in MS onset, x = confirmed. [f] Measured with same EI fragmentation (same 5 main peaks with matching ratio), x = confirmed.

Table 8. Results of selected compounds found in mixtures including Na (with salt).

| solvent mixtures | compound (identified) | retention time (FID) | retention time (MS) | RI^[a]^ | RI from NIST database | verification | | | | | | | |
| --- | --- | --- | --- | --- | --- | --- | --- | --- | --- | --- | --- | --- | --- |
|  |  | [min, Peak maximum] | [min, onset] |  |  | NIST / (Match/ 1000) | Retention, pure compound ^[b]^ | EI fragmentation, pure compound^[c]^ | mass fragmentation *m*/*z* in descending intensity order | | | | |
| PC | Propylene oxide | 2.12 | 2.21 | 489 | 460 | 828 | x | x | 58 | 43 | 57 | 39 | 42 |
| PC | 1,2-Propanediol | 4.04 | 4.16 | 734 | 740 | 886 | x | x | 45 | 43 | 61 | 44 | 58 |
| PC | Isopropyl isobutyrate | 4.63 | 4.75 | 792 | 784 | 877 | x | x | 43 | 71 | 41 | 89 | 115 |
| PC | Diisopropylcarbonate | 5.43 | 5.53 | 868 | (832)^[e]^ | 854 | x | x | 43 | 45 | 63 | 104 | 59 |
| PC+DMC | Propylene oxide | 2.12 | 2.21 | 489 | 460 | 876 | x | x | 58 | 43 | 57 | 39 | 42 |
| PC+DMC | 1,2-Propanediol | 4.04 | 4.16 | 734 | 740 | 891 | x | x | 45 | 43 | 61 | 44 | 58 |
| PC+DMC | **?1** | 6.25 | 6.37 | 954 | --- | --- | --- | --- | 45 | 59 | 72 | 73 | 103 |
| PC+DMC | A | 8.37 | 8.48 | 1198 | --- | ---^[d]^ | --- | --- | 59 | 44 | 103 | 45 | 74 |
| PC+DMC | **?2** | 10.47 | 10.59 | 1493 | --- | --- | --- | --- | 117 | 59 | 44 | 45 | 73 |
| PC+DEC | Ethanol | 1.99 | 2.07 | 437 | 427 | 965 | x | x | 45 | 46 | 43 | --- | --- |
| PC+DEC | Propylene oxide | 2.12 | 2.20 | 489 | 460 | 892 | x | x | 58 | 43 | 57 | 39 | 42 |
| PC+DEC | 1,2-Propanediol | 4.04 | 4.16 | 734 | 740 | 864 | x | x | 45 | 43 | 61 | 44 | 58 |
| PC+DEC | ethyl propyl carbonate | 5.01 | 5.12 | 828 | 861 | 725 | --- | --- | 45 | 43 | 59 | 63 | 90 |
| PC+DEC | C | 9.42 | 9.55 | 1340 | 1348 | 848 | --- | --- | 44 | 45 | 59 | 58 | 104 |
| PC+DEC | **?3** | 11.39 | 11.50 | 1620 | --- | --- | --- | --- | 131 | 103 | 59 | 45 | 91 |
| PC+EMC | Propylene oxide | 2.11 | 2.20 | 485 | 460 | 894 | x | x | 58 | 43 | 57 | 39 | 42 |
| PC+EMC | DMC | 2.89 | 3.00 | 616 | 620 | 940 | x | x | 45 | 59 | 90 | 62 | 60 |
| PC+EMC | 1,2-Propanediol | 4.04 | 4.16 | 437 | 740 | 879 | x | x | 45 | 43 | 61 | 44 | 58 |
| PC+EMC | DEC | 4.57 | 4.65 | 782 | 767 | 931 | x | x | 91 | 45 | 63 | 59 | 75 |
| PC+EMC | A | 8.37 | 8.48 | 1198 | --- | ---^[d]^ | --- | --- | 59 | 44 | 103 | 45 | 74 |
| PC+EMC | B | 8.92 | 9.03 | 1269 | --- | ---^[d]^ | --- | --- | 44 | 45 | 59 | 90 | 103 |
| PC+EMC | C | 9.42 | 9.55 | 1340 | 1348 | 809 | --- | --- | 44 | 45 | 59 | 58 | 104 |
| PC+EMC | **?2** | 10.47 | 10.59 | 1493 | --- | --- | --- | --- | 117 | 59 | 73 | 45 | 118 |
| PC+EMC | **?4** | 10.91 | 11.03 | 1556 | --- | --- | --- | --- | 117 | 59 | 131 | 45 | 103 |
| PC+EMC | **?3** | 11.39 | 11.51 | 1621 | --- | --- | --- | --- | 103 | 131 | 59 | 45 | 91 |
| PC+DPrC | Propylene oxide | 2.12 | 2.21 | 489 | 460 | 899 | x | x | 58 | 43 | 57 | 39 | 42 |
| PC+DPrC | Di-n-propyl ether | 3.53 | 3.64 | 691 | 680 | 802 | --- | --- | 43 | 41 | 73 | 102 | 42 |
| PC+DPrC | n-Propyl acetate | 3.80 | 3.90 | 709 | 708 | 916 | --- | --- | 43 | 61 | 73 | 42 | 59 |
| PC+DPrC | 1,2-Propanediol | 4.04 | 4.16 | 734 | 740 | 869 | x | x | 45 | 43 | 61 | 44 | 58 |
| PC+DPrC |  | 5.95 | 6.06 | 921 | --- | --- | --- | --- | 43 | 45 | 59 | 63 | 41 |
| PC+DPrC | **?5** | 8.86 | 8.99 | 1264 | --- | --- | --- | --- | 43 | 58 | 59 | 100 | 41 |
| PC+DPrC | D | 10.66 | 10.76 | 1517 | (1537)^[e]^ | 828 | --- | --- | 103 | 44 | 59 | 43 | 145 |
| PC+DPrC | **?6** | 13.08 | 13.18 | 1801 | --- | --- | --- | --- | 103 | 44 | 59 | 145 | 43 |
| PC+EC | Propylene oxide | 2.11 | 2.22 | 485 | 460 | 841 | x | x | 58 | 43 | 57 | 39 | 42 |
| PC+EC | Ethylene glycol | 3.56 | 3.67 | 694 | 702 | 723 | x | x | 43 | 62 | 42 | 61 | 33 |
| PC+EC | **?7** | 3.89 | 4.00 | 719 | --- | --- | --- | --- | 103 | 88 | 58 | 118 | 101 |
| PC+EC | 1,2-Propanediol | 4.04 | 4.16 | 734 | 740 | 868 | x | x | 45 | 43 | 61 | 57 | 42 |
| PC+EC | **?8** | 4.25 | 4.36 | 754 | --- | --- | --- | --- | 117 | 89 | 87 | 118 | 59 |
| PC+EC | Diglyme | 6.18 | 6.29 | 945 | 951 | 809 | x | x | 59 | 58 | 45 | 89 | 87 |
| PC+12BC | Propylene oxide | 2.11 | 2.20 | 485 | 460 | 841 | x | x | 58 | 43 | 57 | 39 | 42 |
| PC+12BC | 1,2-Propanediol | 4.04 | 4.16 | 734 | 740 | 870 | x | x | 45 | 43 | 61 | 44 | 58 |
| PC+12BC | 1,2-Butanediol | 5.13 | 5.24 |  | 788 | 832 | x | x | 59 | 41 | 43 | 61 | 58 |
| PC+12BC | Diisopropyl carbonate | 5.43 | 5.53 | 868 | (832)^[e]^ | 840 | --- | --- | 43 | 45 | 41 | 104 | 63 |
| PC+12BC | **?9** | 15.30 | 15.40 | 1956 | --- | --- | --- | --- | 91 | 107 | 79 | 77 | 151 |
| PC+G1 | 1,2-Propanediol | 4.04 | 4.15 | 734 | 740 | 890 | x | x | 45 | 43 | 61 | 58 | 57 |
| PC+G1 | ?8 | 4.25 | 4.34 | 752 |  |  |  |  | 117 | 89 | 59 | 88 | 87 |
| PC+G1 | Diisopropyl carbonate | 5.43 | 5.53 | 868 | (832)^[e]^ | 824 |  |  | 43 | 45 | 41 | 59 | 63 |
| PC+G2 | 1,2-Propanediol | 4.04 | 4.15 | 734 | 740 | 891 | x | x | 45 | 43 | 61 | 58 | 57 |
| PC+G4 | 1,2-Propanediol | 4.04 | 4.15 | 734 | 740 | 874 | x | x | 45 | 43 | 61 | 58 | 57 |
| PC+G4 | ?8 | 4.25 | 4.34 | 752 | --- | --- | --- | --- | 117 | 89 | 59 | 88 | 87 |
| PC+G4 | Triglyme | 8.58 | 8.69 | 1225 | 1232 | 854 | --- | --- | 59 | 58 | 45 | 103 | 89 |
| PC+G4 | **?9** | 9.07 | 9.17 | 1287 | --- | --- | --- | --- | 59 | 45 | 58 | 87 | 73 |
| PC+G4 | Diethylene glycol dibutyl ether | 10.10 | 10.22 | 1437 | (1442)^[e]^ | 788 | x | x | 57 | 41 | 45 | 75 | 85 |
| PC+SL | 1,2-Propanediol | 4.04 | 4.16 | 734 | 740 | 873 | x | x | 45 | 43 | 61 | 57 | 42 |

[a] RI values are provided based on n-alkanes according to the method described in the manuscript, namely equation (1). [b] Measured with same retention time in MS onset, x = confirmed; otherwise (---) no measurements of the pure substance was performed [c] measured and EI fragmentation give same 5 main peaks with matching ratio), x = confirmed. [d] not included in NIST data base. [e] estimated RI index from NIST (no experimental value)

Table 9: Complete results of LCA for all impact categories

| Indicator | PC+G1 | PC+EC | PC+DPrC | PC+SL | PC+G4 | PC+DMC | PC+EMC | PC | PC+G2 | PC +1,2-BC | PC+DEC | Unit |
| --- | --- | --- | --- | --- | --- | --- | --- | --- | --- | --- | --- | --- |
| CED | 8.01E+01 | 7.84E+01 | 8.88E+01 | 8.91E+01 | 6.75E+01 | 7.96E+01 | 9.99E+01 | 9.34E+01 | 7.78E+01 | 8.62E+01 | 1.11E+02 | MJ |
| Fine particulate matter formation | 5.98E-03 | 6.20E-03 | 6.39E-03 | 1.07E-02 | 4.58E-03 | 6.05E-03 | 6.91E-03 | 7.64E-03 | 5.13E-03 | 6.52E-03 | 7.11E-03 | kg PM2.5 eq |
| Fossil resource scarcity | 1.58E+00 | 1.51E+00 | 1.71E+00 | 1.79E+00 | 1.33E+00 | 1.54E+00 | 1.88E+00 | 1.77E+00 | 1.53E+00 | 1.64E+00 | 2.05E+00 | kg oil eq |
| Freshwater ecotoxicity | 1.84E-01 | 2.07E-01 | 1.98E-01 | 1.60E-01 | 1.40E-01 | 2.10E-01 | 2.30E-01 | 2.46E-01 | 1.59E-01 | 2.07E-01 | 2.22E-01 | kg 1,4-DCB |
| Freshwater eutrophication | 1.40E-03 | 1.53E-03 | 1.65E-03 | 1.25E-03 | 1.09E-03 | 1.53E-03 | 2.01E-03 | 1.99E-03 | 1.25E-03 | 1.76E-03 | 2.28E-03 | kg P eq |
| Global warming | 3.70E+00 | 3.93E+00 | 4.06E+00 | 3.38E+00 | 2.85E+00 | 3.86E+00 | 4.12E+00 | 4.77E+00 | 3.26E+00 | 4.26E+00 | 4.05E+00 | kg CO2 eq |
| Human carcinogenic toxicity | 2.70E-01 | 2.92E-01 | 2.88E-01 | 2.48E-01 | 2.04E-01 | 2.94E-01 | 3.62E-01 | 3.51E-01 | 2.31E-01 | 2.99E-01 | 3.32E-01 | kg 1,4-DCB |
| Human non-carcinogenic toxicity | 4.12E+00 | 4.50E+00 | 4.49E+00 | 3.76E+00 | 3.27E+00 | 4.52E+00 | 5.11E+00 | 5.39E+00 | 3.64E+00 | 4.68E+00 | 5.18E+00 | kg 1,4-DCB |
| Ionizing radiation | 3.80E-01 | 4.43E-01 | 5.19E-01 | 3.65E-01 | 3.28E-01 | 4.50E-01 | 7.10E-01 | 6.09E-01 | 3.78E-01 | 5.66E-01 | 8.79E-01 | kBq Co-60 eq |
| Land use | 4.25E-02 | 4.87E-02 | 6.98E-02 | 3.78E-02 | 3.18E-02 | 4.86E-02 | 6.68E-02 | 6.18E-02 | 3.64E-02 | 6.74E-02 | 6.36E-02 | m2a crop eq |
| Marine ecotoxicity | 2.39E-01 | 2.67E-01 | 2.57E-01 | 2.07E-01 | 1.81E-01 | 2.71E-01 | 2.99E-01 | 3.18E-01 | 2.06E-01 | 2.70E-01 | 2.89E-01 | kg 1,4-DCB |
| Marine eutrophication | 1.34E-04 | 1.59E-04 | 1.48E-04 | 1.26E-04 | 1.02E-04 | 1.54E-04 | 1.78E-04 | 2.09E-04 | 1.19E-04 | 2.04E-04 | 1.90E-04 | kg N eq |
| Mineral resource scarcity | 1.43E-02 | 1.57E-02 | 1.48E-02 | 1.27E-02 | 1.15E-02 | 1.60E-02 | 1.71E-02 | 1.76E-02 | 1.26E-02 | 1.58E-02 | 1.55E-02 | kg Cu eq |
| Ozone formation, Human health | 1.36E-02 | 1.52E-02 | 1.57E-02 | 1.96E-02 | 9.61E-03 | 1.45E-02 | 1.52E-02 | 2.23E-02 | 1.15E-02 | 1.44E-02 | 1.44E-02 | kg NOx eq |
| Ozone formation, Terrestrial ecosystems | 1.73E-02 | 1.97E-02 | 2.03E-02 | 2.72E-02 | 1.20E-02 | 1.87E-02 | 1.87E-02 | 3.02E-02 | 1.46E-02 | 1.82E-02 | 1.79E-02 | kg NOx eq |
| Stratospheric ozone depletion | 1.89E-06 | 2.02E-06 | 1.89E-06 | 2.00E-06 | 1.35E-06 | 1.97E-06 | 2.37E-06 | 3.04E-06 | 1.63E-06 | 2.20E-06 | 2.57E-06 | kg CFC11 eq |
| Terrestrial acidification | 1.24E-02 | 1.29E-02 | 1.46E-02 | 2.97E-02 | 9.90E-03 | 1.27E-02 | 1.53E-02 | 1.56E-02 | 1.10E-02 | 1.47E-02 | 1.61E-02 | kg SO2 eq |
| Terrestrial ecotoxicity | 5.65E+00 | 7.39E+00 | 6.24E+00 | 5.39E+00 | 3.95E+00 | 7.04E+00 | 8.12E+00 | 8.50E+00 | 4.48E+00 | 7.55E+00 | 6.20E+00 | kg 1,4-DCB |
| Water consumption | 7.43E-02 | 7.74E-02 | 8.91E-02 | 6.42E-02 | 5.52E-02 | 7.40E-02 | 7.53E-02 | 1.09E-01 | 6.42E-02 | 1.71E-01 | 7.81E-02 | m3 |

Table 10: Cell tests of NMO versus HC (electrolyte: PC + 1 M NaClO_4_)

| cycle | specific discharge capacity / mAh g^-1^ |
| --- | --- |
| 1 | 100.965 |
| 2 | 101.545 |
| 3 | 99.613 |
| 4 | 100 |
| 5 | 99.903 |
| 6 | 99.903 |
| 7 | 96.55 |
| 8 | 96.711 |
| 9 | 96.71 |
| 10 | 96.711 |
| 11 | 96.394 |
| 12 | 96.203 |
| 13 | 96.108 |
| 14 | 96.203 |
| 15 | 96.108 |
| 16 | 95.916 |
| 17 | 96.012 |
| 18 | 95.82 |
| 19 | 95.725 |
| 20 | 95.629 |
| 21 | 95.725 |
| 22 | 95.725 |
| 23 | 95.686 |
| 24 | 95.265 |
| 25 | 95.342 |
| 26 | 95.38 |
| 27 | 95.38 |
| 28 | 95.342 |
| 29 | 95.035 |
| 30 | 94.844 |
| 31 | 95.035 |
| 32 | 94.997 |
| 33 | 94.767 |
| 34 | 94.768 |
| 35 | 94.938 |
| 36 | 94.777 |
| 37 | 94.777 |
| 38 | 94.776 |
| 39 | 94.615 |
| 40 | 94.615 |
| 41 | 94.616 |
| 42 | 94.131 |
| 43 | 94.293 |
| 44 | 94.293 |
| 45 | 93.906 |
| 46 | 93.81 |
| 47 | 93.523 |
| 48 | 93.619 |
| 49 | 93.523 |
| 50 | 93.235 |
| 51 | 93.332 |
| 52 | 93.235 |
| 53 | 93.331 |
| 54 | 93.045 |
| 55 | 92.949 |
| 56 | 92.853 |
| 57 | 92.853 |
| 58 | 92.757 |
| 59 | 92.566 |
| 60 | 92.661 |
| 61 | 92.661 |
| 62 | 92.47 |
| 63 | 92.566 |
| 64 | 92.374 |
| 65 | 92.183 |
| 66 | 92.183 |
| 67 | 92.279 |
| 68 | 91.704 |
| 69 | 91.896 |
| 70 | 91.991 |
| 71 | 91.704 |
| 72 | 91.8 |
| 73 | 91.417 |
| 74 | 91.608 |
| 75 | 91.321 |
| 76 | 91.417 |
| 77 | 91.321 |
| 78 | 91.13 |
| 79 | 90.938 |
| 80 | 91.034 |
| 81 | 91.034 |
| 82 | 91.034 |
| 83 | 90.555 |
| 84 | 90.747 |
| 85 | 90.938 |
| 86 | 90.843 |
| 87 | 90.747 |
| 88 | 90.555 |
| 89 | 90.556 |
| 90 | 90.554 |
| 91 | 89.981 |
| 92 | 90.46 |
| 93 | 90.077 |
| 94 | 90.077 |
| 95 | 89.981 |
| 96 | 90.077 |
| 97 | 89.79 |
| 98 | 89.981 |
| 99 | 89.789 |
| 100 | 89.503 |
| 101 | 89.598 |
| 102 | 89.503 |
| 103 | 89.503 |
| 104 | 89.503 |
| 105 | 89.215 |
| 106 | 89.407 |
| 107 | 89.215 |
| 108 | 89.215 |
| 109 | 89.216 |
| 110 | 89.024 |
| 111 | 89.024 |
| 112 | 89.024 |
| 113 | 88.928 |
| 114 | 88.832 |
| 115 | 88.545 |
| 116 | 88.832 |
| 117 | 88.737 |
| 118 | 88.737 |
| 119 | 88.545 |
| 120 | 88.258 |
| 121 | 88.449 |
| 122 | 88.45 |
| 123 | 88.162 |
| 124 | 88.067 |
| 125 | 88.067 |
| 126 | 88.068 |
| 127 | 88.162 |
| 128 | 87.779 |
| 129 | 87.971 |
| 130 | 87.588 |
| 131 | 87.684 |
| 132 | 87.588 |
| 133 | 87.588 |
| 134 | 87.301 |
| 135 | 87.397 |
| 136 | 87.588 |
| 137 | 87.397 |
| 138 | 87.301 |
| 139 | 87.397 |
| 140 | 87.014 |
| 141 | 87.109 |
| 142 | 87.014 |
| 143 | 87.014 |
| 144 | 87.013 |
| 145 | 88.067 |
| 146 | 87.971 |
| 147 | 87.683 |
| 148 | 87.301 |
| 149 | 87.397 |
| 150 | 87.301 |
| 151 | 87.109 |
| 152 | 87.109 |
| 153 | 86.631 |
| 154 | 86.822 |
| 155 | 86.535 |
| 156 | 86.535 |
| 157 | 86.343 |
| 158 | 86.439 |
| 159 | 86.343 |
| 160 | 86.343 |
| 161 | 85.865 |
| 162 | 85.769 |
| 163 | 85.961 |
| 164 | 85.769 |
| 165 | 85.578 |
| 166 | 85.482 |
| 167 | 85.386 |
| 168 | 85.482 |
| 169 | 85.099 |
| 170 | 85.291 |
| 171 | 85.291 |
| 172 | 85.195 |
| 173 | 84.906 |
| 174 | 84.716 |
| 175 | 84.812 |
| 176 | 84.812 |
| 177 | 84.525 |
| 178 | 84.333 |
| 179 | 84.525 |
| 180 | 84.333 |
| 181 | 84.429 |
| 182 | 84.333 |
| 183 | 84.237 |
| 184 | 84.142 |
| 185 | 84.142 |
| 186 | 83.855 |
| 187 | 83.95 |
| 188 | 83.855 |
| 189 | 83.568 |
| 190 | 83.759 |
| 191 | 83.472 |
| 192 | 83.472 |
| 193 | 83.472 |
| 194 | 83.472 |
| 195 | 83.567 |
| 196 | 83.089 |
| 197 | 82.993 |
| 198 | 82.993 |
| 199 | 82.898 |
| 200 | 82.993 |
| 201 | 83.089 |
| 202 | 82.419 |
| 203 | 82.706 |
| 204 | 82.419 |
| 205 | 82.61 |
| 206 | 82.61 |
| 207 | 82.323 |
| 208 | 82.323 |
| 209 | 82.419 |
| 210 | 82.132 |
| 211 | 82.227 |
| 212 | 82.419 |
| 213 | 81.749 |
| 214 | 81.749 |
| 215 | 81.844 |
| 216 | 81.749 |
| 217 | 81.558 |
| 218 | 81.653 |
| 219 | 81.94 |
| 220 | 81.749 |
| 221 | 82.036 |
| 222 | 81.366 |
| 223 | 81.174 |
| 224 | 81.174 |
| 225 | 81.653 |
| 226 | 81.653 |
| 227 | 81.078 |
| 228 | 81.27 |
| 229 | 80.887 |
| 230 | 81.079 |
| 231 | 81.27 |
| 232 | 80.792 |
| 233 | 80.696 |
| 234 | 80.6 |
| 235 | 81.174 |
| 236 | 81.079 |
| 237 | 80.887 |
| 238 | 80.504 |
| 239 | 80.217 |
| 240 | 80.696 |
| 241 | 80.504 |
| 242 | 80.504 |
| 243 | 80.696 |
| 244 | 80.504 |

Table 11: TGA and DCS results of the electrolyte EC + PC + 1 M NaClO_4_

| Time / min | Temperature / °C | DSC / mW/mg | Mass / % | Gas Flow  (purge1) / (ml/min) | Gas Flow  (protective) / (ml/min) | Sensit./(uV/mW) |
| --- | --- | --- | --- | --- | --- | --- |
| 0 | 21.715 | 0.003 | 100 | 50 | 20 | 1.016 |
| 0.25 | 21.716 | 0.004 | 99.992 | 50 | 20 | 1.016 |
| 0.5 | 21.801 | 0.005 | 99.994 | 50 | 20 | 1.016 |
| 0.75 | 22.037 | 0.009 | 99.992 | 50 | 20 | 1.016 |
| 1 | 22.372 | 0.014 | 99.972 | 50 | 20 | 1.016 |
| 1.25 | 22.892 | 0.021 | 99.988 | 50 | 20 | 1.015 |
| 1.5 | 23.555 | 0.028 | 99.967 | 50 | 20 | 1.015 |
| 1.75 | 24.288 | 0.035 | 99.973 | 50 | 20 | 1.014 |
| 2 | 25.136 | 0.042 | 99.972 | 50 | 20 | 1.013 |
| 2.25 | 26.078 | 0.048 | 99.964 | 50 | 20 | 1.012 |
| 2.5 | 27.119 | 0.053 | 99.928 | 50 | 20 | 1.011 |
| 2.75 | 28.287 | 0.058 | 99.953 | 50 | 20 | 1.01 |
| 3 | 29.614 | 0.063 | 99.937 | 50 | 20 | 1.009 |
| 3.25 | 31.06 | 0.068 | 99.953 | 50 | 20 | 1.008 |
| 3.5 | 32.622 | 0.073 | 99.939 | 50 | 20 | 1.006 |
| 3.75 | 34.319 | 0.078 | 99.944 | 50 | 20 | 1.005 |
| 4 | 36.182 | 0.083 | 99.919 | 50 | 20 | 1.003 |
| 4.25 | 38.162 | 0.087 | 99.912 | 50 | 20 | 1.002 |
| 4.5 | 40.27 | 0.09 | 99.916 | 50 | 20 | 1 |
| 4.75 | 42.472 | 0.093 | 99.882 | 50 | 20 | 0.998 |
| 5 | 44.796 | 0.095 | 99.9 | 50 | 20 | 0.996 |
| 5.25 | 47.267 | 0.096 | 99.887 | 50 | 20 | 0.993 |
| 5.5 | 49.816 | 0.097 | 99.882 | 50 | 20 | 0.991 |
| 5.75 | 52.48 | 0.096 | 99.826 | 50 | 20 | 0.989 |
| 6 | 55.23 | 0.095 | 99.844 | 50 | 20 | 0.986 |
| 6.25 | 58.088 | 0.094 | 99.851 | 50 | 20 | 0.984 |
| 6.5 | 61.012 | 0.092 | 99.837 | 50 | 20 | 0.981 |
| 6.75 | 64.006 | 0.089 | 99.781 | 50 | 20 | 0.978 |
| 7 | 67.113 | 0.086 | 99.818 | 50 | 20 | 0.976 |
| 7.25 | 70.25 | 0.082 | 99.746 | 50 | 20 | 0.973 |
| 7.5 | 73.445 | 0.078 | 99.748 | 50 | 20 | 0.97 |
| 7.75 | 76.66 | 0.073 | 99.723 | 50 | 20 | 0.967 |
| 8 | 79.935 | 0.069 | 99.689 | 50 | 20 | 0.964 |
| 8.25 | 83.248 | 0.064 | 99.619 | 50 | 20 | 0.961 |
| 8.5 | 86.632 | 0.058 | 99.566 | 50 | 20 | 0.958 |
| 8.75 | 90.025 | 0.052 | 99.54 | 50 | 20 | 0.955 |
| 9 | 93.434 | 0.044 | 99.446 | 50 | 20 | 0.952 |
| 9.25 | 96.867 | 0.036 | 99.387 | 50 | 20 | 0.949 |
| 9.5 | 100.333 | 0.028 | 99.288 | 50 | 20 | 0.946 |
| 9.75 | 103.777 | 0.019 | 99.208 | 50 | 20 | 0.943 |
| 10 | 107.21 | 0.01 | 99.065 | 50 | 20 | 0.94 |
| 10.25 | 110.626 | 0.002 | 98.936 | 50 | 20 | 0.936 |
| 10.5 | 114.038 | -0.006 | 98.772 | 50 | 20 | 0.933 |
| 10.75 | 117.456 | -0.013 | 98.567 | 50 | 20 | 0.93 |
| 11 | 120.823 | -0.019 | 98.36 | 50 | 20 | 0.927 |
| 11.25 | 124.187 | -0.025 | 98.113 | 50 | 20 | 0.924 |
| 11.5 | 127.575 | -0.028 | 97.812 | 50 | 20 | 0.921 |
| 11.75 | 130.914 | -0.029 | 97.46 | 50 | 20 | 0.918 |
| 12 | 134.239 | -0.029 | 97.069 | 50 | 20 | 0.915 |
| 12.25 | 137.517 | -0.026 | 96.595 | 50 | 20 | 0.912 |
| 12.5 | 140.775 | -0.021 | 96.076 | 50 | 20 | 0.909 |
| 12.75 | 144.018 | -0.014 | 95.449 | 50 | 20 | 0.907 |
| 13 | 147.221 | -0.003 | 94.758 | 50 | 20 | 0.904 |
| 13.25 | 150.376 | 0.013 | 93.885 | 50 | 20 | 0.901 |
| 13.5 | 153.494 | 0.034 | 92.936 | 50 | 20 | 0.898 |
| 13.75 | 156.562 | 0.061 | 91.727 | 50 | 20 | 0.895 |
| 14 | 159.639 | 0.095 | 90.422 | 50 | 20 | 0.893 |
| 14.25 | 162.652 | 0.133 | 88.879 | 50 | 20 | 0.89 |
| 14.5 | 165.654 | 0.174 | 87.115 | 50 | 20 | 0.887 |
| 14.75 | 168.578 | 0.228 | 85.062 | 50 | 20 | 0.885 |
| 15 | 171.485 | 0.275 | 82.839 | 50 | 20 | 0.882 |
| 15.25 | 174.345 | 0.331 | 80.327 | 50 | 20 | 0.88 |
| 15.5 | 177.173 | 0.369 | 77.711 | 50 | 20 | 0.877 |
| 15.75 | 179.968 | 0.408 | 74.79 | 50 | 20 | 0.875 |
| 16 | 182.726 | 0.444 | 71.784 | 50 | 20 | 0.872 |
| 16.25 | 185.483 | 0.484 | 68.576 | 50 | 20 | 0.87 |
| 16.5 | 188.205 | 0.547 | 64.957 | 50 | 20 | 0.868 |
| 16.75 | 190.906 | 0.617 | 60.986 | 50 | 20 | 0.865 |
| 17 | 193.574 | 0.673 | 56.813 | 50 | 20 | 0.863 |
| 17.25 | 196.205 | 0.727 | 52.323 | 50 | 20 | 0.861 |
| 17.5 | 198.84 | 0.788 | 47.162 | 50 | 20 | 0.858 |
| 17.75 | 201.426 | 0.841 | 41.929 | 50 | 20 | 0.856 |
| 18 | 203.961 | 0.889 | 36.62 | 50 | 20 | 0.854 |
| 18.25 | 206.474 | 0.927 | 31.905 | 50 | 20 | 0.852 |
| 18.5 | 208.99 | 0.92 | 26.588 | 50 | 20 | 0.85 |
| 18.75 | 211.43 | 0.749 | 22.439 | 50 | 20 | 0.848 |
| 19 | 213.935 | 0.452 | 19.458 | 50 | 20 | 0.845 |
| 19.25 | 216.62 | 0.069 | 17.709 | 50 | 20 | 0.843 |
| 19.5 | 219.455 | -0.267 | 17.67 | 50 | 20 | 0.841 |
| 19.75 | 222.345 | -0.398 | 17.137 | 50 | 20 | 0.838 |
| 20 | 225.144 | -0.441 | 16.649 | 50 | 20 | 0.836 |
| 20.25 | 227.844 | -0.456 | 16.452 | 50 | 20 | 0.834 |
| 20.5 | 230.508 | -0.455 | 16.301 | 50 | 20 | 0.831 |
| 20.75 | 233.131 | -0.458 | 15.992 | 50 | 20 | 0.829 |
| 21 | 235.702 | -0.457 | 15.692 | 50 | 20 | 0.827 |
| 21.25 | 238.21 | -0.339 | 14.401 | 50 | 20 | 0.825 |
| 21.5 | 240.698 | -0.412 | 13.896 | 50 | 20 | 0.823 |
| 21.75 | 243.186 | -0.382 | 13.287 | 50 | 20 | 0.821 |
| 22 | 245.635 | -0.322 | 12.334 | 50 | 20 | 0.819 |
| 22.25 | 248.016 | -0.27 | 11.413 | 50 | 20 | 0.817 |
| 22.5 | 250.435 | -0.167 | 10.171 | 50 | 20 | 0.815 |
| 22.75 | 252.917 | -0.093 | 9.041 | 50 | 20 | 0.813 |
| 23 | 255.338 | -0.068 | 8.24 | 50 | 20 | 0.811 |
| 23.25 | 257.798 | -0.095 | 7.985 | 50 | 20 | 0.809 |
| 23.5 | 260.273 | -0.121 | 7.945 | 50 | 20 | 0.807 |
| 23.75 | 262.75 | -0.126 | 7.955 | 50 | 20 | 0.804 |
| 24 | 265.255 | -0.127 | 7.932 | 50 | 20 | 0.802 |
| 24.25 | 267.742 | -0.126 | 7.931 | 50 | 20 | 0.8 |
| 24.5 | 270.267 | -0.108 | 7.916 | 50 | 20 | 0.798 |
| 24.75 | 272.767 | -0.1 | 7.949 | 50 | 20 | 0.796 |
| 25 | 275.31 | -0.1 | 7.93 | 50 | 20 | 0.794 |
| 25.25 | 277.823 | -0.098 | 7.91 | 50 | 20 | 0.792 |
| 25.5 | 280.363 | -0.103 | 7.913 | 50 | 20 | 0.79 |
| 25.75 | 282.901 | -0.109 | 7.89 | 50 | 20 | 0.788 |
| 26 | 285.379 | -0.109 | 7.879 | 50 | 20 | 0.786 |
| 26.25 | 287.862 | -0.109 | 7.88 | 50 | 20 | 0.784 |
| 26.5 | 290.301 | -0.108 | 7.879 | 50 | 20 | 0.782 |
| 26.75 | 292.741 | -0.107 | 7.895 | 50 | 20 | 0.78 |
| 27 | 295.184 | -0.106 | 7.887 | 50 | 20 | 0.778 |
| 27.25 | 297.632 | -0.105 | 7.864 | 50 | 20 | 0.776 |
| 27.5 | 300.062 | -0.102 | 7.912 | 50 | 20 | 0.774 |
| 27.75 | 302.485 | -0.086 | 7.904 | 50 | 20 | 0.772 |
| 28 | 304.927 | -0.096 | 7.885 | 50 | 20 | 0.771 |
| 28.25 | 307.385 | -0.105 | 7.928 | 50 | 20 | 0.769 |
| 28.5 | 309.844 | -0.107 | 7.932 | 50 | 20 | 0.767 |
| 28.75 | 312.273 | -0.108 | 7.899 | 50 | 20 | 0.765 |
| 29 | 314.77 | -0.109 | 7.903 | 50 | 20 | 0.763 |
| 29.25 | 317.214 | -0.109 | 7.935 | 50 | 20 | 0.761 |
| 29.5 | 319.67 | -0.109 | 7.865 | 50 | 20 | 0.759 |
| 29.75 | 322.127 | -0.108 | 7.941 | 50 | 20 | 0.757 |
| 30 | 324.62 | -0.108 | 7.936 | 50 | 20 | 0.755 |
| 30.25 | 327.061 | -0.107 | 7.97 | 50 | 20 | 0.753 |
| 30.5 | 329.522 | -0.107 | 7.966 | 50 | 20 | 0.751 |
| 30.75 | 331.987 | -0.106 | 7.965 | 50 | 20 | 0.75 |
| 31 | 334.437 | -0.106 | 7.978 | 50 | 20 | 0.748 |
| 31.25 | 336.927 | -0.106 | 7.972 | 50 | 20 | 0.746 |
| 31.5 | 339.385 | -0.106 | 7.943 | 50 | 20 | 0.744 |
| 31.75 | 341.854 | -0.105 | 8.027 | 50 | 20 | 0.742 |
| 32 | 344.333 | -0.105 | 7.977 | 50 | 20 | 0.74 |
| 32.25 | 346.793 | -0.105 | 8.014 | 50 | 20 | 0.738 |
| 32.5 | 349.267 | -0.105 | 8.001 | 50 | 20 | 0.737 |
| 32.75 | 351.732 | -0.105 | 8.008 | 50 | 20 | 0.735 |
| 33 | 354.233 | -0.106 | 7.983 | 50 | 20 | 0.733 |
| 33.25 | 356.715 | -0.105 | 8.007 | 50 | 20 | 0.731 |
| 33.5 | 359.163 | -0.104 | 8.07 | 50 | 20 | 0.729 |
| 33.75 | 361.652 | -0.105 | 8.034 | 50 | 20 | 0.727 |
| 34 | 364.136 | -0.105 | 8.053 | 50 | 20 | 0.726 |
| 34.25 | 366.627 | -0.107 | 7.963 | 50 | 20 | 0.724 |
| 34.5 | 369.099 | -0.106 | 8.032 | 50 | 20 | 0.722 |
| 34.75 | 371.584 | -0.106 | 8.014 | 50 | 20 | 0.72 |
| 35 | 374.064 | -0.107 | 7.982 | 50 | 20 | 0.718 |
| 35.25 | 376.546 | -0.107 | 8.018 | 50 | 20 | 0.717 |
| 35.5 | 379.014 | -0.106 | 8.048 | 50 | 20 | 0.715 |
| 35.75 | 381.516 | -0.107 | 7.885 | 50 | 20 | 0.713 |
| 36 | 383.969 | -0.106 | 8.104 | 50 | 20 | 0.711 |
| 36.25 | 386.46 | -0.105 | 8.079 | 50 | 20 | 0.71 |
| 36.5 | 388.957 | -0.104 | 8.041 | 50 | 20 | 0.708 |
| 36.75 | 391.425 | -0.103 | 8.071 | 50 | 20 | 0.706 |
| 37 | 393.907 | -0.102 | 8.161 | 50 | 20 | 0.704 |
| 37.25 | 396.389 | -0.103 | 7.95 | 50 | 20 | 0.703 |
| 37.5 | 398.876 | -0.103 | 8.002 | 50 | 20 | 0.701 |
| 37.75 | 401.373 | -0.104 | 7.983 | 50 | 20 | 0.699 |
| 38 | 403.842 | -0.103 | 7.968 | 50 | 20 | 0.697 |
| 38.25 | 406.339 | -0.103 | 8.02 | 50 | 20 | 0.696 |
| 38.5 | 408.813 | -0.104 | 7.974 | 50 | 20 | 0.694 |
| 38.75 | 411.292 | -0.104 | 7.995 | 50 | 20 | 0.692 |
| 39 | 413.793 | -0.104 | 7.976 | 50 | 20 | 0.691 |
| 39.25 | 416.28 | -0.103 | 7.987 | 50 | 20 | 0.689 |
| 39.5 | 418.758 | -0.104 | 7.902 | 50 | 20 | 0.687 |
| 39.75 | 421.258 | -0.104 | 7.927 | 50 | 20 | 0.686 |
| 40 | 423.735 | -0.104 | 7.909 | 50 | 20 | 0.684 |
| 40.25 | 426.221 | -0.105 | 7.903 | 50 | 20 | 0.682 |
| 40.5 | 428.712 | -0.104 | 7.917 | 50 | 20 | 0.681 |
| 40.75 | 431.198 | -0.103 | 7.917 | 50 | 20 | 0.679 |
| 41 | 433.668 | -0.102 | 7.926 | 50 | 20 | 0.677 |
| 41.25 | 436.174 | -0.102 | 7.923 | 50 | 20 | 0.676 |
| 41.5 | 438.658 | -0.1 | 7.956 | 50 | 20 | 0.674 |
| 41.75 | 441.151 | -0.101 | 7.912 | 50 | 20 | 0.673 |
| 42 | 443.653 | -0.099 | 7.94 | 50 | 20 | 0.671 |
| 42.25 | 446.121 | -0.098 | 7.95 | 50 | 20 | 0.669 |
| 42.5 | 448.627 | -0.097 | 7.913 | 50 | 20 | 0.668 |
| 42.75 | 451.114 | -0.095 | 7.941 | 50 | 20 | 0.666 |
| 43 | 453.606 | -0.093 | 7.923 | 50 | 20 | 0.665 |
| 43.25 | 456.092 | -0.089 | 7.958 | 50 | 20 | 0.663 |
| 43.5 | 458.592 | -0.086 | 7.921 | 50 | 20 | 0.661 |
| 43.75 | 461.095 | -0.082 | 7.916 | 50 | 20 | 0.66 |
| 44 | 463.562 | -0.075 | 7.934 | 50 | 20 | 0.658 |
| 44.25 | 466.048 | -0.06 | 7.933 | 50 | 20 | 0.657 |
| 44.5 | 468.548 | -0.018 | 7.952 | 50 | 20 | 0.655 |
| 44.75 | 471.012 | 0.066 | 7.939 | 50 | 20 | 0.654 |
| 45 | 473.457 | 0.159 | 7.93 | 50 | 20 | 0.652 |
| 45.25 | 475.95 | 0.067 | 7.97 | 50 | 20 | 0.651 |
| 45.5 | 478.476 | 0.048 | 7.976 | 50 | 20 | 0.649 |
| 45.75 | 480.994 | 0.041 | 7.966 | 50 | 20 | 0.648 |
| 46 | 483.468 | 0.039 | 7.942 | 50 | 20 | 0.646 |
| 46.25 | 485.991 | 0.039 | 7.945 | 50 | 20 | 0.645 |
| 46.5 | 488.484 | 0.04 | 7.984 | 50 | 20 | 0.643 |
| 46.75 | 490.995 | 0.041 | 7.932 | 50 | 20 | 0.642 |
| 47 | 493.504 | 0.04 | 7.947 | 50 | 20 | 0.64 |
| 47.25 | 496.007 | 0.042 | 7.996 | 50 | 20 | 0.639 |
| 47.5 | 498.5 | 0.042 | 7.978 | 50 | 20 | 0.637 |
| 47.75 | 500.995 | 0.042 | 7.908 | 50 | 20 | 0.636 |
| 48 | 503.485 | 0.043 | 7.932 | 50 | 20 | 0.634 |
| 48.25 | 505.979 | 0.051 | 7.92 | 50 | 20 | 0.633 |
| 48.5 | 508.491 | 0.05 | 7.893 | 50 | 20 | 0.632 |
| 48.75 | 510.96 | 0.053 | 7.931 | 50 | 20 | 0.63 |
| 49 | 513.471 | 0.048 | 7.927 | 50 | 20 | 0.629 |
| 49.25 | 515.973 | 0.047 | 7.897 | 50 | 20 | 0.627 |
| 49.5 | 518.462 | 0.046 | 7.925 | 50 | 20 | 0.626 |
| 49.75 | 520.964 | 0.046 | 7.899 | 50 | 20 | 0.624 |
| 50 | 523.452 | 0.046 | 7.881 | 50 | 20 | 0.623 |
| 50.25 | 525.947 | 0.044 | 7.86 | 50 | 20 | 0.622 |
| 50.5 | 528.459 | 0.043 | 7.86 | 50 | 20 | 0.62 |
| 50.75 | 530.946 | 0.042 | 7.848 | 50 | 20 | 0.619 |
| 51 | 533.457 | 0.04 | 7.816 | 50 | 20 | 0.618 |
| 51.25 | 535.943 | 0.038 | 7.852 | 50 | 20 | 0.616 |
| 51.5 | 538.442 | 0.035 | 7.791 | 50 | 20 | 0.615 |
| 51.75 | 540.935 | 0.034 | 7.754 | 50 | 20 | 0.614 |
| 52 | 543.432 | 0.032 | 7.735 | 50 | 20 | 0.612 |
| 52.25 | 545.92 | 0.029 | 7.712 | 50 | 20 | 0.611 |
| 52.5 | 548.419 | 0.026 | 7.686 | 50 | 20 | 0.61 |
| 52.75 | 550.922 | 0.019 | 7.544 | 50 | 20 | 0.608 |
| 53 | 553.441 | 0.009 | 7.503 | 50 | 20 | 0.607 |
| 53.25 | 555.92 | -0.002 | 7.49 | 50 | 20 | 0.606 |
| 53.5 | 558.425 | -0.015 | 7.349 | 50 | 20 | 0.604 |
| 53.75 | 560.93 | -0.028 | 7.262 | 50 | 20 | 0.603 |
| 54 | 563.415 | -0.043 | 7.151 | 50 | 20 | 0.602 |
| 54.25 | 565.923 | -0.058 | 7.033 | 50 | 20 | 0.6 |
| 54.5 | 568.421 | -0.073 | 6.861 | 50 | 20 | 0.599 |
| 54.75 | 570.941 | -0.088 | 6.675 | 50 | 20 | 0.598 |
| 55 | 573.442 | -0.103 | 6.455 | 50 | 20 | 0.597 |
| 55.25 | 575.938 | -0.111 | 6.269 | 50 | 20 | 0.595 |
| 55.5 | 578.442 | -0.126 | 5.969 | 50 | 20 | 0.594 |
| 55.75 | 580.948 | -0.145 | 5.649 | 50 | 20 | 0.593 |
| 56 | 583.441 | -0.255 | 5.331 | 50 | 20 | 0.592 |
| 56.25 | 585.974 | -0.265 | 5.043 | 50 | 20 | 0.59 |
| 56.5 | 588.451 | -0.259 | 4.77 | 50 | 20 | 0.589 |
| 56.75 | 590.968 | -0.246 | 4.504 | 50 | 20 | 0.588 |
| 57 | 593.444 | -0.23 | 4.294 | 50 | 20 | 0.587 |
| 57.25 | 595.931 | -0.213 | 4.116 | 50 | 20 | 0.586 |
| 57.5 | 598.432 | -0.196 | 3.95 | 50 | 20 | 0.584 |
| 57.75 | 600.913 | -0.179 | 3.811 | 50 | 20 | 0.583 |
| 58 | 603.391 | -0.162 | 3.756 | 50 | 20 | 0.582 |
| 58.25 | 605.885 | -0.148 | 3.695 | 50 | 20 | 0.581 |
| 58.5 | 608.383 | -0.137 | 3.627 | 50 | 20 | 0.58 |
| 58.75 | 610.875 | -0.13 | 3.617 | 50 | 20 | 0.578 |
| 59 | 613.378 | -0.127 | 3.602 | 50 | 20 | 0.577 |
| 59.25 | 615.879 | -0.127 | 3.576 | 50 | 20 | 0.576 |
| 59.5 | 618.358 | -0.129 | 3.559 | 50 | 20 | 0.575 |
| 59.75 | 620.859 | -0.13 | 3.559 | 50 | 20 | 0.574 |
| 60 | 623.361 | -0.131 | 3.524 | 50 | 20 | 0.573 |
| 60.25 | 625.835 | -0.131 | 3.563 | 50 | 20 | 0.572 |
| 60.5 | 628.359 | -0.132 | 3.562 | 50 | 20 | 0.57 |
| 60.75 | 630.844 | -0.133 | 3.547 | 50 | 20 | 0.569 |
| 61 | 633.333 | -0.135 | 3.544 | 50 | 20 | 0.568 |
| 61.25 | 635.851 | -0.135 | 3.548 | 50 | 20 | 0.567 |
| 61.5 | 638.35 | -0.136 | 3.535 | 50 | 20 | 0.566 |
| 61.75 | 640.841 | -0.135 | 3.534 | 50 | 20 | 0.565 |
| 62 | 643.337 | -0.135 | 3.549 | 50 | 20 | 0.564 |
| 62.25 | 645.849 | -0.135 | 3.545 | 50 | 20 | 0.563 |
| 62.5 | 648.331 | -0.135 | 3.492 | 50 | 20 | 0.562 |
| 62.75 | 650.824 | -0.133 | 3.536 | 50 | 20 | 0.561 |
| 63 | 653.347 | -0.133 | 3.527 | 50 | 20 | 0.56 |
| 63.25 | 655.823 | -0.132 | 3.539 | 50 | 20 | 0.559 |
| 63.5 | 658.328 | -0.131 | 3.536 | 50 | 20 | 0.557 |
| 63.75 | 660.809 | -0.13 | 3.509 | 50 | 20 | 0.556 |
| 64 | 663.31 | -0.128 | 3.531 | 50 | 20 | 0.555 |
| 64.25 | 665.812 | -0.126 | 3.528 | 50 | 20 | 0.554 |
| 64.5 | 668.301 | -0.124 | 3.54 | 50 | 20 | 0.553 |
| 64.75 | 670.807 | -0.123 | 3.506 | 50 | 20 | 0.552 |
| 65 | 673.294 | -0.12 | 3.526 | 50 | 20 | 0.551 |
| 65.25 | 675.806 | -0.118 | 3.513 | 50 | 20 | 0.55 |
| 65.5 | 678.316 | -0.115 | 3.531 | 50 | 20 | 0.549 |
| 65.75 | 680.814 | -0.111 | 3.517 | 50 | 20 | 0.548 |
| 66 | 683.303 | -0.108 | 3.501 | 50 | 20 | 0.547 |
| 66.25 | 685.786 | -0.105 | 3.517 | 50 | 20 | 0.546 |
| 66.5 | 688.279 | -0.101 | 3.499 | 50 | 20 | 0.545 |
| 66.75 | 690.779 | -0.096 | 3.518 | 50 | 20 | 0.544 |
| 67 | 693.293 | -0.092 | 3.521 | 50 | 20 | 0.543 |
| 67.25 | 695.799 | -0.087 | 3.496 | 50 | 20 | 0.542 |
| 67.5 | 698.315 | -0.083 | 3.499 | 50 | 20 | 0.541 |
| 67.75 | 700.797 | -0.078 | 3.501 | 50 | 20 | 0.54 |

Table 12. Identification number and substance info card of the chemicals assessed.

| **Substance** | **European Community number** | **Link to substance Infocard** |
| --- | --- | --- |
| Sodium Perchlorate | 231-511-9 | https://echa.europa.eu/substance-information/-/substanceinfo/100.028.647 |
| Propylene Carbonate | 203-572-1 | https://echa.europa.eu/substance-information/-/substanceinfo/100.003.248 |
| Ethylene Carbonate | 202-510-0 | https://echa.europa.eu/substance-information/-/substanceinfo/100.002.283 |
| 1,2 Butylene Carbonate | 403-780-4 | https://echa.europa.eu/substance-information/-/substanceinfo/100.100.548 |
| Diethyl Carbonate | 203-311-1 | https://echa.europa.eu/substance-information/-/substanceinfo/100.003.011 |
| Dimethyl Carbonate | 210-478-4 | https://echa.europa.eu/substance-information/-/substanceinfo/100.009.527 |
| Ethylmethyl Carbonate | 433-480-9 | https://echa.europa.eu/substance-information/-/substanceinfo/100.103.173 |
| Dipropyl Carbonate | 210-822-3 | https://echa.europa.eu/substance-information/-/substanceinfo/100.009.839 |
| Sulfolane | 204-783-1 | https://echa.europa.eu/substance-information/-/substanceinfo/100.004.349 |
| Monoglyme | 203-794-9 | https://echa.europa.eu/substance-information/-/substanceinfo/100.003.451 |
| Diglyme | 203-924-4 | https://echa.europa.eu/substance-information/-/substanceinfo/100.003.568 |
| Tetraglyme | 205-594-7 | https://echa.europa.eu/substance-information/-/substanceinfo/100.005.086 |
