# Supplementary material for: Dataset of propylene carbonate based liquid electrolyte mixtures for sodium-ion cells
Source: Data Brief. 2021 Dec 30;40:107775. doi: 10.1016/j.dib.2021.107775 (PMC8741479; doi:10.1016/j.dib.2021.107775)
Supplement: Supplementary file 2 [file mmc2.docx]

Table 1a

PC + 1 M NaClO4

T d D_Na conductivity viscosity

°C g∙cm-3 cm2∙s-1 mS∙cm-1 mPa∙s

0 1.2933 2.08E-7 2.88 15.12

5 1.2885 2.88E-7 3.44 12.66

10 1.2829 3.91E-7 4.05 10.75

15 1.2782 5.18E-7 4.71 9.24

20 1.2731 6.77E-7 5.41 8.02

25 1.2679 8.73E-7 6.17 7.02

30 1.2631 1.11E-6 6.99 6.18

35 1.2574 1.41E-6 7.88 5.47

40 1.2528 1.76E-6 8.82 4.88

45 1.2481 2.18E-6 9.82 4.37

50 1.2426 2.67E-6 10.86 3.94

55 1.2377 3.23E-6 11.93 3.58

60 1.2331 3.85E-6 12.99 3.28

65 1.2274 4.49E-6 14.00 3.04

70 1.2227 5.12E-6 14.90 2.85

75 1.2180 5.69E-6 15.63 2.72

80 1.2130 6.10E-6 16.12 2.64

85 1.2083 6.29E-6 16.28 2.61

90 1.2030 6.18E-6 16.05 2.66

Table 1b

PC + 1 M NaClO4 PC + DMC + 1 M NaClO4

T d D_Na conductivity viscosity

°C g∙cm-3 cm2∙s-1 mS∙cm-1 mPa∙s

0 1.2284 5.75E-7 6.01 6.16

5 1.2235 7.25E-7 6.77 5.45

10 1.2180 9.03E-7 7.58 4.86

15 1.2130 1.11E-6 8.43 4.36

20 1.2077 1.36E-6 9.33 3.93

25 1.2026 1.64E-6 10.28 3.56

30 1.1974 1.98E-6 11.29 3.24

35 1.1927 2.37E-6 12.36 2.95

40 1.1874 2.82E-6 13.48 2.70

45 1.1825 3.34E-6 14.66 2.47

50 1.1769 3.93E-6 15.89 2.29

55 1.1722 4.59E-6 17.15 2.12

60 1.1674 5.30E-6 18.41 1.96

65 1.1620 6.06E-6 19.66 1.84

70 1.1570 6.84E-6 20.85 1.74

75 11.1517 7.42E-6 21.39 1.68

80 1.1471 8.10E-6 22.31 1.61

85 1.1418 8.67E-6 23.01 1.56

90 1.1369 9.05E-6 23.45 1.53

Table 1c

PC + DEC + 1 M NaClO4

T d D_Na conductivity viscosity

°C g∙cm-3 cm2∙s-1 mS∙cm-1 mPa∙s

0 1.1638 4.66E-7 4.51 7.42

5 1.1589 5.90E-7 5.10 6.52

10 1.1536 7.39E-7 5.72 5.78

15 1.1485 9.15E-7 6.38 5.16

20 1.1434 1.12E-6 7.08 4.63

25 1.1381 1.37E-6 7.81 4.18

30 1.1332 1.65E-6 8.59 3.78

35 1.1277 1.99E-6 9.42 3.44

40 1.1230 2.37E-6 10.28 3.13

45 1.1174 2.82E-6 11.19 2.87

50 1.1126 3.33E-6 12.13 2.64

55 1.1072 3.89E-6 13.10 2.43

60 1.1022 4.52E-6 14.08 2.26

65 1.0971 5.18E-6 15.04 2.11

70 1.0919 5.86E-6 15.96 1.98

75 1.0870 6.54E-6 16.81 1.88

80 1.0815 7.16E-6 17.54 1.79

85 1.0768 7.68E-6 18.10 1.74

90 1.0713 8.04E-6 18.45 1.70

Table 1d

PC + EMC + 1 M NaClO4

T d D_Na conductivity viscosity

°C g∙cm-3 cm2∙s-1 mS∙cm-1 mPa∙s

0 1.1927 6.41E-7 6.21 5.64

5 1.1874 7.98E-7 6.94 5.03

10 1.1825 9.83E-7 7.70 4.52

15 1.1769 1.20E-6 8.49 4.08

20 1.1722 1.45E-6 9.33 3.70

25 1.1666 1.74E-6 10.21 3.37

30 1.1620 2.08E-6 11.14 3.08

35 1.1564 2.47E-6 12.11 2.82

40 1.1517 2.92E-6 13.13 2.60

45 1.1461 3.44E-6 14.19 2.39

50 1.1415 4.01E-6 15.29 2.21

55 1.1362 4.64E-6 16.41 2.06

60 1.1313 5.33E-6 17.54 1.93

65 1.1258 6.05E-6 18.64 1.81

70 1.1211 6.81E-6 19.68 1.71

75 1.1155 7.52E-6 20.62 1.63

80 1.1107 8.17E-6 21.42 1.57

85 1.1054 8.69E-6 22.01 1.52

90 1.1008 9.03E-6 22.35 1.50

Table 1e

PC + EC + 1 M NaClO4

T d D_Na conductivity viscosity

°C g∙cm-3 cm2∙s-1 mS∙cm-1 mPa∙s

0 1.3502 2.87E-7 3.98 11.41

5 1.3454 3.92E-7 4.69 9.66

10 1.3397 5.24E-7 5.45 8.29

15 1.3350 6.87E-7 6.26 7.19

20 1.3297 8.85E-7 7.13 6.31

25 1.3245 1.13E-6 8.05 5.57

30 1.3198 1.41E-6 9.02 4.96

35 1.3143 1.76E-6 10.05 4.43

40 1.3094 2.17E-6 11.12 4.00

45 1.3047 2.63E-6 12.25 3.63

50 1.2990 3.16E-6 13.40 3.31

55 1.2943 3.77E-6 14.58 3.03

60 1.2894 4.43E-6 15.76 2.80

65 1.2839 5.14E-6 16.91 2.60

70 1.2792 5.87E-6 18.00 2.45

75 1.2745 6.60E-6 18.99 2.31

80 1.2693 7.25E-6 19.83 2.21

85 1.2641 7.81E-6 20.47 2.14

90 1.2594 8.18E-6 20.84 2.11

Table 1f

PC + G1 + 1 M NaClO4

T d D_Na conductivity viscosity

°C g∙cm-3 cm2∙s-1 mS∙cm-1 mPa∙s

0 1.1164 1.15E-6 11.70 3.34

5 1.1107 1.40E-6 12.84 3.04

10 1.1054 1.68E-6 14.01 2.78

15 1.1004 2.00E-6 15.23 2.55

20 1.0947 2.37E-6 16.51 2.34

25 1.0897 2.79E-6 17.85 2.16

30 1.0842 3.26E-6 19.25 2.00

35 1.0787 3.81E-6 20.71 1.85

40 1.0741 4.44E-6 22.23 1.72

45 1.0685 5.13E-6 23.81 1.61

50 1.0631 5.91E-6 25.43 1.50

55 1.0575 6.75E-6 27.07 1.41

60 1.0520 7.66E-6 28.69 1.33

65 1.0469 8.61E-6 30.27 1.26

70 1.0413 9.56E-6 31.75 1.19

75 1.0357 1.04E-5 33.05 1.15

80 1.0311 1.13E-5 34.13 1.11

85 1.0255 1.19E-5 34.88 1.08

90 1.0199 1.23E-5 35.22 1.08

Table 1g

PC + G2 + 1 M NaClO4

T d D_Na conductivity viscosity

°C g∙cm-3 cm2∙s-1 mS∙cm-1 mPa∙s

0 1.1369 4.15E-7 4.74 8.29

5 1.1323 5.33E-7 5.41 7.24

10 1.1267 6.74E-7 6.12 6.37

15 1.1211 8.44E-7 6.87 5.65

20 1.1164 1.05E-6 7.68 5.03

25 1.1109 1.30E-6 8.53 4.51

30 1.1060 1.58E-6 9.45 4.05

35 1.1008 1.92E-6 10.42 3.67

40 1.0957 2.32E-6 11.45 3.32

45 1.0906 2.79E-6 12.54 3.02

50 1.0853 3.33E-6 13.68 2.76

55 1.0806 3.94E-6 14.85 2.53

60 1.0750 4.62E-6 16.05 2.34

65 1.0703 5.35E-6 17.24 2.17

70 1.0649 6.13E-6 18.39 2.03

75 1.0593 6.91E-6 19.46 1.91

80 1.0544 7.64E-6 20.40 1.82

85 1.0493 8.29E-6 21.15 1.75

90 1.0441 8.76E-6 21.67 1.71

Table 1h

PC + G4 + 1 M NaClO4

T d D_Na conductivity viscosity

°C g∙cm-3 cm2∙s-1 mS∙cm-1 mPa∙s

0 1.1582 1.32E-7 1.55 26.74

5 1.1532 1.81E-7 1.88 21.98

10 1.1480 2.44E-7 2.24 18.35

15 1.1428 3.23E-7 2.63 15.51

20 1.1378 4.20E-7 3.05 13.25

25 1.1324 5.40E-7 3.51 11.44

30 1.1276 6.87E-7 4.01 9.95

35 1.1220 8.65E-7 4.54 8.71

40 1.1173 1.08E-6 5.11 7.68

45 1.1119 1.34E-6 5.71 6.81

50 1.1070 1.63E-6 6.34 6.08

55 1.1017 1.98E-6 6.99 5.47

60 1.0966 2.38E-6 7.65 4.95

65 1.0915 2.81E-6 8.31 4.51

70 1.0862 3.27E-6 8.95 4.16

75 1.0814 3.75E-6 9.55 3.87

80 1.0759 4.21E-6 10.08 3.65

85 1.0713 4.63E-6 10.50 3.47

90 1.0658 4.94E-6 10.79 3.36

Table 1i

PC + SL + 1 M NaClO4

T d D_Na conductivity viscosity

°C g∙cm-3 cm2∙s-1 mS∙cm-1 mPa∙s

0 1.3255 1.44E-7 1.88 24.82

5 1.3208 1.96E-7 2.24 20.80

10 1.3153 2.63E-7 2.63 17.68

15 1.3104 3.46E-7 3.06 15.19

20 1.3056 4.49E-7 3.53 13.17

25 1.2999 5.76E-7 4.03 11.51

30 1.2952 7.31E-7 4.57 10.13

35 1.2904 9.19E-7 5.15 8.97

40 1.2848 1.15E-6 5.77 8.00

45 1.2801 1.42E-6 6.43 7.16

50 1.2745 1.73E-6 7.12 6.45

55 1.2697 2.10E-6 7.84 5.85

60 1.2650 2.51E-6 8.58 5.33

65 1.2594 2.97E-6 9.33 4.90

70 1.2547 3.46E-6 10.05 4.54

75 1.2500 3.97E-6 10.74 4.24

80 1.2445 4.46E-6 11.35 4.01

85 1.2396 4.91E-6 11.86 3.84

90 1.2349 5.25E-6 12.22 3.72

Table 2a

Mixture density density conductivity conductivity DNa

g∙cm-3 g∙cm-3 mS∙cm-1 mS∙cm-1 10-7 cm2∙s-1

experimental AEM experimental AEM AEM

PC 1.2616 1.27 5.82 6.17 8.73

PC+DMC 1.2105 1.20 7.41 10.28 16.43

PC+DEC 1.1439 1.14 4.26 7.81 13.66

PC+EMC 1.1741 1.17 5.57 10.21 17.41

PC+DPrC 1.1726 ---- 3.63 ---- ---

PC+EC 1.3243 1.32 7.08 8.05 11.28

PC+12BC 1.2353 ---- 4.18 ---- ---

PC+G1 1.1082 1.09 10.00 17.85 27.86

PC+G2 1.1241 1.11 7.85 8.53 12.96

PC+G4 1.1363 1.13 3.21 3.51 5.40

PC+SL 1.2984 1.30 3.22 4.03 5.76

Table 2b

Mixture viscosity viscosity EA R2

mPa∙s mPa∙s kJ∙mol-1 for the linear fit

experimental AEM "based on experimental rheology data"

PC 7.27 7.02 19.8 0.9993

PC+DMC 4.08 3.56 13.0 0.9987

PC+DEC 4.08 4.18 16.6 0.9990

PC+EMC 3.85 3.37 10.6 0.9980

PC+DPrC 6.43 ---- 19.0 0.9979

PC+EC 6.84 5.57 19.0 0.9995

PC+12BC 8.32 ---- 21.5 0.9984

PC+G1 3.41 2.16 11.1 0.9976

PC+G2 4.33 4.51 16.3 0.9977

PC+G4 10.90 11.44 20.8 0.9988

PC+SL 14.72 11.51 23.1 0.9981

Table 3a

PC PC+DMC PC+DEC PC+EMC PC+DPrC PC+EC PC+12BC PC+G1 PC+G2 PC+G4 PC+SL

20 5.3 6.60 3.9 5.0 3.2 6.30 3.7 9.30 7.20 2.8 2.8

30 6.5 8.10 4.6 6.1 4.0 7.90 4.8 10.7 8.60 3.7 3.7

40 7.9 10.3 5.4 7.3 4.9 9.60 5.9 12.2 10.0 4.8 4.6

50 9.4 12.9 6.3 8.6 5.8 11.3 7.2 13.7 11.4 5.9 5.7

Table 3b

PC PC+DMC PC+DEC PC+EMC PC+DPrC PC+EC PC+12BC PC+G1 PC+G2 PC+G4 PC+SL

20 8.4 4.5 4.6 4.2 7.5 7.8 9.8 3.7 4.9 12.5 17.5

30 6.3 3.7 3.7 3.6 5.7 6.0 7.1 3.2 3.9 9.5 12.5

40 4.9 3.2 3.1 3.1 4.5 4.7 5.4 2.8 3.1 7.4 9.4

50 3.9 2.7 2.7 2.8 3.6 3.8 4.3 2.6 2.6 5.6 7.2

Table 3c

PC PC+DMC PC+DEC PC+EMC PC+DPrC PC+EC PC+12BC PC+G1 PC+G2 PC+G4 PC+SL

20 1.2626 1.2160 1.1492 1.1795 1.1776 1.3297 1.2403 1.1133 1.1289 1.1413 1.3033

30 1.2529 1.2045 1.1387 1.1687 1.1675 1.3189 1.2303 1.1032 1.1194 1.1315 1.2935

40 1.2422 1.1936 1.1282 1.1579 1.1574 1.3082 1.2202 1.0930 1.1098 1.1221 1.2839

50 1.2315 1.1824 1.1176 1.1470 1.1473 1.2974 1.2103 1.0828 1.1002 1.1129 1.2744

Table 4

Mixture "conductivity (T = 50 °C) / conductivity (T = 25 °C)" "viscosity (T = 50 °C) / viscosity (T = 25 °C)"

experimental experimental

PC 1.62 0.54

PC+DMC 1.74 0.66

PC+DEC 1.47 0.67

PC+EMC 1.54 0.73

PC+DPrC 1.60 0.56

PC+EC 1.60 0.56

PC+12BC 1.73 0.51

PC+G1 1.37 0.75

PC+G2 1.46 0.60

PC+G4 1.83 0.52

PC+SL 1.77 0.49

Table 5a

PC PC+DMC PC+DEC PC+EMC PC+DPrC PC+EC PC+12BC PC+G1 PC+G2 PC+G4 PC+SL

5.838E-5 6.269E-4 1.559E-4 3.102E-4 1.472E-4 4.172E-4 5.405E-5 0.004 4.65E-4 5.379E-5

2.193E-4 2.819E-4 9.172E-5 0.06 2.999E-5 2.859E-4 0.001 0.002 2.859E-4 5.64E-5

1.505E-4 3.046E-4 5.851E-5 0.213 4.076E-5 9.665E-5 4.49E-5 0.002 5.549E-5

8.757E-5 0.006 9.985E-5 2.51E-5 3.292E-5 6.165E-5 1.901E-4 3.457E-5

0.006 2.555E-4 6.61E-5 0.003 4.2E-5 3.068E-5 3.173E-5 0.002

4.393E-4 8.709E-5 5.335E-4 3.874E-5 0.002 7.315E-5

0.001 0.003 0.005 7.977E-5

0.004 1.384E-4 4.091E-4 5.651E-5

0.002 1.948E-4 0.003 4.366E-4

9.071E-5 1.068E-4 1.225E-4 3.675E-5

2.185E-4 1.814E-4 2.032E-4 5.668E-5

1.236E-4 1.735E-4 5.013E-5 0.001

0.002 9.668E-4 0.001

6E-4 7.303E-4 3.364E-4

4.066E-4 6.167E-4 8.603E-5

6.78E-5 2.639E-4 1.745E-4

7.485E-5 5.861E-5

3.67E-5 5.04E-4

2.894E-4 2.407E-4

4.764E-4 0.001

2.576E-4 6.783E-5

2.969E-5 4.54E-5

1.844E-4 2.642E-4

4.605E-4 1.161E-4

3.541E-4 3.898E-4

2.267E-4 3.762E-4

5.103E-4 1.457E-4

2.138E-4 5.109E-5

3.159E-5 0.001

1.393E-4 1.679E-4

1.799E-4 1.99E-4

1.966E-4 8.806E-5

1.39E-4 2.074E-4

1.672E-4 9.09E-5

5.989E-5 5.183E-5

4.342E-5

2.92E-5

5.393E-5

6.693E-5

2.309E-4

1.797E-4

3.246E-4

1.027E-4

2.399E-4

2.481E-4

3.852E-4

Table 5b

PC PC+DMC PC+DEC PC+EMC PC+DPrC PC+EC PC+12BC PC+G1 PC+G2 PC+G4 PC+SL

0.004 0.013 0.002 0.001 0.007 2.6E-4 0.002 8.644E-4 5.833E-4 2.541E-4 3.018E-4

0.008 0.008 0.008 0.015 5.293E-5 0.004 0.003 0.002 7.617E-4 0.004 0.006

0.007 0.003 0.005 0.064 7.026E-4 9.686E-4 0.003 0.01 0.004 3.651E-4 4.44E-4

4.571E-4 3.088E-4 0.001 0.008 0.004 0.002 2.545E-4 8.798E-5 3.777E-4 1.142E-4

1.214E-4 0.004 0.001 3.359E-4 7.343E-4 4.609E-4 0.004 4.828E-4 3.289E-4 3.927E-5

3.03E-4 2.303E-4 1.411E-4 0.001 4.554E-4 2.526E-4 2.622E-4 5.056E-4 1.418E-4 1.079E-4

4.075E-4 0.004 9.695E-4 0.265 0.011 4.993E-4 5.35E-4 7.952E-5

2.989E-4 0.002 0.004 8.199E-4 1.178E-4 1.99E-4 2.134E-4 1.285E-4

0.001 3.325E-4 1.88E-4 2.152E-4 6.132E-4 1.675E-4

0.002 9.993E-5 1.19E-4 1.537E-4 6.438E-5

4.207E-4 0.002 4.672E-4 3.501E-4

0.002 4.966E-4 1.362E-4 2.427E-4

6.764E-4 1.852E-4 1.096E-4 1.614E-4

8.161E-4 7.74E-4 1.665E-4

0.003 8.388E-4 1.407E-4

0.002 7.044E-4 1.729E-4

5.105E-4 0.004

0.001 2.906E-4

8.904E-4 1.318E-4

0.004

3.377E-4

3.832E-4

Table 5c

Mixture "Electrolyte over Na with NaClO4" "Electrolyte over Na without NaClO4"

PC 0.104 0.0013

PC+DMC 0.086 0.0013

PC+DEC 0.065 0.0011

PC+EMC 0.071 0.0014

PC+DPrC 0.064 0.0013

PC+EC 0.088 0.0016

PC+12BC 0.074 0.0045

PC+G1 0.058 0.0014

PC+G2 0.045 0.0012

PC+G4 0.046 0.0012

PC+SL 0.058 0.0014

Table 7

solvent[a] "impurity (identified)" "retention time (FID)" "retention time (MS)" RI[b] "RI from NIST[c] database" "FID area relative to solvent area[d]" "verification"

"[min, Peak maximum]" "[min, onset]" [%] "NIST / (Match/ 1000)" "Retention, pure compound [e]" "EI fragmentation, pure compound[f]" "mass fragmentation m/z in descending intensity order"

EMC DMC 2.89 2.99 615 620 5.7 907 x x 45 59 90 62 60

EMC DEC 4.54 4.64 781 767 19.0 924 x x 45 91 63 43 59

DPrC di-n-propyl ether 3.53 3.63 681 680 0.2 885 --- --- 43 41 73 102 58

DPrC sec-butyl propyl carbonate 6.82 6.93 1013 (996) 0.02 790 --- --- 57 41 45 44 56

12-BC 1,2-butanediol 5.13 5.23 839 (824) 0.1 830 x x 59 41 43 58 57

12-BC propylene carbonate 6.58 6.67 985 931 0.03 752 x x 57 44 43 87 58

12-BC ? 6.68 6.78 997 --- 0.04 --- --- --- 101 44 43 57 41

12-BC ? 6.80 6.90 1010 --- 0.02 --- --- --- 43 44 45 41 57

G1 1,4-dioxane 3.78 3.88 707 695 0.03 857 x x 88 58 43 57 87

G1 butylated hydroxytoluene 10.66 10.77 1519 1513 0.02 724 x x 205 57 41 145 220

G2 dimethoxyethane 3.22 3.32 649 643 0.01 879 x x 45 60 90 58 43

G2 1,4-dioxane 3.77 3.88 707 695 0.01 878 x x 88 58 43 57 87

G2 ? 6.77 6.86 1006 --- 0.03 --- --- --- 73 59 45 117 41

G2 ? 6.89 7.00 1022 --- 0.02 --- --- --- 59 73 72 43 41

G2 butylated hydroxytoluene 10.65 10.77 1519 1513 0.02 731 x x 205 41 57 220 145

G4 ? 6.80 6.92 1013 --- 0.01 --- --- --- 45 59 58 43 44

G4 Triethylene glycol monomethyl ether 8.52 8.63 1217 1223 0.01 824 x x 45 59 58 89 43

G4 Triglyme 8.56 8.67 1222 1232 0.07 848 --- --- 59 58 45 43 103

G4 ? 9.06 9.17 1287 --- 0.03 --- --- --- 59 45 43 58 44

G4 Diethylene glycol di-n-butyl ether 10.10 10.22 1437 (1442) 0.04 839 x x 57 41 56 45 75

G4 ? 10.92 11.03 1556 --- 0.05 --- --- --- 59 58 45 73 103

G4 ? 11.20 11.30 1594 --- 0.04 --- --- --- 59 45 147 103 58

G4 ? 12.71 12.82 1765 --- 0.1 --- 59 58 103 45 87

SL ? 8.84 8.95 1258 --- 0.05 --- --- --- 89 39 53 55 120

SL ? 10.91 11.03 1556 --- 0.01 --- --- --- 69 55 64 41 56

SL ? 11.96 12.07 1689 --- 0.02 --- --- --- 91 79 64 77 106

SL ? 12.09 12.21 1705 --- 0.01 --- --- --- 67 79 157 41 109

MTBE C4-alkene 1.82 1.95 --- --- --- --- --- --- 56 41 39 55 50

MTBE 2-Methoxy butane 2.71 2.81 593 (530) --- 931 --- --- 59 73 41 45 43

MTBE tert-butyl formate 5.37 5.47 862 --- 837 --- --- 57 41 59 56 87

n-C4 --- 1.89 1.97 --- --- --- --- --- --- --- --- --- ---

n-C5 --- 2.17 2.24 --- --- --- --- --- --- --- --- --- ---

n-C6 --- 2.75 2.85 --- --- --- --- --- --- --- --- --- ---

n-C7 --- 3.71 3.81 --- --- --- --- --- --- --- --- --- ---

n-C8 --- 4.74 4.83 --- --- --- --- --- --- --- --- --- ---

n-C9 --- 5.76 5.86 --- --- --- --- --- --- --- --- --- ---

n-C10 --- 6.70 6.81 --- --- --- --- --- --- --- --- --- ---

n-C11 --- 7.57 7.69 --- --- --- --- --- --- --- --- --- ---

n-C12 --- 8.40 8.50 --- --- --- --- --- --- --- --- --- ---

n-C13 --- 9.15 9.27 --- --- --- --- --- --- --- --- --- ---

n-C14 --- 9.86 9.97 --- --- --- --- --- --- --- --- --- ---

n-C15 --- 10.54 10.64 --- --- --- --- --- --- --- --- --- ---

n-C16 --- 11.23 11.34 --- --- --- --- --- --- --- --- --- ---

n-C17 --- 12.05 12.16 --- --- --- --- --- --- --- --- --- ---

n-C18 --- 13.07 13.17 --- --- --- --- --- --- --- --- --- ---

n-C19 --- 14.54 14.46 --- --- --- --- --- --- --- --- --- ---

n-C20 --- 16.04 16.13 --- --- --- --- --- --- --- --- --- ---

[a] All alkanes mentioned are saturated n-alkanes with chain length of n carbon atoms measured from standards (Restek or Sigma Aldrich)

[b] RI values are provided based on n-alkanes according to the method described in the manuscript, namely equation (1).

[c] Retention index are taken from NIST database as experimental standard non-polar or semi-standard non-polar data. Data in brackets are estimated values for non-polar retention indices.

[d] Only given for a very rough estimation. It must be mentioned that relative FID area comparison is not suitable for quantifications. In a comparison with known concentrations of similar substance types (organic carbonates, ethers), we obtain response factors that differ by a factor of up to 2.

[e] Measured with same retention time in MS onset, x = confirmed.

[f] Measured with same EI fragmentation (same 5 main peaks with matching ratio), x = confirmed.

Table 8

solvent mixtures "compound (identified)" "retention time (FID)" "retention time (MS)" RI[a] "RI from NIST database" verification

[min, Peak maximum] [min, onset] "NIST / (Match/ 1000)" "Retention, pure compound [b]" "EI fragmentation, pure compound [c]" "mass fragmentation m/z in descending intensity order"

PC Propylene oxide 2.12 2.21 489 460 828 x x 58 43 57 39 42

PC 1,2-Propanediol 4.04 4.16 734 740 886 x x 45 43 61 44 58

PC Isopropyl isobutyrate 4.63 4.75 792 784 877 x x 43 71 41 89 115

PC Diisopropylcarbonate 5.43 5.53 868 (832)[e] 854 x x 43 45 63 104 59

PC+DMC Propylene oxide 2.12 2.21 489 460 876 x x 58 43 57 39 42

PC+DMC 1,2-Propanediol 4.04 4.16 734 740 891 x x 45 43 61 44 58

PC+DMC ?1 6.25 6.37 954 --- --- --- --- 45 59 72 73 103

PC+DMC A 8.37 8.48 1198 --- ---[d] --- --- 59 44 103 45 74

PC+DMC ?2 10.47 10.59 1493 --- --- --- --- 117 59 44 45 73

PC+DEC Ethanol 1.99 2.07 437 427 965 x x 45 46 43 --- ---

PC+DEC Propylene oxide 2.12 2.20 489 460 892 x x 58 43 57 39 42

PC+DEC 1,2-Propanediol 4.04 4.16 734 740 864 x x 45 43 61 44 58

PC+DEC ethyl propyl carbonate 5.01 5.12 828 861 725 --- --- 45 43 59 63 90

PC+DEC C 9.42 9.55 1340 1348 848 --- --- 44 45 59 58 104

PC+DEC ?3 11.39 11.50 1620 --- --- --- --- 131 103 59 45 91

PC+EMC Propylene oxide 2.11 2.20 485 460 894 x x 58 43 57 39 42

PC+EMC DMC 2.89 3.00 616 620 940 x x 45 59 90 62 60

PC+EMC 1,2-Propanediol 4.04 4.16 437 740 879 x x 45 43 61 44 58

PC+EMC DEC 4.57 4.65 782 767 931 x x 91 45 63 59 75

PC+EMC A 8.37 8.48 1198 --- ---[d] --- --- 59 44 103 45 74

PC+EMC B 8.92 9.03 1269 --- ---[d] --- --- 44 45 59 90 103

PC+EMC C 9.42 9.55 1340 1348 809 --- --- 44 45 59 58 104

PC+EMC ?2 10.47 10.59 1493 --- --- --- --- 117 59 73 45 118

PC+EMC ?4 10.91 11.03 1556 --- --- --- --- 117 59 131 45 103

PC+EMC ?3 11.39 11.51 1621 --- --- --- --- 103 131 59 45 91

PC+DPrC Propylene oxide 2.12 2.21 489 460 899 x x 58 43 57 39 42

PC+DPrC Di-n-propyl ether 3.53 3.64 691 680 802 --- --- 43 41 73 102 42

PC+DPrC n-Propyl acetate 3.80 3.90 709 708 916 --- --- 43 61 73 42 59

PC+DPrC 1,2-Propanediol 4.04 4.16 734 740 869 x x 45 43 61 44 58

PC+DPrC 5.95 6.06 921 --- --- --- --- 43 45 59 63 41

PC+DPrC ?5 8.86 8.99 1264 --- --- --- --- 43 58 59 100 41

PC+DPrC D 10.66 10.76 1517 (1537)[e] 828 --- --- 103 44 59 43 145

PC+DPrC ?6 13.08 13.18 1801 --- --- --- --- 103 44 59 145 43

PC+EC Propylene oxide 2.11 2.22 485 460 841 x x 58 43 57 39 42

PC+EC Ethylene glycol 3.56 3.67 694 702 723 x x 43 62 42 61 33

PC+EC ?7 3.89 4.00 719 --- --- --- --- 103 88 58 118 101

PC+EC 1,2-Propanediol 4.04 4.16 734 740 868 x x 45 43 61 57 42

PC+EC ?8 4.25 4.36 754 --- --- --- --- 117 89 87 118 59

PC+EC Diglyme 6.18 6.29 945 951 809 x x 59 58 45 89 87

PC+12BC Propylene oxide 2.11 2.20 485 460 841 x x 58 43 57 39 42

PC+12BC 1,2-Propanediol 4.04 4.16 734 740 870 x x 45 43 61 44 58

PC+12BC 1,2-Butanediol 5.13 5.24 788 832 x x 59 41 43 61 58

PC+12BC Diisopropyl carbonate 5.43 5.53 868 (832)[e] 840 --- --- 43 45 41 104 63

PC+12BC ?9 15.30 15.40 1956 --- --- --- --- 91 107 79 77 151

PC+G1 1,2-Propanediol 4.04 4.15 734 740 890 x x 45 43 61 58 57

PC+G1 ?8 4.25 4.34 752 117 89 59 88 87

PC+G1 Diisopropyl carbonate 5.43 5.53 868 (832)[e] 824 43 45 41 59 63

PC+G2 1,2-Propanediol 4.04 4.15 734 740 891 x x 45 43 61 58 57

PC+G4 1,2-Propanediol 4.04 4.15 734 740 874 x x 45 43 61 58 57

PC+G4 ?8 4.25 4.34 752 --- --- --- --- 117 89 59 88 87

PC+G4 Triglyme 8.58 8.69 1225 1232 854 --- --- 59 58 45 103 89

PC+G4 ?9 9.07 9.17 1287 --- --- --- --- 59 45 58 87 73

PC+G4 Diethylene glycol dibutyl ether 10.10 10.22 1437 (1442)[e] 788 x x 57 41 45 75 85

PC+SL 1,2-Propanediol 4.04 4.16 734 740 873 x x 45 43 61 57 42

[a] RI values are provided based on n-alkanes according to the method described in the manuscript, namely equation (1).

[b] Measured with same retention time in MS onset, x = confirmed; otherwise (---) no measurements of the pure substance was performed

[c] measured and EI fragmentation give same 5 main peaks with matching ratio), x = confirmed.

[d] not included in NIST data base.

[e] estimated RI index from NIST (no experimental value)

Table 9

Indicator PC+G1 PC+EC PC+DPrC PC+SL PC+G4 PC+DMC PC+EMC PC PC+G2 PC +1,2-BC PC+DEC Unit

CED 8.01E+01 7.84E+01 8.88E+01 8.91E+01 6.75E+01 7.96E+01 9.99E+01 9.34E+01 7.78E+01 8.62E+01 1.11E+02 MJ

Fine particulate matter formation 5.98E-03 6.20E-03 6.39E-03 1.07E-02 4.58E-03 6.05E-03 6.91E-03 7.64E-03 5.13E-03 6.52E-03 7.11E-03 kg PM2.5 eq

Fossil resource scarcity 1.58E+00 1.51E+00 1.71E+00 1.79E+00 1.33E+00 1.54E+00 1.88E+00 1.77E+00 1.53E+00 1.64E+00 2.05E+00 kg oil eq

Freshwater ecotoxicity 1.84E-01 2.07E-01 1.98E-01 1.60E-01 1.40E-01 2.10E-01 2.30E-01 2.46E-01 1.59E-01 2.07E-01 2.22E-01 kg 1,4-DCB

Freshwater eutrophication 1.40E-03 1.53E-03 1.65E-03 1.25E-03 1.09E-03 1.53E-03 2.01E-03 1.99E-03 1.25E-03 1.76E-03 2.28E-03 kg P eq

Global warming 3.70E+00 3.93E+00 4.06E+00 3.38E+00 2.85E+00 3.86E+00 4.12E+00 4.77E+00 3.26E+00 4.26E+00 4.05E+00 kg CO2 eq

Human carcinogenic toxicity 2.70E-01 2.92E-01 2.88E-01 2.48E-01 2.04E-01 2.94E-01 3.62E-01 3.51E-01 2.31E-01 2.99E-01 3.32E-01 kg 1,4-DCB

Human non-carcinogenic toxicity 4.12E+00 4.50E+00 4.49E+00 3.76E+00 3.27E+00 4.52E+00 5.11E+00 5.39E+00 3.64E+00 4.68E+00 5.18E+00 kg 1,4-DCB

Ionizing radiation 3.80E-01 4.43E-01 5.19E-01 3.65E-01 3.28E-01 4.50E-01 7.10E-01 6.09E-01 3.78E-01 5.66E-01 8.79E-01 kBq Co-60 eq

Land use 4.25E-02 4.87E-02 6.98E-02 3.78E-02 3.18E-02 4.86E-02 6.68E-02 6.18E-02 3.64E-02 6.74E-02 6.36E-02 m2a crop eq

Marine ecotoxicity 2.39E-01 2.67E-01 2.57E-01 2.07E-01 1.81E-01 2.71E-01 2.99E-01 3.18E-01 2.06E-01 2.70E-01 2.89E-01 kg 1,4-DCB

Marine eutrophication 1.34E-04 1.59E-04 1.48E-04 1.26E-04 1.02E-04 1.54E-04 1.78E-04 2.09E-04 1.19E-04 2.04E-04 1.90E-04 kg N eq

Mineral resource scarcity 1.43E-02 1.57E-02 1.48E-02 1.27E-02 1.15E-02 1.60E-02 1.71E-02 1.76E-02 1.26E-02 1.58E-02 1.55E-02 kg Cu eq

Ozone formation, Human health 1.36E-02 1.52E-02 1.57E-02 1.96E-02 9.61E-03 1.45E-02 1.52E-02 2.23E-02 1.15E-02 1.44E-02 1.44E-02 kg NOx eq

Ozone formation, Terrestrial ecosystems 1.73E-02 1.97E-02 2.03E-02 2.72E-02 1.20E-02 1.87E-02 1.87E-02 3.02E-02 1.46E-02 1.82E-02 1.79E-02 kg NOx eq

Stratospheric ozone depletion 1.89E-06 2.02E-06 1.89E-06 2.00E-06 1.35E-06 1.97E-06 2.37E-06 3.04E-06 1.63E-06 2.20E-06 2.57E-06 kg CFC11 eq

Terrestrial acidification 1.24E-02 1.29E-02 1.46E-02 2.97E-02 9.90E-03 1.27E-02 1.53E-02 1.56E-02 1.10E-02 1.47E-02 1.61E-02 kg SO2 eq

Terrestrial ecotoxicity 5.65E+00 7.39E+00 6.24E+00 5.39E+00 3.95E+00 7.04E+00 8.12E+00 8.50E+00 4.48E+00 7.55E+00 6.20E+00 kg 1,4-DCB

Water consumption 7.43E-02 7.74E-02 8.91E-02 6.42E-02 5.52E-02 7.40E-02 7.53E-02 1.09E-01 6.42E-02 1.71E-01 7.81E-02 m3

Table 10

cycle "specific discharge capacity / mAh g-1"

1 100.965

2 101.545

3 99.613

4 100

5 99.903

6 99.903

7 96.55

8 96.711

9 96.71

10 96.711

11 96.394

12 96.203

13 96.108

14 96.203

15 96.108

16 95.916

17 96.012

18 95.82

19 95.725

20 95.629

21 95.725

22 95.725

23 95.686

24 95.265

25 95.342

26 95.38

27 95.38

28 95.342

29 95.035

30 94.844

31 95.035

32 94.997

33 94.767

34 94.768

35 94.938

36 94.777

37 94.777

38 94.776

39 94.615

40 94.615

41 94.616

42 94.131

43 94.293

44 94.293

45 93.906

46 93.81

47 93.523

48 93.619

49 93.523

50 93.235

51 93.332

52 93.235

53 93.331

54 93.045

55 92.949

56 92.853

57 92.853

58 92.757

59 92.566

60 92.661

61 92.661

62 92.47

63 92.566

64 92.374

65 92.183

66 92.183

67 92.279

68 91.704

69 91.896

70 91.991

71 91.704

72 91.8

73 91.417

74 91.608

75 91.321

76 91.417

77 91.321

78 91.13

79 90.938

80 91.034

81 91.034

82 91.034

83 90.555

84 90.747

85 90.938

86 90.843

87 90.747

88 90.555

89 90.556

90 90.554

91 89.981

92 90.46

93 90.077

94 90.077

95 89.981

96 90.077

97 89.79

98 89.981

99 89.789

100 89.503

101 89.598

102 89.503

103 89.503

104 89.503

105 89.215

106 89.407

107 89.215

108 89.215

109 89.216

110 89.024

111 89.024

112 89.024

113 88.928

114 88.832

115 88.545

116 88.832

117 88.737

118 88.737

119 88.545

120 88.258

121 88.449

122 88.45

123 88.162

124 88.067

125 88.067

126 88.068

127 88.162

128 87.779

129 87.971

130 87.588

131 87.684

132 87.588

133 87.588

134 87.301

135 87.397

136 87.588

137 87.397

138 87.301

139 87.397

140 87.014

141 87.109

142 87.014

143 87.014

144 87.013

145 88.067

146 87.971

147 87.683

148 87.301

149 87.397

150 87.301

151 87.109

152 87.109

153 86.631

154 86.822

155 86.535

156 86.535

157 86.343

158 86.439

159 86.343

160 86.343

161 85.865

162 85.769

163 85.961

164 85.769

165 85.578

166 85.482

167 85.386

168 85.482

169 85.099

170 85.291

171 85.291

172 85.195

173 84.906

174 84.716

175 84.812

176 84.812

177 84.525

178 84.333

179 84.525

180 84.333

181 84.429

182 84.333

183 84.237

184 84.142

185 84.142

186 83.855

187 83.95

188 83.855

189 83.568

190 83.759

191 83.472

192 83.472

193 83.472

194 83.472

195 83.567

196 83.089

197 82.993

198 82.993

199 82.898

200 82.993

201 83.089

202 82.419

203 82.706

204 82.419

205 82.61

206 82.61

207 82.323

208 82.323

209 82.419

210 82.132

211 82.227

212 82.419

213 81.749

214 81.749

215 81.844

216 81.749

217 81.558

218 81.653

219 81.94

220 81.749

221 82.036

222 81.366

223 81.174

224 81.174

225 81.653

226 81.653

227 81.078

228 81.27

229 80.887

230 81.079

231 81.27

232 80.792

233 80.696

234 80.6

235 81.174

236 81.079

237 80.887

238 80.504

239 80.217

240 80.696

241 80.504

242 80.504

243 80.696

244 80.504

Table 11

"Time / min" "Temperature / °C" "DSC / mW/mg" "Mass / %" "Gas Flow (purge1) / (ml/min)" "Gas Flow (protective) / (ml/min)" "Sensit./(uV/mW)"

0 21.715 0.003 100 50 20 1.016

0.25 21.716 0.004 99.992 50 20 1.016

0.5 21.801 0.005 99.994 50 20 1.016

0.75 22.037 0.009 99.992 50 20 1.016

1 22.372 0.014 99.972 50 20 1.016

1.25 22.892 0.021 99.988 50 20 1.015

1.5 23.555 0.028 99.967 50 20 1.015

1.75 24.288 0.035 99.973 50 20 1.014

2 25.136 0.042 99.972 50 20 1.013

2.25 26.078 0.048 99.964 50 20 1.012

2.5 27.119 0.053 99.928 50 20 1.011

2.75 28.287 0.058 99.953 50 20 1.01

3 29.614 0.063 99.937 50 20 1.009

3.25 31.06 0.068 99.953 50 20 1.008

3.5 32.622 0.073 99.939 50 20 1.006

3.75 34.319 0.078 99.944 50 20 1.005

4 36.182 0.083 99.919 50 20 1.003

4.25 38.162 0.087 99.912 50 20 1.002

4.5 40.27 0.09 99.916 50 20 1

4.75 42.472 0.093 99.882 50 20 0.998

5 44.796 0.095 99.9 50 20 0.996

5.25 47.267 0.096 99.887 50 20 0.993

5.5 49.816 0.097 99.882 50 20 0.991

5.75 52.48 0.096 99.826 50 20 0.989

6 55.23 0.095 99.844 50 20 0.986

6.25 58.088 0.094 99.851 50 20 0.984

6.5 61.012 0.092 99.837 50 20 0.981

6.75 64.006 0.089 99.781 50 20 0.978

7 67.113 0.086 99.818 50 20 0.976

7.25 70.25 0.082 99.746 50 20 0.973

7.5 73.445 0.078 99.748 50 20 0.97

7.75 76.66 0.073 99.723 50 20 0.967

8 79.935 0.069 99.689 50 20 0.964

8.25 83.248 0.064 99.619 50 20 0.961

8.5 86.632 0.058 99.566 50 20 0.958

8.75 90.025 0.052 99.54 50 20 0.955

9 93.434 0.044 99.446 50 20 0.952

9.25 96.867 0.036 99.387 50 20 0.949

9.5 100.333 0.028 99.288 50 20 0.946

9.75 103.777 0.019 99.208 50 20 0.943

10 107.21 0.01 99.065 50 20 0.94

10.25 110.626 0.002 98.936 50 20 0.936

10.5 114.038 -0.006 98.772 50 20 0.933

10.75 117.456 -0.013 98.567 50 20 0.93

11 120.823 -0.019 98.36 50 20 0.927

11.25 124.187 -0.025 98.113 50 20 0.924

11.5 127.575 -0.028 97.812 50 20 0.921

11.75 130.914 -0.029 97.46 50 20 0.918

12 134.239 -0.029 97.069 50 20 0.915

12.25 137.517 -0.026 96.595 50 20 0.912

12.5 140.775 -0.021 96.076 50 20 0.909

12.75 144.018 -0.014 95.449 50 20 0.907

13 147.221 -0.003 94.758 50 20 0.904

13.25 150.376 0.013 93.885 50 20 0.901

13.5 153.494 0.034 92.936 50 20 0.898

13.75 156.562 0.061 91.727 50 20 0.895

14 159.639 0.095 90.422 50 20 0.893

14.25 162.652 0.133 88.879 50 20 0.89

14.5 165.654 0.174 87.115 50 20 0.887

14.75 168.578 0.228 85.062 50 20 0.885

15 171.485 0.275 82.839 50 20 0.882

15.25 174.345 0.331 80.327 50 20 0.88

15.5 177.173 0.369 77.711 50 20 0.877

15.75 179.968 0.408 74.79 50 20 0.875

16 182.726 0.444 71.784 50 20 0.872

16.25 185.483 0.484 68.576 50 20 0.87

16.5 188.205 0.547 64.957 50 20 0.868

16.75 190.906 0.617 60.986 50 20 0.865

17 193.574 0.673 56.813 50 20 0.863

17.25 196.205 0.727 52.323 50 20 0.861

17.5 198.84 0.788 47.162 50 20 0.858

17.75 201.426 0.841 41.929 50 20 0.856

18 203.961 0.889 36.62 50 20 0.854

18.25 206.474 0.927 31.905 50 20 0.852

18.5 208.99 0.92 26.588 50 20 0.85

18.75 211.43 0.749 22.439 50 20 0.848

19 213.935 0.452 19.458 50 20 0.845

19.25 216.62 0.069 17.709 50 20 0.843

19.5 219.455 -0.267 17.67 50 20 0.841

19.75 222.345 -0.398 17.137 50 20 0.838

20 225.144 -0.441 16.649 50 20 0.836

20.25 227.844 -0.456 16.452 50 20 0.834

20.5 230.508 -0.455 16.301 50 20 0.831

20.75 233.131 -0.458 15.992 50 20 0.829

21 235.702 -0.457 15.692 50 20 0.827

21.25 238.21 -0.339 14.401 50 20 0.825

21.5 240.698 -0.412 13.896 50 20 0.823

21.75 243.186 -0.382 13.287 50 20 0.821

22 245.635 -0.322 12.334 50 20 0.819

22.25 248.016 -0.27 11.413 50 20 0.817

22.5 250.435 -0.167 10.171 50 20 0.815

22.75 252.917 -0.093 9.041 50 20 0.813

23 255.338 -0.068 8.24 50 20 0.811

23.25 257.798 -0.095 7.985 50 20 0.809

23.5 260.273 -0.121 7.945 50 20 0.807

23.75 262.75 -0.126 7.955 50 20 0.804

24 265.255 -0.127 7.932 50 20 0.802

24.25 267.742 -0.126 7.931 50 20 0.8

24.5 270.267 -0.108 7.916 50 20 0.798

24.75 272.767 -0.1 7.949 50 20 0.796

25 275.31 -0.1 7.93 50 20 0.794

25.25 277.823 -0.098 7.91 50 20 0.792

25.5 280.363 -0.103 7.913 50 20 0.79

25.75 282.901 -0.109 7.89 50 20 0.788

26 285.379 -0.109 7.879 50 20 0.786

26.25 287.862 -0.109 7.88 50 20 0.784

26.5 290.301 -0.108 7.879 50 20 0.782

26.75 292.741 -0.107 7.895 50 20 0.78

27 295.184 -0.106 7.887 50 20 0.778

27.25 297.632 -0.105 7.864 50 20 0.776

27.5 300.062 -0.102 7.912 50 20 0.774

27.75 302.485 -0.086 7.904 50 20 0.772

28 304.927 -0.096 7.885 50 20 0.771

28.25 307.385 -0.105 7.928 50 20 0.769

28.5 309.844 -0.107 7.932 50 20 0.767

28.75 312.273 -0.108 7.899 50 20 0.765

29 314.77 -0.109 7.903 50 20 0.763

29.25 317.214 -0.109 7.935 50 20 0.761

29.5 319.67 -0.109 7.865 50 20 0.759

29.75 322.127 -0.108 7.941 50 20 0.757

30 324.62 -0.108 7.936 50 20 0.755

30.25 327.061 -0.107 7.97 50 20 0.753

30.5 329.522 -0.107 7.966 50 20 0.751

30.75 331.987 -0.106 7.965 50 20 0.75

31 334.437 -0.106 7.978 50 20 0.748

31.25 336.927 -0.106 7.972 50 20 0.746

31.5 339.385 -0.106 7.943 50 20 0.744

31.75 341.854 -0.105 8.027 50 20 0.742

32 344.333 -0.105 7.977 50 20 0.74

32.25 346.793 -0.105 8.014 50 20 0.738

32.5 349.267 -0.105 8.001 50 20 0.737

32.75 351.732 -0.105 8.008 50 20 0.735

33 354.233 -0.106 7.983 50 20 0.733

33.25 356.715 -0.105 8.007 50 20 0.731

33.5 359.163 -0.104 8.07 50 20 0.729

33.75 361.652 -0.105 8.034 50 20 0.727

34 364.136 -0.105 8.053 50 20 0.726

34.25 366.627 -0.107 7.963 50 20 0.724

34.5 369.099 -0.106 8.032 50 20 0.722

34.75 371.584 -0.106 8.014 50 20 0.72

35 374.064 -0.107 7.982 50 20 0.718

35.25 376.546 -0.107 8.018 50 20 0.717

35.5 379.014 -0.106 8.048 50 20 0.715

35.75 381.516 -0.107 7.885 50 20 0.713

36 383.969 -0.106 8.104 50 20 0.711

36.25 386.46 -0.105 8.079 50 20 0.71

36.5 388.957 -0.104 8.041 50 20 0.708

36.75 391.425 -0.103 8.071 50 20 0.706

37 393.907 -0.102 8.161 50 20 0.704

37.25 396.389 -0.103 7.95 50 20 0.703

37.5 398.876 -0.103 8.002 50 20 0.701

37.75 401.373 -0.104 7.983 50 20 0.699

38 403.842 -0.103 7.968 50 20 0.697

38.25 406.339 -0.103 8.02 50 20 0.696

38.5 408.813 -0.104 7.974 50 20 0.694

38.75 411.292 -0.104 7.995 50 20 0.692

39 413.793 -0.104 7.976 50 20 0.691

39.25 416.28 -0.103 7.987 50 20 0.689

39.5 418.758 -0.104 7.902 50 20 0.687

39.75 421.258 -0.104 7.927 50 20 0.686

40 423.735 -0.104 7.909 50 20 0.684

40.25 426.221 -0.105 7.903 50 20 0.682

40.5 428.712 -0.104 7.917 50 20 0.681

40.75 431.198 -0.103 7.917 50 20 0.679

41 433.668 -0.102 7.926 50 20 0.677

41.25 436.174 -0.102 7.923 50 20 0.676

41.5 438.658 -0.1 7.956 50 20 0.674

41.75 441.151 -0.101 7.912 50 20 0.673

42 443.653 -0.099 7.94 50 20 0.671

42.25 446.121 -0.098 7.95 50 20 0.669

42.5 448.627 -0.097 7.913 50 20 0.668

42.75 451.114 -0.095 7.941 50 20 0.666

43 453.606 -0.093 7.923 50 20 0.665

43.25 456.092 -0.089 7.958 50 20 0.663

43.5 458.592 -0.086 7.921 50 20 0.661

43.75 461.095 -0.082 7.916 50 20 0.66

44 463.562 -0.075 7.934 50 20 0.658

44.25 466.048 -0.06 7.933 50 20 0.657

44.5 468.548 -0.018 7.952 50 20 0.655

44.75 471.012 0.066 7.939 50 20 0.654

45 473.457 0.159 7.93 50 20 0.652

45.25 475.95 0.067 7.97 50 20 0.651

45.5 478.476 0.048 7.976 50 20 0.649

45.75 480.994 0.041 7.966 50 20 0.648

46 483.468 0.039 7.942 50 20 0.646

46.25 485.991 0.039 7.945 50 20 0.645

46.5 488.484 0.04 7.984 50 20 0.643

46.75 490.995 0.041 7.932 50 20 0.642

47 493.504 0.04 7.947 50 20 0.64

47.25 496.007 0.042 7.996 50 20 0.639

47.5 498.5 0.042 7.978 50 20 0.637

47.75 500.995 0.042 7.908 50 20 0.636

48 503.485 0.043 7.932 50 20 0.634

48.25 505.979 0.051 7.92 50 20 0.633

48.5 508.491 0.05 7.893 50 20 0.632

48.75 510.96 0.053 7.931 50 20 0.63

49 513.471 0.048 7.927 50 20 0.629

49.25 515.973 0.047 7.897 50 20 0.627

49.5 518.462 0.046 7.925 50 20 0.626

49.75 520.964 0.046 7.899 50 20 0.624

50 523.452 0.046 7.881 50 20 0.623

50.25 525.947 0.044 7.86 50 20 0.622

50.5 528.459 0.043 7.86 50 20 0.62

50.75 530.946 0.042 7.848 50 20 0.619

51 533.457 0.04 7.816 50 20 0.618

51.25 535.943 0.038 7.852 50 20 0.616

51.5 538.442 0.035 7.791 50 20 0.615

51.75 540.935 0.034 7.754 50 20 0.614

52 543.432 0.032 7.735 50 20 0.612

52.25 545.92 0.029 7.712 50 20 0.611

52.5 548.419 0.026 7.686 50 20 0.61

52.75 550.922 0.019 7.544 50 20 0.608

53 553.441 0.009 7.503 50 20 0.607

53.25 555.92 -0.002 7.49 50 20 0.606

53.5 558.425 -0.015 7.349 50 20 0.604

53.75 560.93 -0.028 7.262 50 20 0.603

54 563.415 -0.043 7.151 50 20 0.602

54.25 565.923 -0.058 7.033 50 20 0.6

54.5 568.421 -0.073 6.861 50 20 0.599

54.75 570.941 -0.088 6.675 50 20 0.598

55 573.442 -0.103 6.455 50 20 0.597

55.25 575.938 -0.111 6.269 50 20 0.595

55.5 578.442 -0.126 5.969 50 20 0.594

55.75 580.948 -0.145 5.649 50 20 0.593

56 583.441 -0.255 5.331 50 20 0.592

56.25 585.974 -0.265 5.043 50 20 0.59

56.5 588.451 -0.259 4.77 50 20 0.589

56.75 590.968 -0.246 4.504 50 20 0.588

57 593.444 -0.23 4.294 50 20 0.587

57.25 595.931 -0.213 4.116 50 20 0.586

57.5 598.432 -0.196 3.95 50 20 0.584

57.75 600.913 -0.179 3.811 50 20 0.583

58 603.391 -0.162 3.756 50 20 0.582

58.25 605.885 -0.148 3.695 50 20 0.581

58.5 608.383 -0.137 3.627 50 20 0.58

58.75 610.875 -0.13 3.617 50 20 0.578

59 613.378 -0.127 3.602 50 20 0.577

59.25 615.879 -0.127 3.576 50 20 0.576

59.5 618.358 -0.129 3.559 50 20 0.575

59.75 620.859 -0.13 3.559 50 20 0.574

60 623.361 -0.131 3.524 50 20 0.573

60.25 625.835 -0.131 3.563 50 20 0.572

60.5 628.359 -0.132 3.562 50 20 0.57

60.75 630.844 -0.133 3.547 50 20 0.569

61 633.333 -0.135 3.544 50 20 0.568

61.25 635.851 -0.135 3.548 50 20 0.567

61.5 638.35 -0.136 3.535 50 20 0.566

61.75 640.841 -0.135 3.534 50 20 0.565

62 643.337 -0.135 3.549 50 20 0.564

62.25 645.849 -0.135 3.545 50 20 0.563

62.5 648.331 -0.135 3.492 50 20 0.562

62.75 650.824 -0.133 3.536 50 20 0.561

63 653.347 -0.133 3.527 50 20 0.56

63.25 655.823 -0.132 3.539 50 20 0.559

63.5 658.328 -0.131 3.536 50 20 0.557

63.75 660.809 -0.13 3.509 50 20 0.556

64 663.31 -0.128 3.531 50 20 0.555

64.25 665.812 -0.126 3.528 50 20 0.554

64.5 668.301 -0.124 3.54 50 20 0.553

64.75 670.807 -0.123 3.506 50 20 0.552

65 673.294 -0.12 3.526 50 20 0.551

65.25 675.806 -0.118 3.513 50 20 0.55

65.5 678.316 -0.115 3.531 50 20 0.549

65.75 680.814 -0.111 3.517 50 20 0.548

66 683.303 -0.108 3.501 50 20 0.547

66.25 685.786 -0.105 3.517 50 20 0.546

66.5 688.279 -0.101 3.499 50 20 0.545

66.75 690.779 -0.096 3.518 50 20 0.544

67 693.293 -0.092 3.521 50 20 0.543

67.25 695.799 -0.087 3.496 50 20 0.542

67.5 698.315 -0.083 3.499 50 20 0.541

67.75 700.797 -0.078 3.501 50 20 0.54

Table 12

Substance "European Community number" "Link to substance Infocard"

"Sodium Perchlorate" 231-511-9 https://echa.europa.eu/substance-information/-/substanceinfo/100.028.647

"Propylene Carbonate" 203-572-1 https://echa.europa.eu/substance-information/-/substanceinfo/100.003.248

"Ethylene Carbonate" 202-510-0 https://echa.europa.eu/substance-information/-/substanceinfo/100.002.283

"1,2 Butylene Carbonate" 403-780-4 https://echa.europa.eu/substance-information/-/substanceinfo/100.100.548

"Diethyl Carbonate" 203-311-1 https://echa.europa.eu/substance-information/-/substanceinfo/100.003.011

"Dimethyl Carbonate" 210-478-4 https://echa.europa.eu/substance-information/-/substanceinfo/100.009.527

"Ethylmethyl Carbonate" 433-480-9 https://echa.europa.eu/substance-information/-/substanceinfo/100.103.173

"Dipropyl Carbonate" 210-822-3 https://echa.europa.eu/substance-information/-/substanceinfo/100.009.839

Sulfolane 204-783-1 https://echa.europa.eu/substance-information/-/substanceinfo/100.004.349

Monoglyme 203-794-9 https://echa.europa.eu/substance-information/-/substanceinfo/100.003.451

Diglyme 203-924-4 https://echa.europa.eu/substance-information/-/substanceinfo/100.003.568

Tetraglyme 205-594-7 https://echa.europa.eu/substance-information/-/substanceinfo/100.005.086
